# Supplementary material for: Immune‐mediated ECM depletion improves tumour perfusion and payload delivery
Source: EMBO Mol Med. 2019 Nov 11;11(12):e10923. doi: 10.15252/emmm.201910923 (PMC6895610; doi:10.15252/emmm.201910923)
Supplement: Supplementary file 1 — Appendix [file EMMM-11-e10923-s001.pdf]

Table of Content Appendix

Appendix Table S1: Mean tumour stiffness/elasticity measured by OCT-microelastography

Appendix Fig S1. Phage library biopanning for peptides recognising tumour ECM and CSG binding specificity.

Appendix Fig S2. Purification of recombinant TNFα and TNFα-CSG, and their bioactivity in vitro and in vivo. **(A)** SDS-PAGE analysis indicating: M. Molecular weight markers, 1.

Appendix Fig S3. Immune-mediated effects of TNFα-CSG therapy.

Appendix Fig S4. TNFα-CSG therapy reduces hypoxia and lung metastasis.

Appendix Table S1: Mean tumour stiffness/elasticity measured by OCT-microelastography (on day 5) in response to consecutive daily i.v. injection of TNFα-CSG (2 μg) or CSG control (0.8 μg) for 4 days.

Tumour	Treatment	Mean [kPa]	± StDev
4T1	CSG 1	22.14	3.37
	CSG 2	10.49	3.15
	CSG 3	15.21	3.98
		15.95	3.383
4T1	TNFa-CSG 1	13.55	2.31
	TNFa-CSG 2	16.17	2.66
	TNFa-CSG 3	17.96	1.90
		15.89	1.281
RIP1-Tag5	CSG 1	20.53	2.91
	CSG 2	6.25	2.12
	CSG 3	6.12	2.68
		10.97	4.782
RIP1-Tag5	TNFa-CSG 1	5.17	1.86
	TNFa-CSG 2	8.81	1.95
	TNFa-CSG 3	3.19	1.78
		5.723	1.646

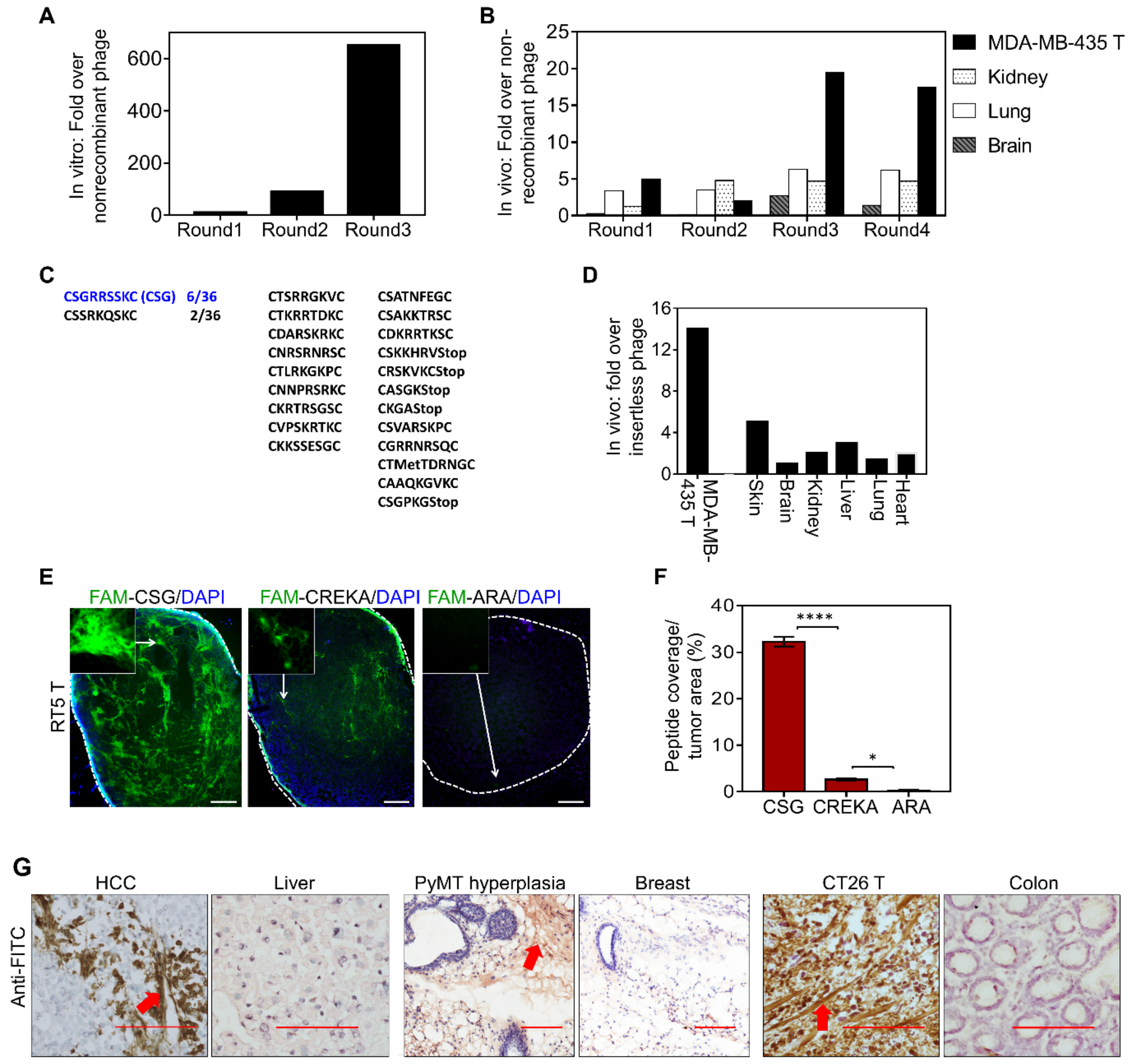

Appendix Fig S1. Phage library biopanning for peptides recognising tumour ECM and CSG binding specificity. **(A)** *In vitro* biopanning on Matrigel™ was performed using a cyclic peptide phage library encoding peptides with the general structure CX7C where X is any amino acid. Three rounds of selection yielded a phage pool that bound to Matrigel™ approximately 650-fold over control, non-recombinant phage. **(B)** The enriched phage pool from the third *in vitro* round was subsequently subjected to 4 rounds of *in vivo* screening in mice bearing MDA-MB-435 human breast cancer xenograft tumours (T). **(C)** Insert sequences of randomly picked phage colonies from the *in vivo*-selected phage pool. **(D)** The phage displaying the dominant CSGRRSSKC (CSG) peptide sequence was tested for homing to MDA-MB-435 tumours *in vivo*. **(E** and **F)** Acetone-fixed RIP1-Tag5 tumour tissue cross sections (RT5 T, 8 μm) were incubated with the indicated peptides (5 μM) for 20 min. Microscopic images show peptide binding (FAM^+, green), and the bar chart shows mean ± SEM of percent peptide coverage (FAM^+)/ tumour area (n=5 tumours; **P*<0.05, ****

P<0.0001 by one-way ANOVA test with Tukey correction). **(G)** FAM-CSG was i.v. injected into mice bearing tumours, and tissues were collected with perfusion after 1 h. Representative micrographs compare CSG binding detected by immunoperoxidase staining with anti-FITC antibody in ALB-Tag hepatocellular carcinoma (HCC) and non-transformed liver, early hyperplastic PyMT-MMTV breast carcinoma and normal breast tissue from a wildtype mouse, and transplant murine CT26 and normal colon. Scale bar: 100 µm. Arrows: CSG binding around tumour ECM.

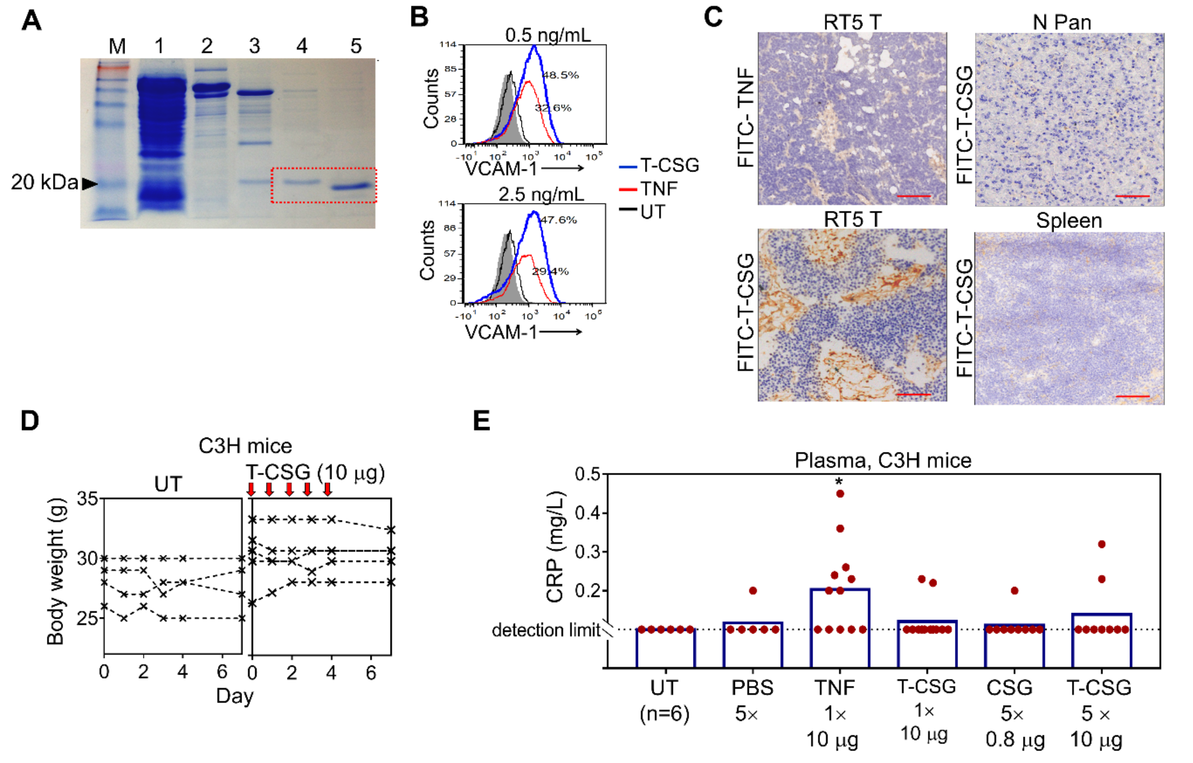

Appendix Fig S2. Purification of recombinant TNFα and TNFα-CSG, and their bioactivity in vitro and in vivo. **(A)** SDS-PAGE analysis indicating: M. Molecular weight markers, 1. Bacterial lysate, 2. His-Nus-tagged TNFα-CSG fusion protein purified using a Ni-NTA column, 3. TNFα-CSG cleaved off from His-NusA tag after TEV digestion, 4. Purified TNFα-CSG (18.9 kDa), and 5. Purified TNFα (17.7 kDa). **(B)** Brain endothelial cells (bEnd5) were incubated for 2 h with TNFα (TNF) and TNFα-CSG (T-CSG) at the indicated concentrations. Induction of VCAM-1 was analyzed by FACS. **(C)** RIP1-Tag5 mice (n=3 mice/group) were i.v. injected with 100 µg of FITC-labeled TNFα-CSG or TNFα. Tissues were collected 1 h later, and stained with anti-FITC-HRP antibody to detect the FITC-labeled proteins. Detection of the labeled proteins (brown) against haematoxylin staining (blue) in tumours (RT5 T) , normal pancreas (N Pan) and spleen. Scale bar: 100 µm. **(D)** Graph plots of body weight (g) of individual healthy C3H mouse before TNFα-CSG treatment (day 0) and daily during treatment until day 7. Body weights of untreated mice (UT) at indicated time points were compared in parallel. **(E)** Systemic toxicity profile of TNFα and TNFα-CSG assessed by i.v. injection of indicated compounds in normal C3H mice (9 - 11 weeks of age). Blood samples were collected retro-orbitally, 5 h after a single dose injection or 2 days after 5 daily doses. The levels of plasma C reactive protein (CRP) were measured as indicator of

inflammation. Elevated CRP levels in 7/12 mice treated with TNFα (58%) when compared to other groups (**P*<0.05, nonparametric test). The plasma CRP levels in both TNFα-CSG groups were not significantly different compared to the control PBS or CSG-treated groups (n=6 - 12 mice/group).

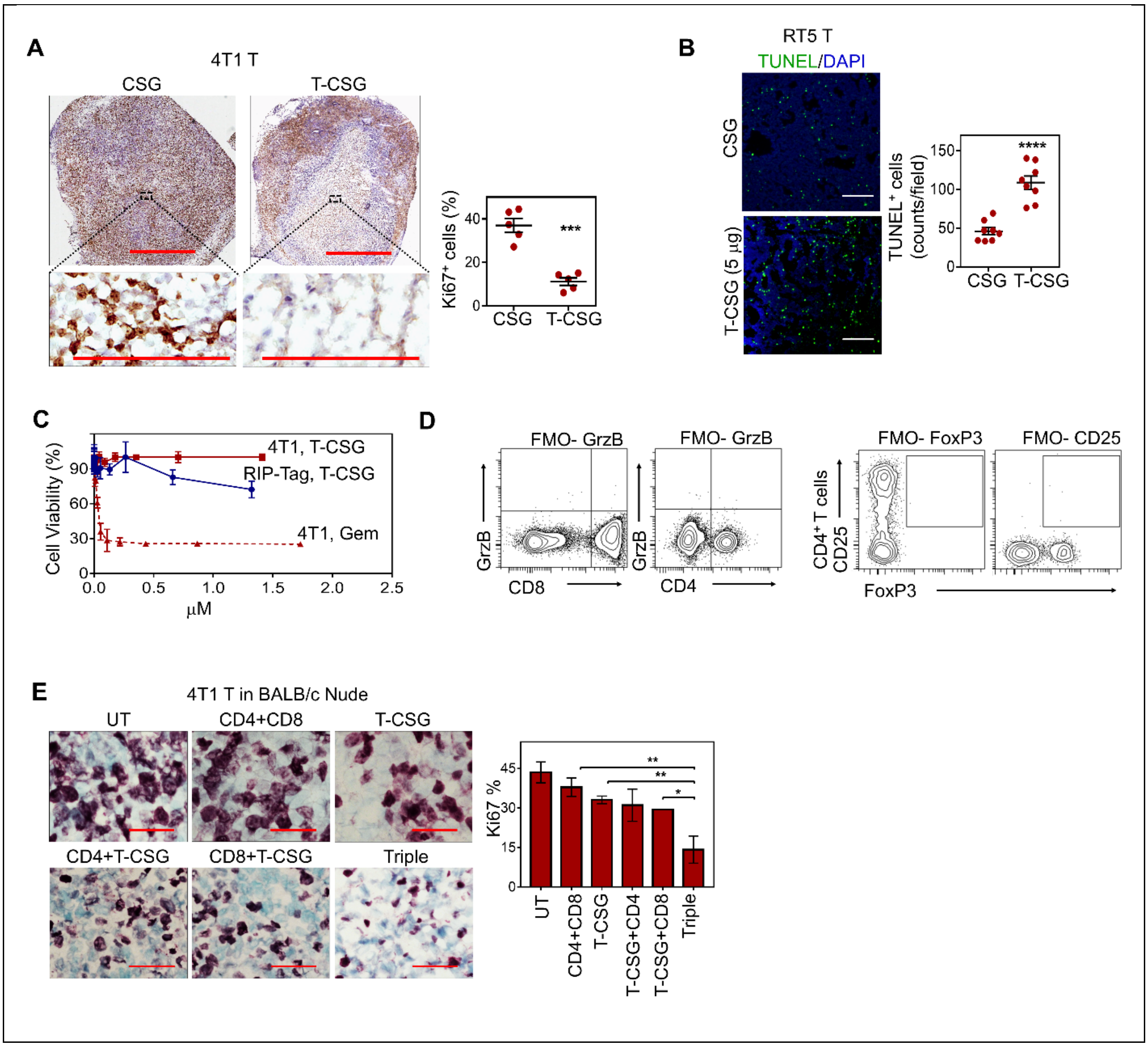

Appendix Fig S3. Immune-mediated effects of TNFα-CSG therapy. **(A)** Left: Ki67 staining (brown, counter-stained with hematoxylin) of 4T1 tumours in syngeneic BALB/c mice treated with indicated compounds. Scale bar: 2 mm (top) and 200 µm (bottom). Right: Quantification of Ki67$^+$ cells in individual tumours and mean ± SEM (n=5; ***P<0.001 by Student's t test). **(B)** Left: TUNEL staining of RIP1-Tag5 tumours treated with 5 daily injections of indicated compounds. Scale bar: 100 µm. Right: Quantification of mean TUNEL$^+$ cell counts in individual tumours and mean ± SEM (n=3; **P*<0.05 by Student's *t* test). **(C)** Cultured 4T1 tumour cells and malignant pancreatic beta cells derived from RIP-Tag mice were incubated in triplicates with indicated TNFα-CSG doses. Treatment of 4T1 tumour cells with gemcitabine (Gem) is shown as a control. MTT assays were performed after 48 h. **(D)** Gating strategies for FACS detection of cytotoxic (Granzyme B$^+$) CD8$^+$ and CD4$^+$ T cells and regulatory (FOXP3$^+$, CD25$^+$) CD4$^+$ T cells. All cells were gated on live CD3$^+$ population. **(E)** Comparison of Ki67 staining of 4T1 tumours in immunodeficient BALB/c Nude mice treated with TNFα-CSG alone or in combination with adoptive transfer of

$CD4^+$ or $CD8^+$ T cells, or both. Ki67 staining of tumours in the control untreated (UT) and combined CD4+ and CD8+ T cells were also compared. Treatment schedule for each agent is shown in Fig 8A. Left: Micrographs show $Ki67^+$ cells (purple) counter-stained with methyl green in tumours from the indicated groups. Scale bar: 40 μm. Right: Bar charts show mean ± SEM of $Ki67^+$ fractions/field (n=3; **P*<0.05 and ***P* <0.005 by one-way ANOVA test).

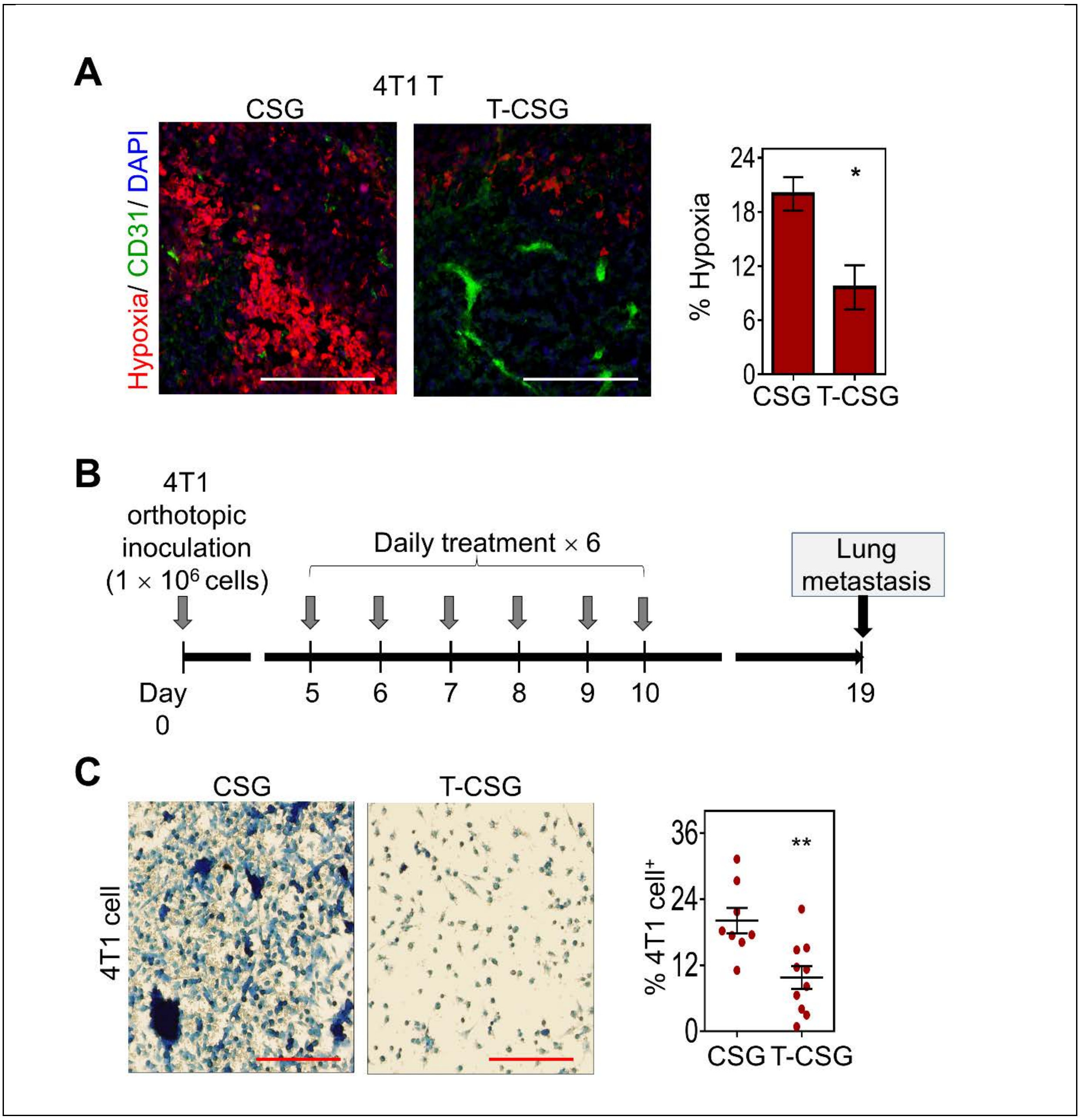

Appendix Fig S4. TNFα-CSG therapy reduces hypoxia and lung metastasis. **(A)** Left: Micrographs show tumour hypoxia (red) relative to blood vessels (CD31, green) in 4T1 tumours treated with 5 daily injections of indicated compounds. Scale bar: 50 μm. Right: The bar chart shows mean ± SEM of hypoxia^{+} staining (n=3; *P<0.05 by Student's t test). **(B)** Schedule depicting TNFα-CSG treatment and lung metastasis assay. Mice were inoculated orthotopically with 1 × 10^{6} 4T1 cells and treated with 6 daily doses of 10 μg TNFα-CSG or 0.8 μg CSG on day 5 post-inoculation. The mice were euthanised on day 19 post-inoculation and lungs were harvested for performing colonic assay (counts of colony outgrowth *in vitro* based on 4T1 cells resistance to 6-thioguanine). **(C)** Left: Representative micrographs of cell colonies stained with methylene blue. Scale bar: 100 μm. Right: Fractions of 4T1 cell^{+} /lung and mean ± SEM (n=8-10; **P<0.005 by Student's t test).
