# Supplementary material for: Immune‐mediated ECM depletion improves tumour perfusion and payload delivery
Source: EMBO Mol Med. 2019 Nov 11;11(12):e10923. doi: 10.15252/emmm.201910923 (PMC6895610; doi:10.15252/emmm.201910923)
Supplement: Supplementary file 2 — Expanded View Figures PDF [file EMMM-11-e10923-s002.pdf]

Expanded View Figures

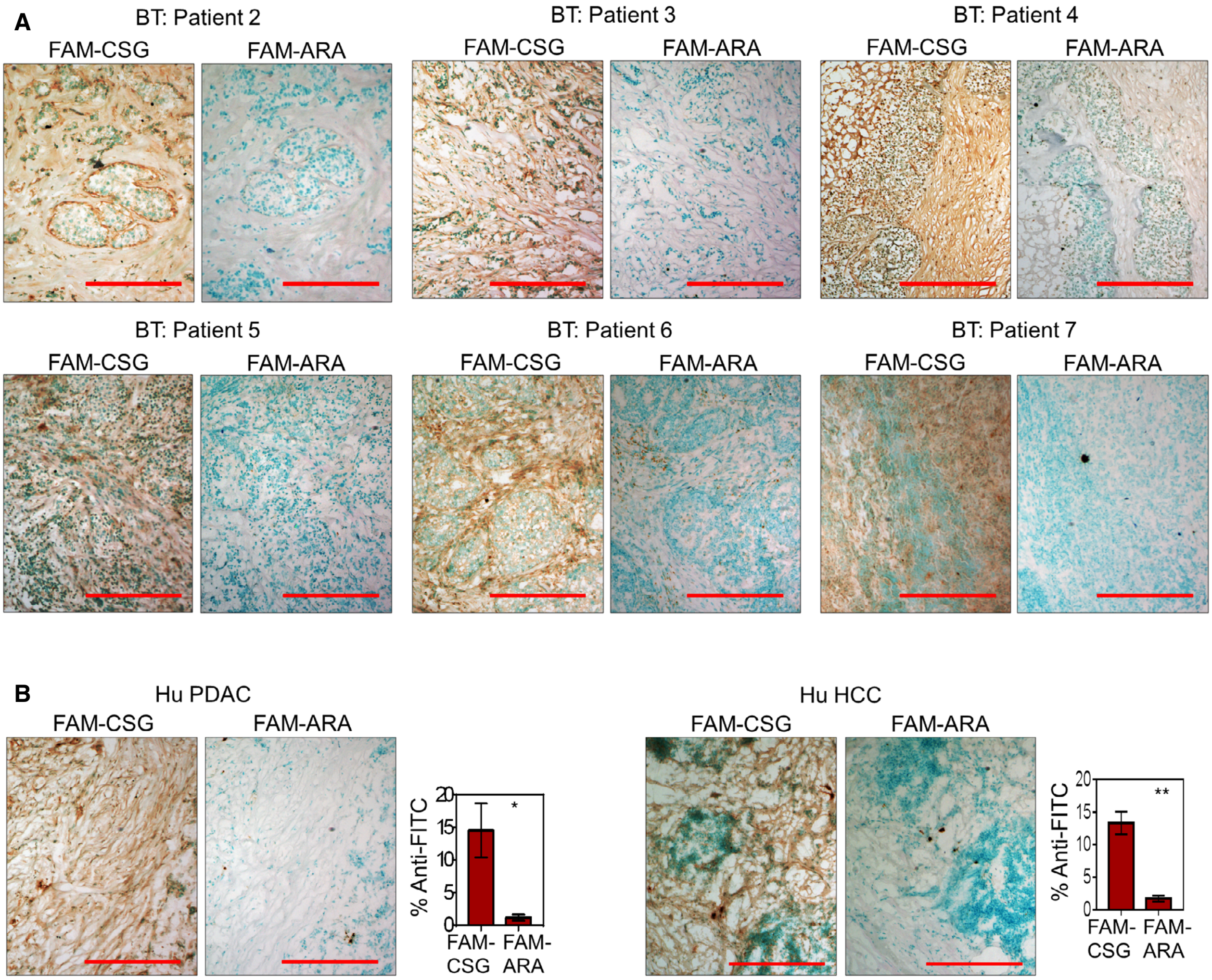

Figure EV1. CSG-specific binding in human cancers.

A, B 8-μm tissue sections were incubated for 30 min with 1 μM FAM-CSG or FAM-ARA. Micrographs of corresponding tissues stained with anti-FITC antibody (brown) are shown for (A) individual breast cancer patients and a representative (B, left) PDAC and (B, right) HCC patient samples. (B) Bar charts on the right show mean ± SEM of percentage area per tissue section stained with anti-FITC antibody ($N = 3$ PDAC and HCC patient samples; *$P < 0.05$ and **$P < 0.005$ by Student's *t*-test).

© 2019 The Authors

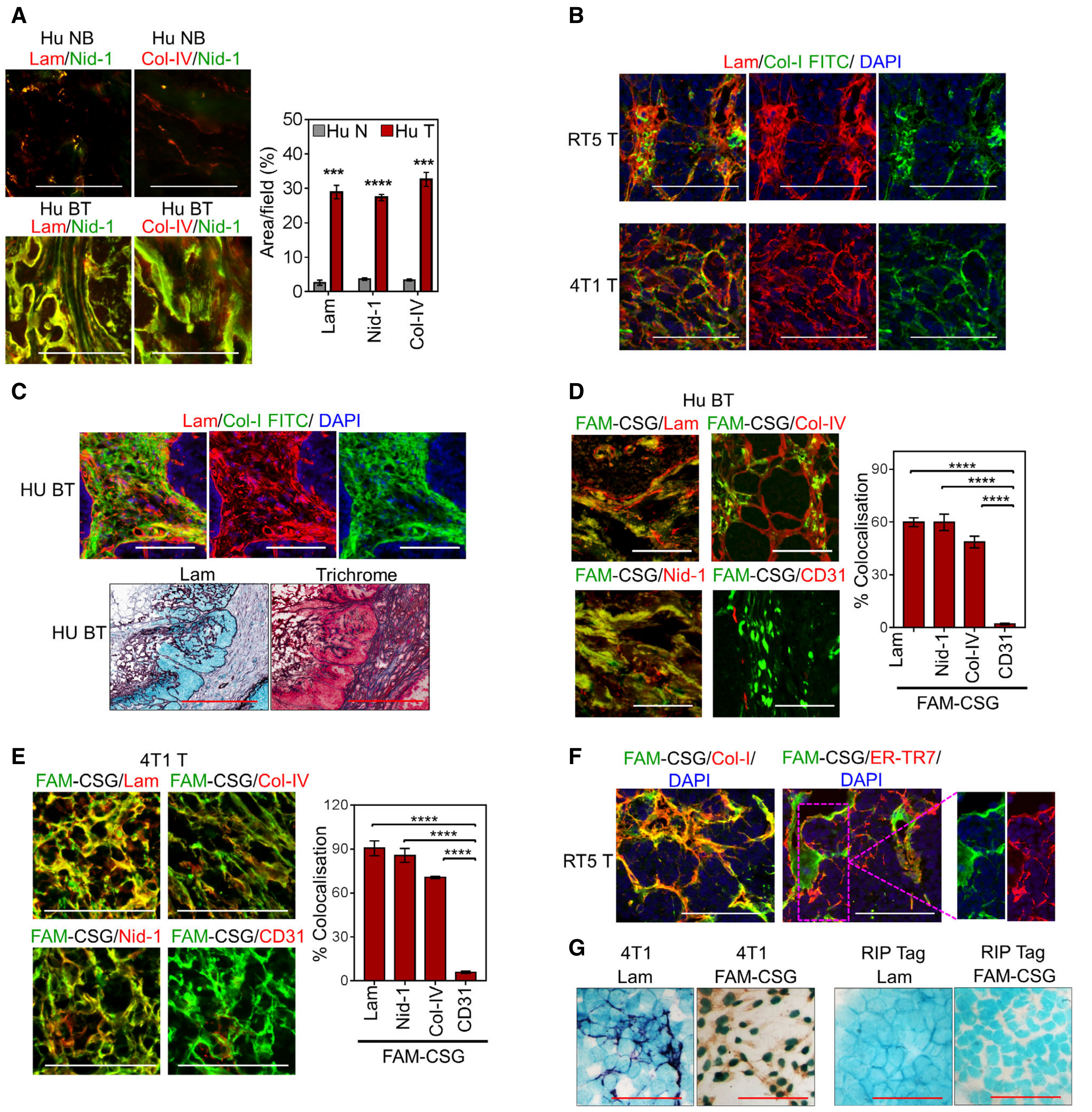

Figure EV2. CSG binding co-localises with high abundant tumour ECM.

A Normal human breast (Hu NB) and human breast carcinoma (Hu BT) were stained for the indicated ECM components. Representative micrographs are shown (left) and corresponding bar graphs (right) quantifying the area positive for each ECM marker (mean ± SEM; $n = 3$, ***$P < 0.001$ and ***$P < 0.0001$ by multiple t-tests). Scale bars: 50 μm.

B Co-staining analysis of 4T1 (4T1 T) and RIP1-Tag5 (RT5 T) tumours for laminin (lam, red) and collagen I (Col-I-FITC, green) expression. Scale bars: 50 μm.

C Top panel: Similar analysis as in panel (B) for Hu BT tumour. Lower panel: Serial tissue sections of Hu BT comparing immunostaining of laminin and trichrome. Scale bars: 300 μm.

D, E Co-staining of FAM-CSG (green) and ECM markers or CD31 (red) on Hu BT (D) and 4T1 tumour (E). Representative micrographs are shown. Scale bars: 50 μm. Corresponding bar graphs (right) show co-localisation of indicated markers with CSG (mean ± SEM; $n = 4$, ****$P < 0.0001$ by one-way ANOVA test with Tukey's correction).

F Co-staining of FAM-CSG (green) and collagen I and ER-TR7 (red) in RT5 T. Scale bars: 50 μm.

G Analysis of laminin expression (lam, purple), FAM-CSG binding detected with anti-FITC antibody (brown) and nuclear staining (methyl green) in cultured 4T1 and malignant pancreatic beta cells derived from RIP-Tag mice. Scale bars: 25 μm.

 © 2019 The Authors

Figure EV3. The effects of TNFα-CSG on immune infiltrates in tumours.

A, B Co-staining of RIP1-Tag5 tumour sections treated with 5 daily doses of indicated compounds. Micrographs show $CD8^+$ T-cell (red) infiltration relative to collagen IV (A; green) and $CD31^+$ blood vessels (B; green). Scale bars: 100 μm.

C Left panels show representative flow cytometry plots of quantification of immune cells in 4T1 tumours treated with 5 daily i.v. injections of 0.5 μg of unconjugated TNFα or TNFα-CSG, or left untreated (UT). Bar charts show mean ± SEM of cell counts in each treatment group (data are shown for one of the two repeated experiments; $n = 4$; *$P < 0.05$, **$P < 0.005$ and ***$P < 0.001$, non-parametric tests).

D Co-staining of ALB-Tag HCC tumour sections treated with 5 daily doses of indicated compounds. Micrographs show $CD8^+$ and $CD4^+$ T-cell (green) infiltration. Scale bars: 100 μm. Bar charts show mean ± SEM of cell counts in each treatment group ($n = 4$; ***$P < 0.001$ and ****$P < 0.0001$ by one-way ANOVA test with Tukey's correction).

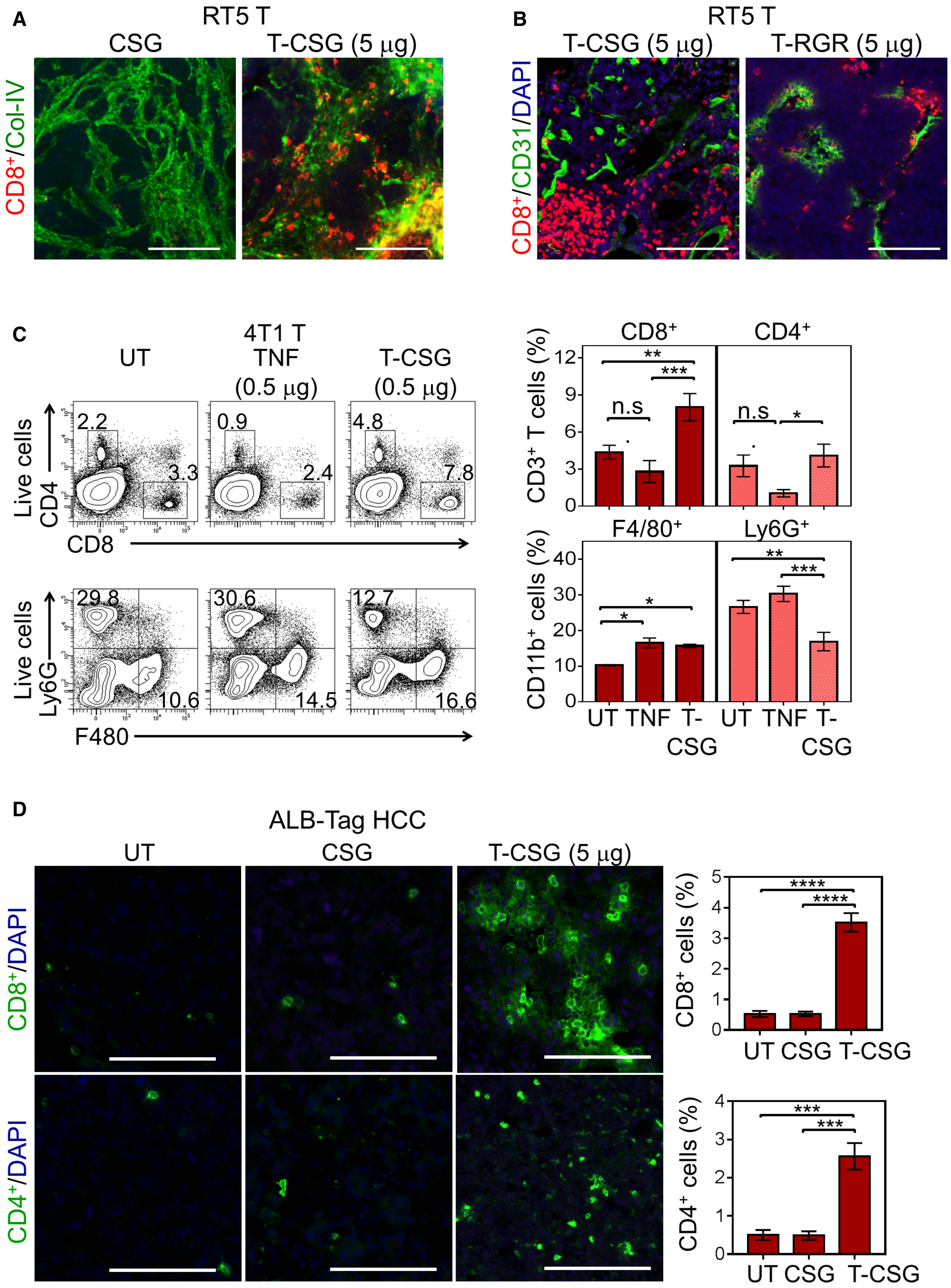

Figure EV3.

 © 2019 The Authors

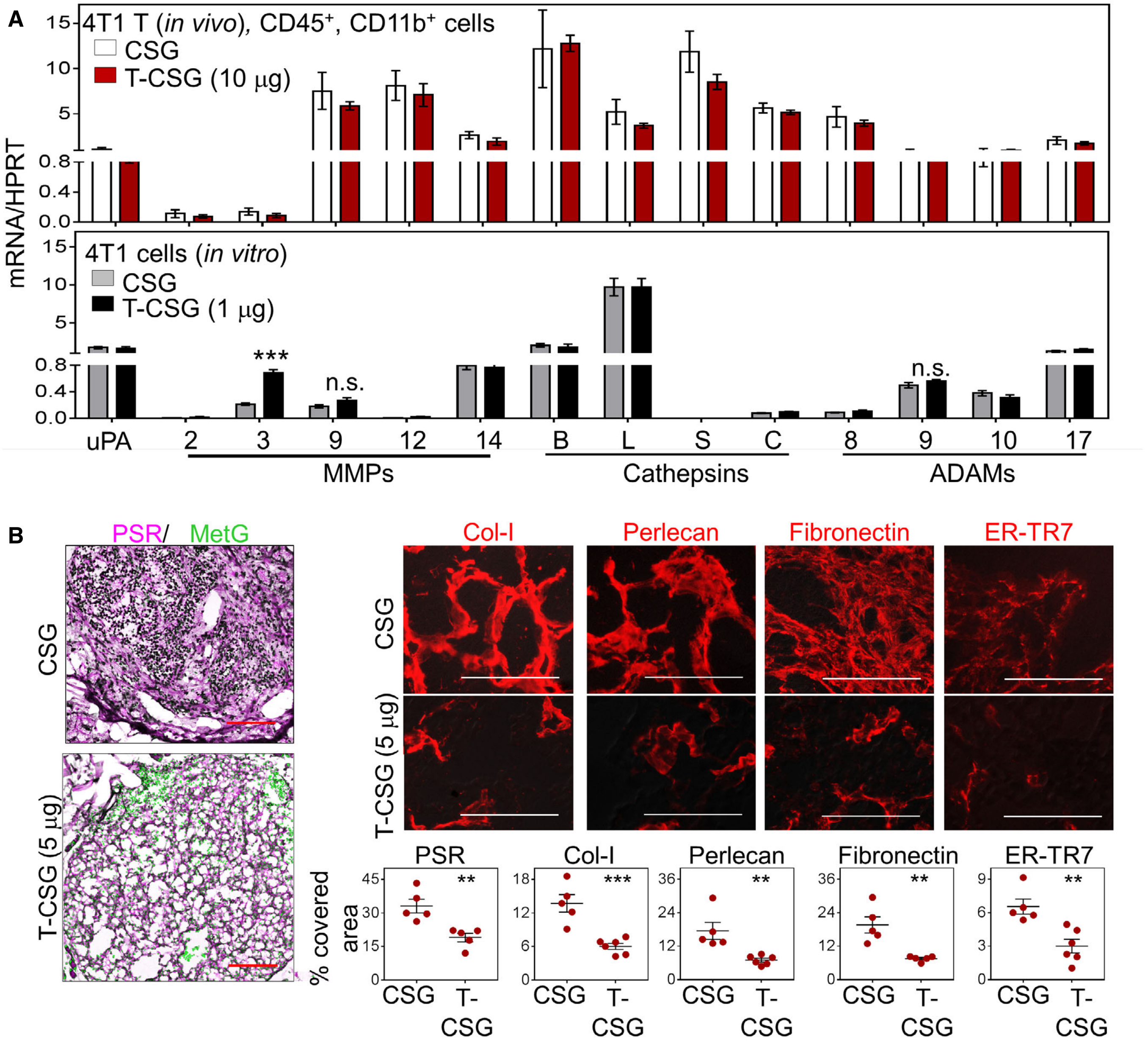

Figure EV4. The effects of TNFα-CSG on immune infiltrates and protease mRNA transcripts and ECM components.

A Quantitative PCR analysis was performed on (top) macrophages ($CD45^+/CD11b^+$ cells) isolated from 4T1 tumours treated with 5 daily injections of indicated compounds and (bottom) cultured 4T1 cells incubated overnight with indicated compounds. Hypoxanthine-guanine phosphoribosyltransferase (HPRT) was used as a standard (mean ± SEM are shown; $n = 5$; ***$P < 0.001$ by Student's *t*-tests).

B Co-staining of tumour sections from RIP1-Tag5 mice treated with indicated compounds for picrosirius red (PSR; counterstained with methyl green for nuclei staining), collagen I (Col-I), heparan sulphate proteoglycan 2 (perlecan), fibronectin and ER-TR7. Top: Micrographs are shown for an individual staining (red). Bottom: Percentage of expression for each tumour and as means ± SEM ($n = 3$ mice/group, **$P < 0.01$ and ***$P < 0.001$, Student's *t*-test) are shown. Scale bars: 50 μm.

© 2019 The Authors

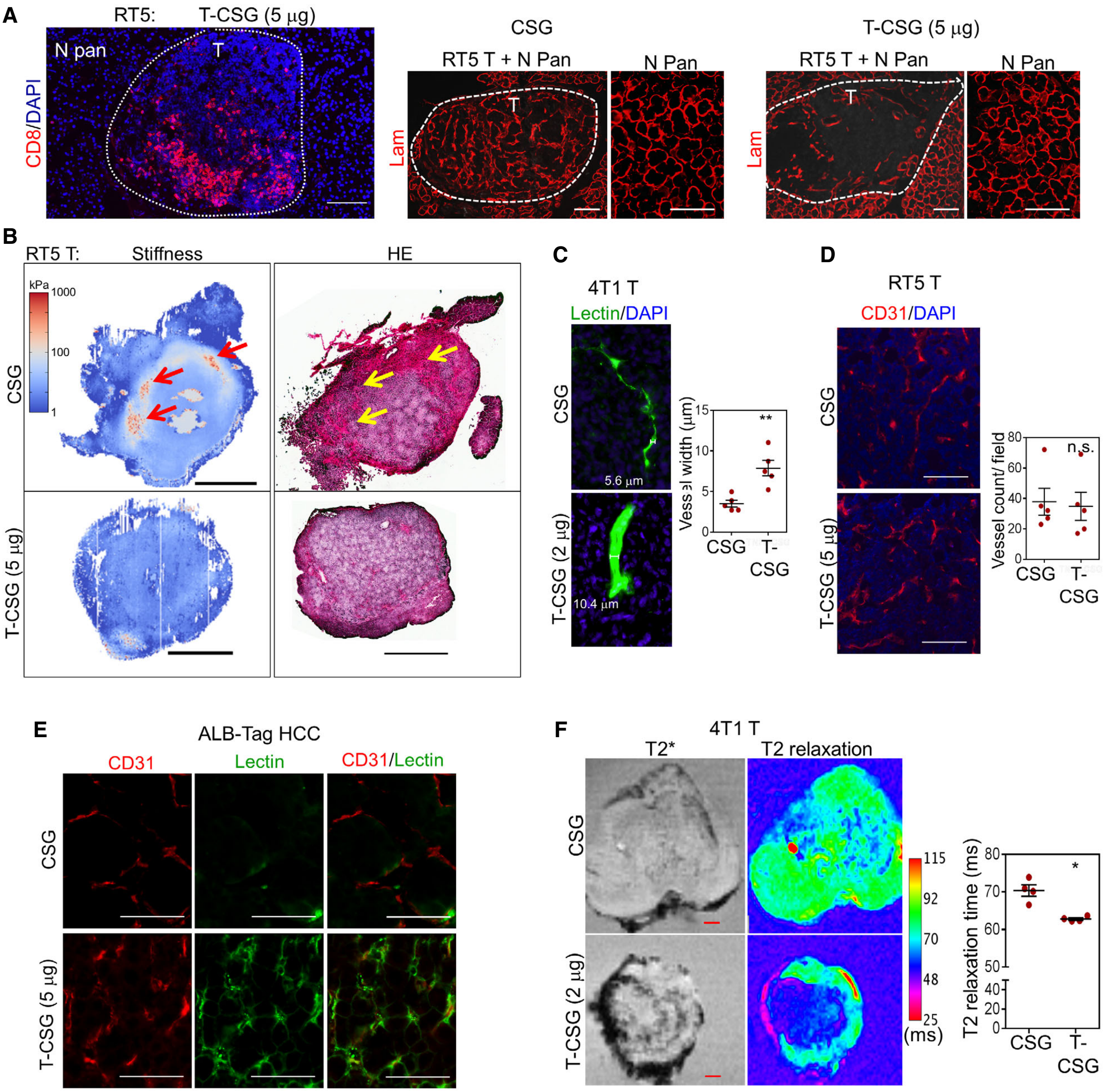

Figure EV5. The effects of TNFα-CSG on tumour ECM and perfusion.

A Comparison of CD8+ T-cell (red) or ECM content (laminin, red) in tumour (T) and the surrounding normal pancreas (N Pan) for RIP1-Tag5 mice treated with 5 daily doses on indicated compound. Scale bars: 100 µm.

B Stiffness analyses of the RIP1-Tag5 tumours following treatment with 4 × daily i.v. injections of indicated compounds. Tumour stiffness was analysed on day 5. Left panel: En face quantitative micro-elastograms showing tumour stiffness (red arrow: stiffest zones). Right panel: The corresponding micrograph of haematoxylin and eosin (HE) staining of the tumours (yellow arrow: ECM-rich zone). Scale bars: 400 µm.

C Micrographs show lectin+ vessels (green) in 4T1 tumours, after mice were treated with 5 daily injections of indicated compounds and perfused with fluorescein-labelled lectin to visualise patent blood vessels. Data show average vessel width for individual tumours and mean ± SEM for each group ($n = 4$; $^{**}P < 0.005$ by Student's *t*-test).

D Micrographs show CD31+ vessels (red) in RIP1-Tag5 tumours, after mice were treated with 5 daily injections of indicated compounds. Scale bars: 100 µm. Data show mean vessel counts per tumour and mean ± SEM for each group ($n = 5$; $P = 0.8202$ by Student's *t*-test).

E Co-staining of lectin-perfused ALB-Tag HCC tumours (treated as in panel A) with CD31 (red, blood vessels). Scale bars: 100 µm.

F Left: *Ex vivo* MRI scans of T2* (dark contrast) and T2 relaxation (magenta) show the presence of IONP in 4T1 tumours treated as in (C) followed by an i.v. injection of 5 mg/kg IONP. IONP uptake in tumours was imaged 4 h after IONP injection. Scale bars: 1 mm. Right: Reduction in T2 relaxation time indicates the relative amount of IONP in individual tumours and mean ± SEM ($n = 4$; $^{*}P < 0.05$ by Student's *t*-test).

 © 2019 The Authors
