# Supplementary material for: Immune‐mediated ECM depletion improves tumour perfusion and payload delivery
Source: EMBO Mol Med. 2019 Nov 11;11(12):e10923. doi: 10.15252/emmm.201910923 (PMC6895610; doi:10.15252/emmm.201910923)
Supplement: Supplementary file 4 — Source Data for Figure 1 [file EMMM-11-e10923-s003.pdf]

Figure 1B: FAM-CSG homing detected with anti-FITC HRP

RIP1-Tag5 tumour

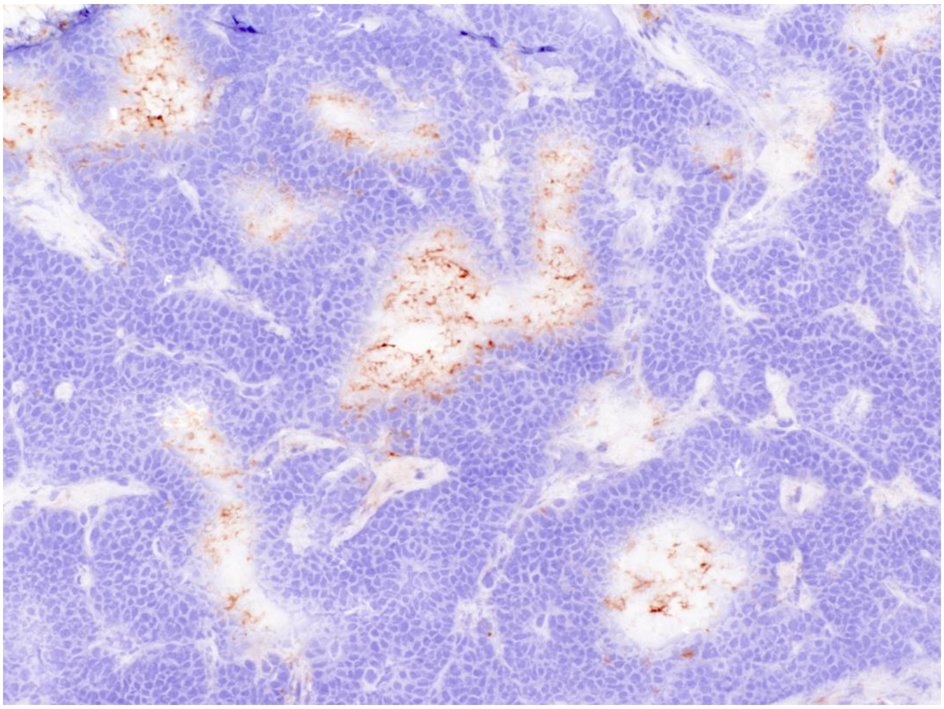

4T1 tumour

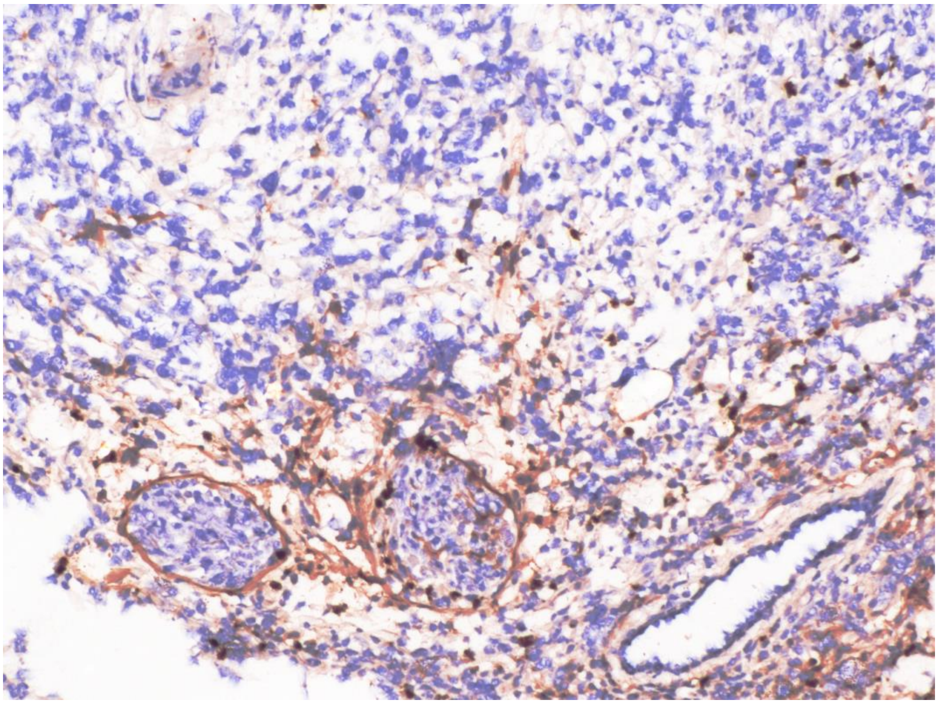

Figure 1B: FAM-CSG homing detected with anti-FITC HRP

Kidney

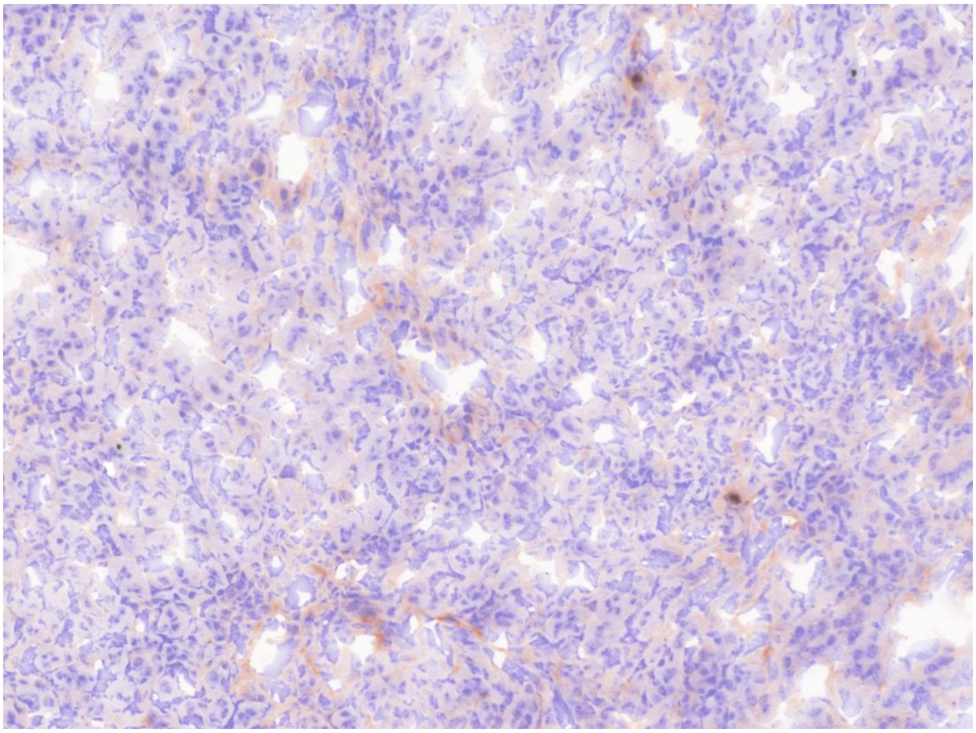

Intestine

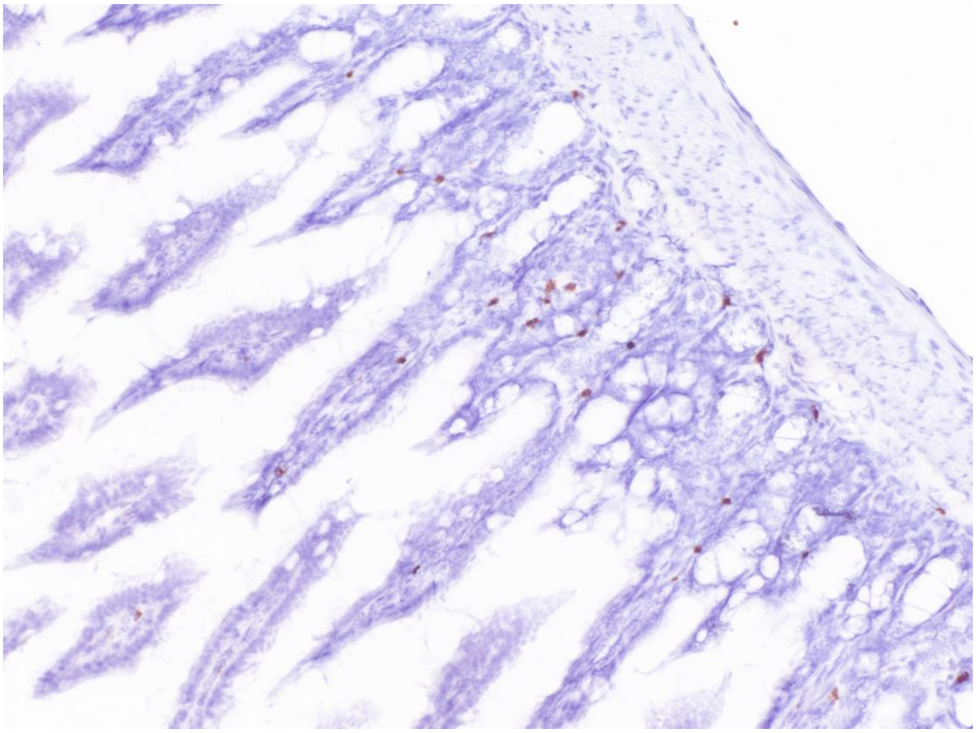

Figure 1B: FAM-CSG homing detected with anti-FITC HRP

Liver

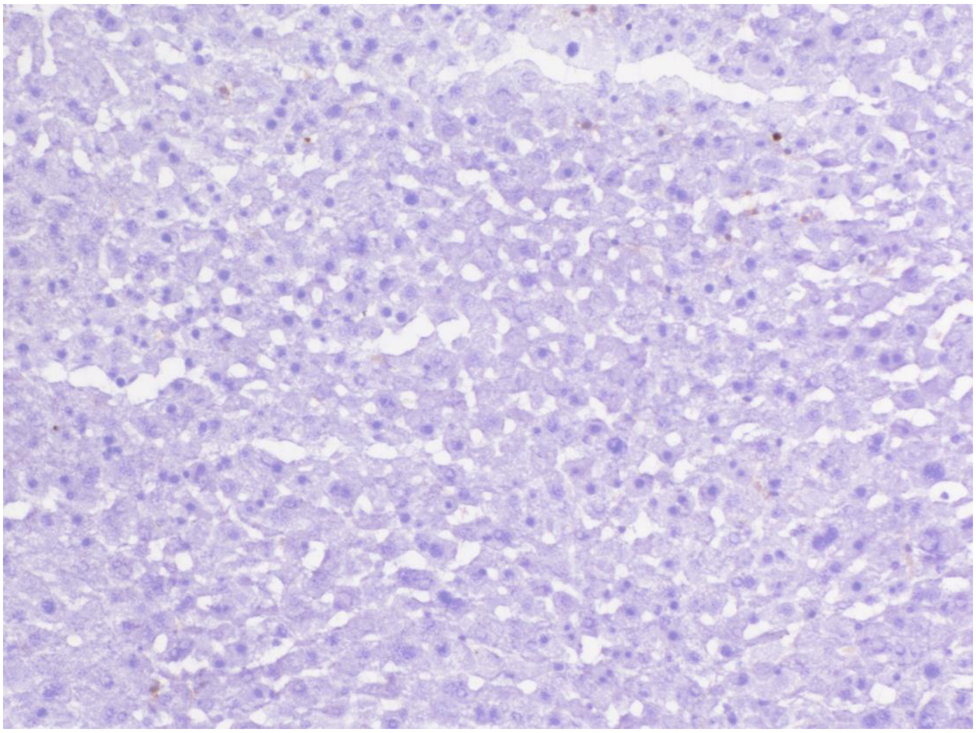

Muscle

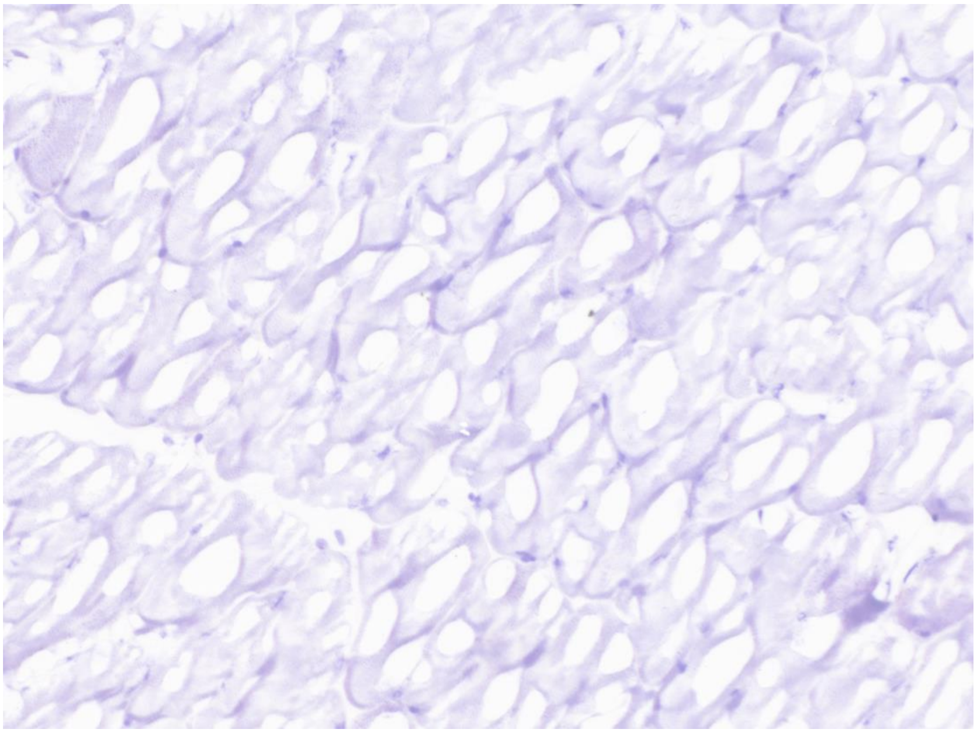

Figure 1B: FAM-CSG homing detected with anti-FITC HRP

Spleen

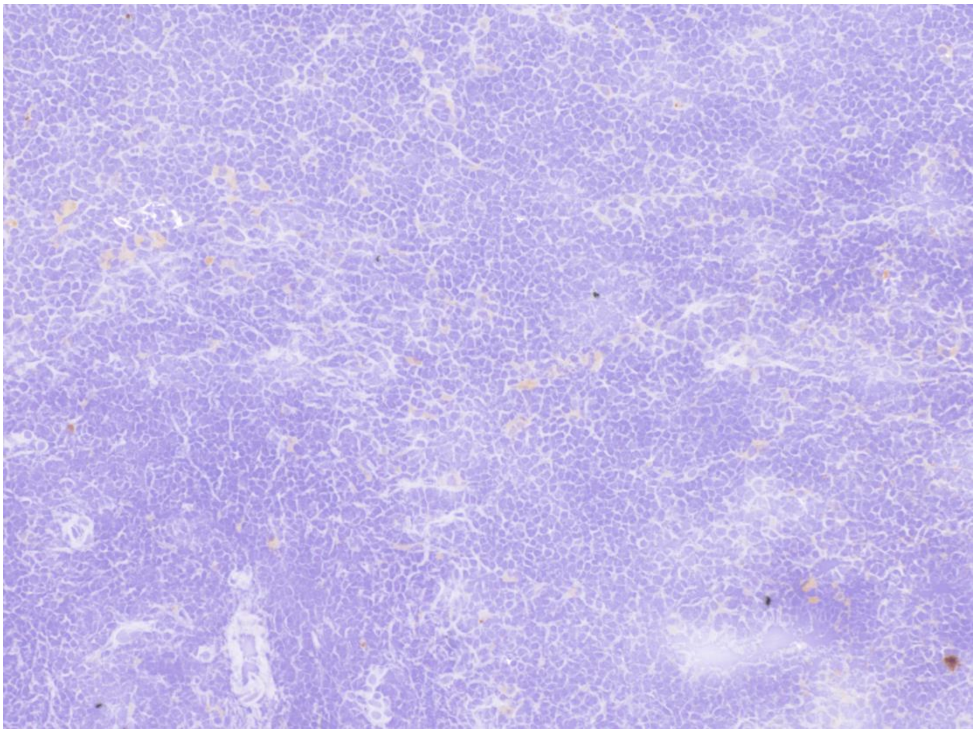

Heart

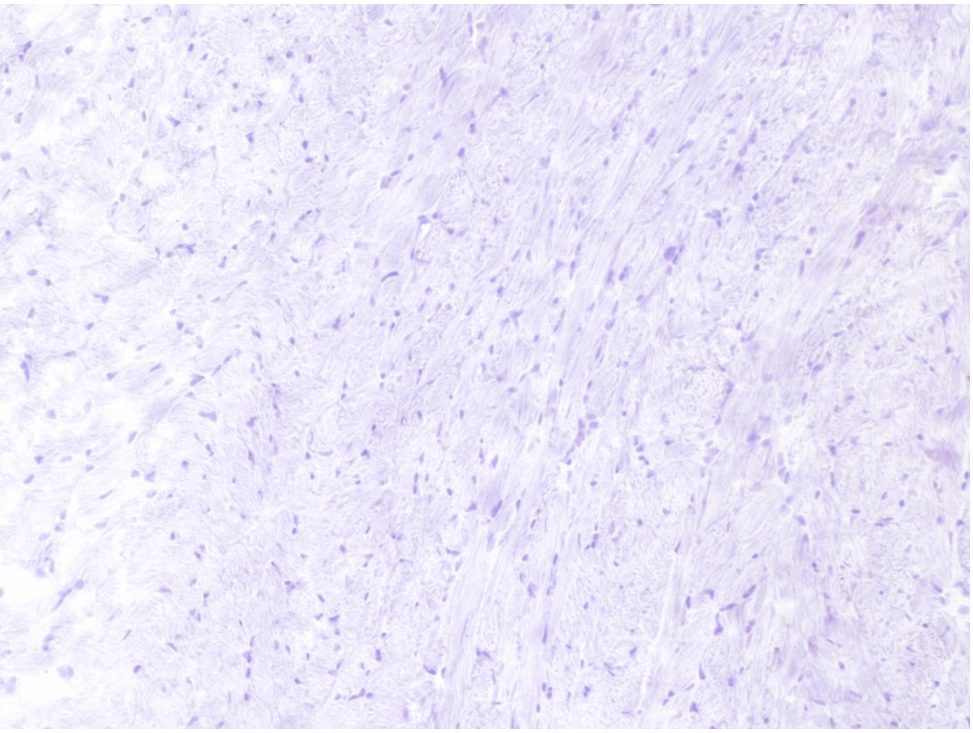

Figure 1B: FAM-CSG homing detected with anti-FITC HRP

Pancreas

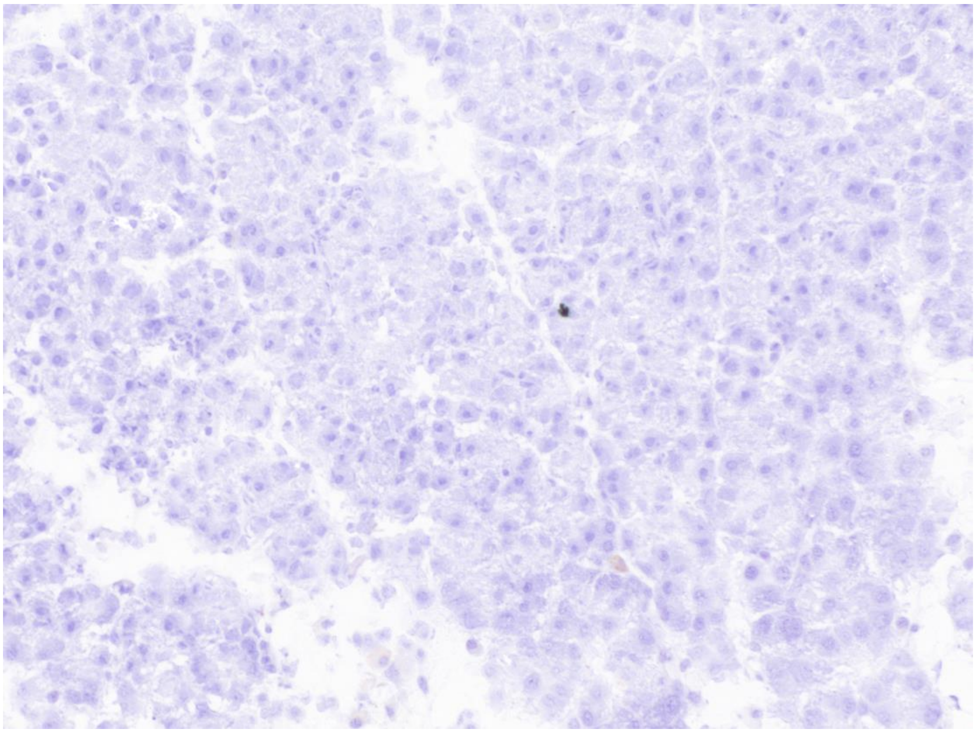

Brain

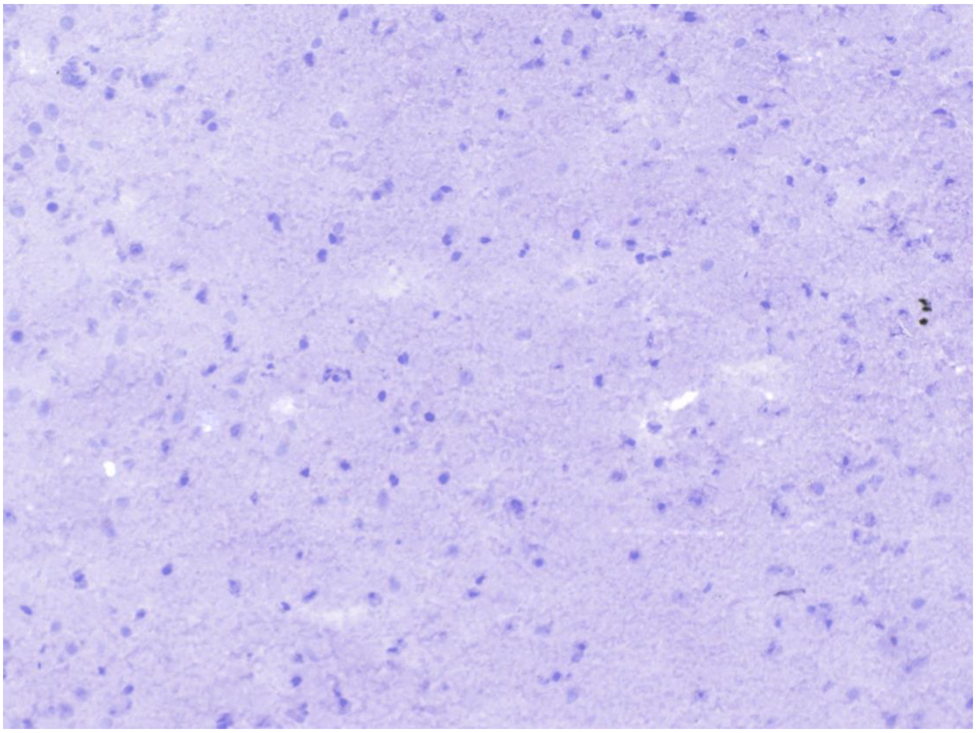

Figure 1B: FAM-CSG homing detected with anti-FITC HRP

Skin

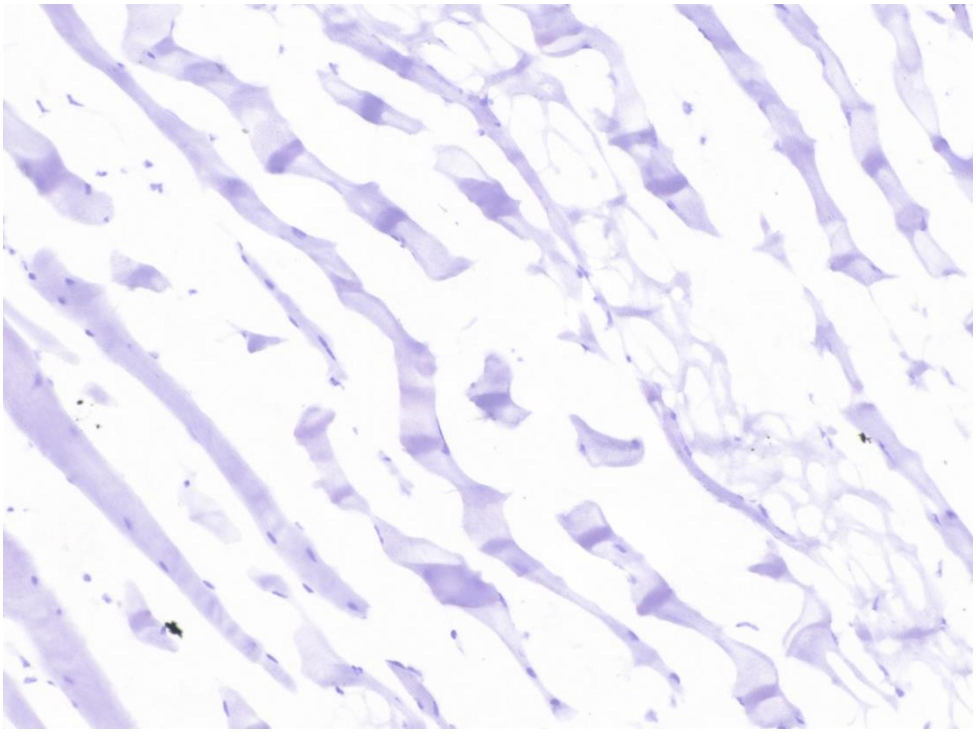

Lymph node

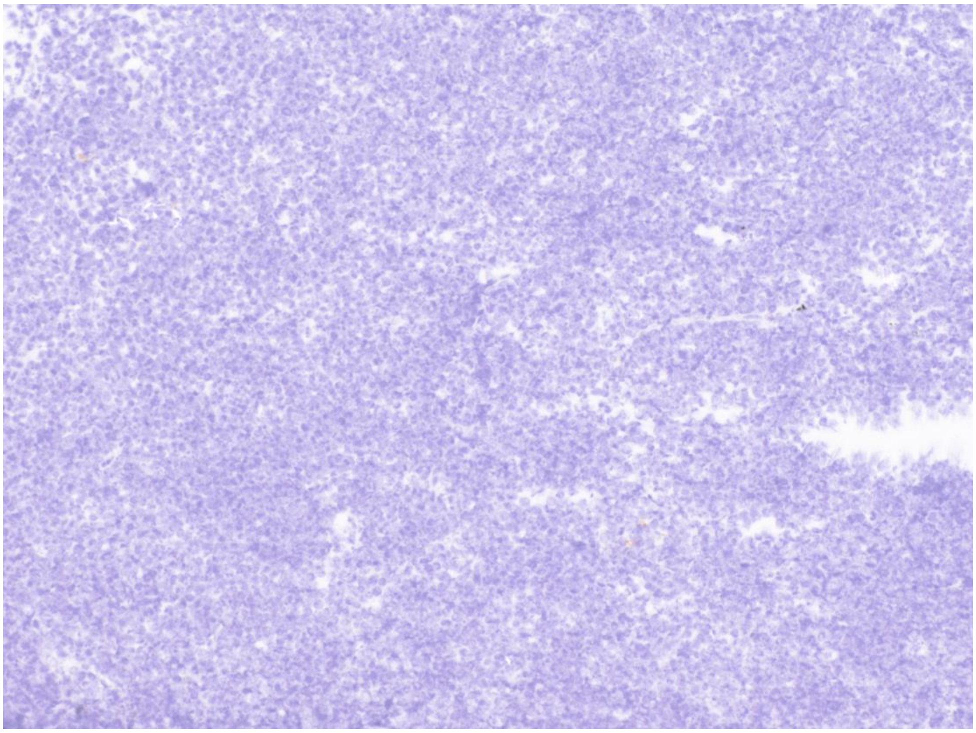

Figure 1D: FAM-CSG homing detected with anti-FITC HRP

Human: normal breast FAM-CSG

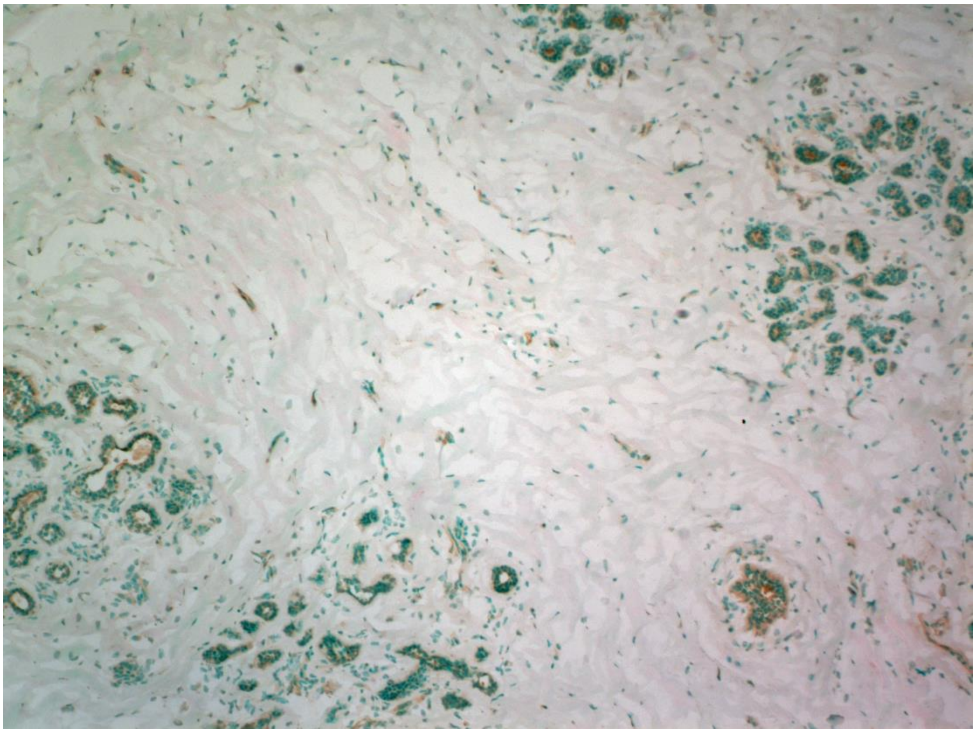

Human: breast tumour FAM-CSG

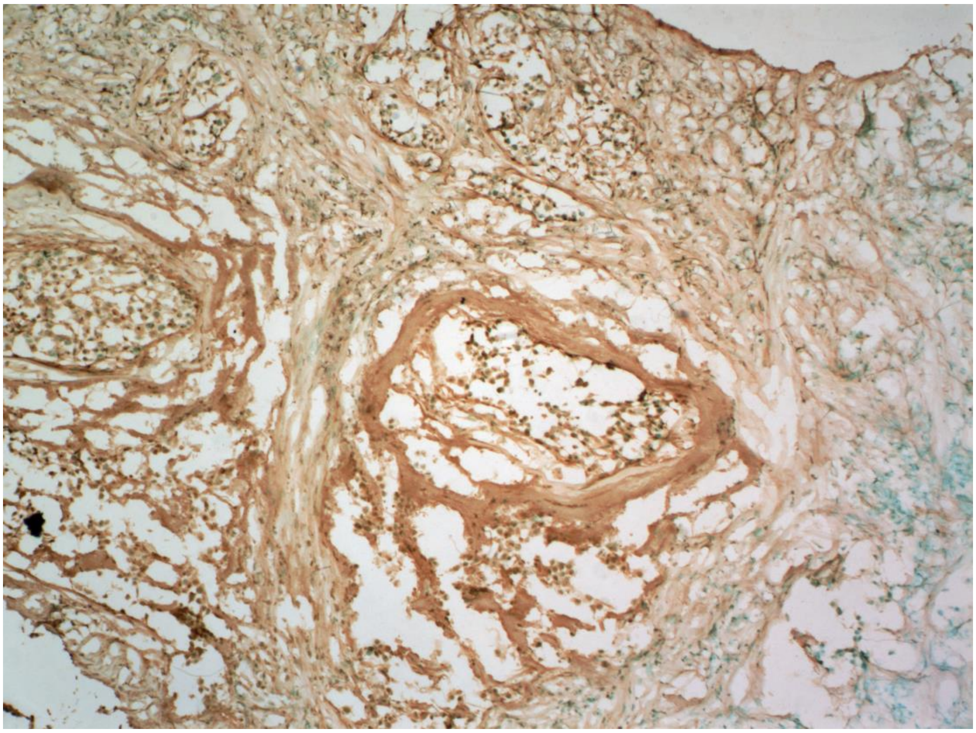

Figure 1D: FAM-CSG homing detected with anti-FITC HRP

Human: breast tumour CSG (excess) + FAM-CSG

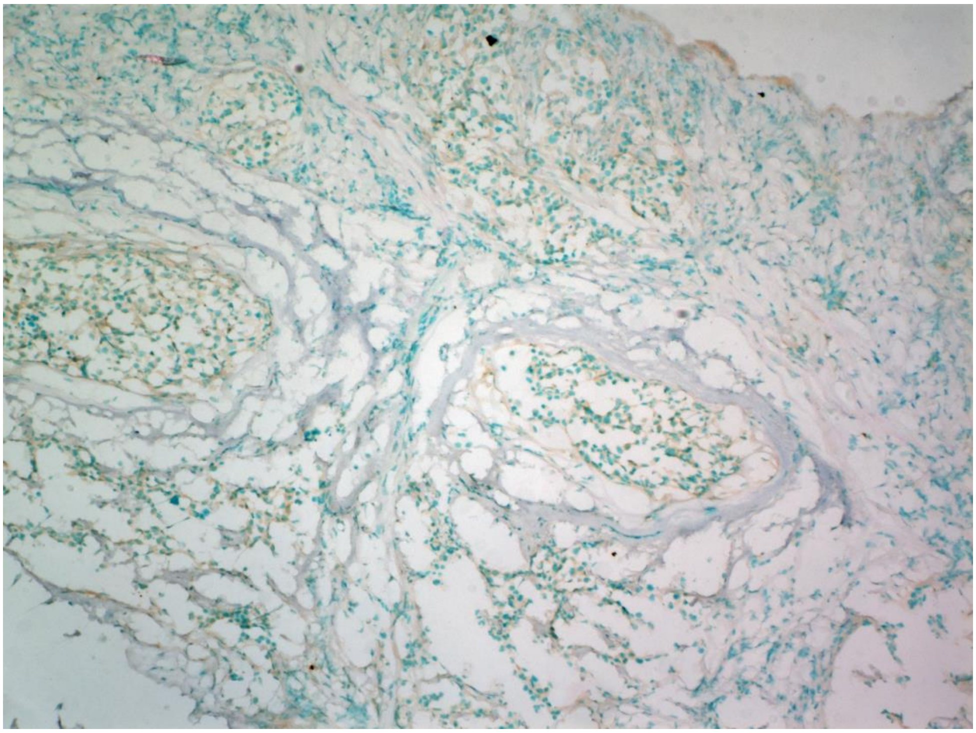

Human: breast tumour FAM-ARA

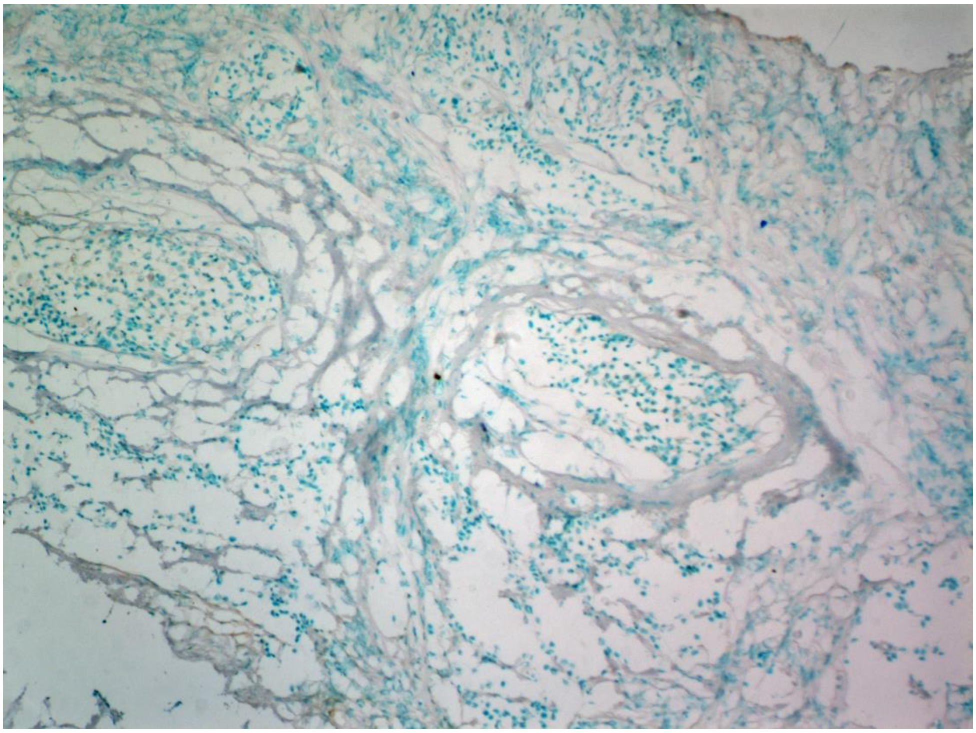

Figure 1 C **% anti-FITC/field**

**4T1 Tumour**	**RIP1-Tag5 Tumour**	**Liver**	**Intestine**	**Muscle**	**Spleen**	**Heart**	**Pancreas**	**Skin**	**Lymph nodes**	**Brain**
22.34	9.8	0.66	0.65	0.05	0.19	0.12	0	0	0	0
17.74	9.78	1.57	1.2	0	0.02	0	0	0	0	0
13.39	28.93	0.33	0.77	1.4	0.09	0	0	0	0	0
11.34	4.54									
	9.43									
	7.33									
	9.79									
	15.08									

Analysis 1 way ANOVA (Correct for mulple comparisons using Tukey test

Number of families	1
Number of comparisons	55
Alpha	0.05

**Tukey's multiple compa**	**Mean Diff.**	**95.00% CI of diff.**	**Significant**	**Summary**	**Adjusted P Value**	
4T1 vs. RIP-Tag	4.368	-4.389 to 13.12	No	ns	0.801	
4T1 vs. Liver	15.35	4.428 to 26.27	Yes	**	0.0015	
4T1 vs. Intestine	15.33	4.408 to 26.25	Yes	**	0.0015	
4T1 vs. Muscle	15.72	4.798 to 26.64	Yes	**	0.0011	
4T1 vs. Spleen	16.1	5.181 to 27.02	Yes	***	0.0008	
4T1 vs. Heart	16.16	5.241 to 27.08	Yes	***	0.0007	
4T1 vs. Pancreas	16.2	5.281 to 27.12	Yes	***	0.0007	
4T1 vs. Skin	16.2	5.281 to 27.12	Yes	***	0.0007	
4T1 vs. LN	16.2	5.281 to 27.12	Yes	***	0.0007	
4T1 vs. Brain	16.2	5.281 to 27.12	Yes	***	0.0007	
RIP-Tag vs. Liver	10.98	1.301 to 20.66	Yes	*	0.0165	
RIP-Tag vs. Intestine	10.96	1.281 to 20.64	Yes	*	0.0168	
RIP-Tag vs. Muscle	11.35	1.671 to 21.03	Yes	*	0.0119	
RIP-Tag vs. Spleen	11.74	2.054 to 21.42	Yes	**	0.0084	
RIP-Tag vs. Heart	11.8	2.114 to 21.48	Yes	**	0.008	
RIP-Tag vs. Pancreas	11.84	2.154 to 21.52	Yes	**	0.0077	
RIP-Tag vs. Skin	11.84	2.154 to 21.52	Yes	**	0.0077	
RIP-Tag vs. LN	11.84	2.154 to 21.52	Yes	**	0.0077	
RIP-Tag vs. Brain	11.84	2.154 to 21.52	Yes	**	0.0077	
Liver vs. Intestine	-0.02	-11.7 to 11.66	No	ns	>0.9999	
Liver vs. Muscle	0.37	-11.31 to 12.05	No	ns	>0.9999	
Liver vs. Spleen	0.7533	-10.92 to 12.43	No	ns	>0.9999	
Liver vs. Heart	0.8133	-10.86 to 12.49	No	ns	>0.9999	
Liver vs. Pancreas	0.8533	-10.82 to 12.53	No	ns	>0.9999	
Liver vs. Skin	0.8533	-10.82 to 12.53	No	ns	>0.9999	
Liver vs. LN	0.8533	-10.82 to 12.53	No	ns	>0.9999	
Liver vs. Brain	0.8533	-10.82 to 12.53	No	ns	>0.9999	
Intestine vs. Muscle	0.39	-11.29 to 12.07	No	ns	>0.9999	
Intestine vs. Spleen	0.7733	-10.9 to 12.45	No	ns	>0.9999	
Intestine vs. Heart	0.8333	-10.84 to 12.51	No	ns	>0.9999	
Intestine vs. Pancreas	0.8733	-10.8 to 12.55	No	ns	>0.9999	
Intestine vs. Skin	0.8733	-10.8 to 12.55	No	ns	>0.9999	
Intestine vs. LN	0.8733	-10.8 to 12.55	No	ns	>0.9999	
Intestine vs. Brain	0.8733	-10.8 to 12.55	No	ns	>0.9999	
Muscle vs. Spleen	0.3833	-11.29 to 12.06	No	ns	>0.9999	
Muscle vs. Heart	0.4433	-11.23 to 12.12	No	ns	>0.9999	
Muscle vs. Pancreas	0.4833	-11.19 to 12.16	No	ns	>0.9999	
Muscle vs. Skin	0.4833	-11.19 to 12.16	No	ns	>0.9999	
Muscle vs. LN	0.4833	-11.19 to 12.16	No	ns	>0.9999	
Muscle vs. Brain	0.4833	-11.19 to 12.16	No	ns	>0.9999	
Spleen vs. Heart	0.06	-11.62 to 11.74	No	ns	>0.9999	
Spleen vs. Pancreas	0.1	-11.58 to 11.78	No	ns	>0.9999	
Spleen vs. Skin	0.1	-11.58 to 11.78	No	ns	>0.9999	
Spleen vs. LN	0.1	-11.58 to 11.78	No	ns	>0.9999	
Spleen vs. Brain	0.1	-11.58 to 11.78	No	ns	>0.9999	
Heart vs. Pancreas	0.04	-11.64 to 11.72	No	ns	>0.9999	
Heart vs. Skin	0.04	-11.64 to 11.72	No	ns	>0.9999	
Heart vs. LN	0.04	-11.64 to 11.72	No	ns	>0.9999	
Heart vs. Brain	0.04	-11.64 to 11.72	No	ns	>0.9999	
Pancreas vs. Skin	0	-11.68 to 11.68	No	ns	>0.9999	
Pancreas vs. LN	0	-11.68 to 11.68	No	ns	>0.9999	
Pancreas vs. Brain	0	-11.68 to 11.68	No	ns	>0.9999	
Skin vs. LN	0	-11.68 to 11.68	No	ns	>0.9999	
Skin vs. Brain	0	-11.68 to 11.68	No	ns	>0.9999	
LN vs. Brain	0	-11.68 to 11.68	No	ns	>0.9999	

Figure 1 E **% anti-FITC/field**

FAM-CSG on Human Normal Breast	FAM-CSG on Human Breast Tumour	CSG (excess) + FAM-CSG on Human Breast tumour	FAM-ARA on Human Breast Tumour
5.3	29.1	0.1	0.6
2.8	13.1	7.1	2.9
6.1	34.2	0.1	1.4
	40.5	0.1	0.1
	32.2	0.4	0.1
	41.4	0.6	0.3
	10.7		0.4

Analysis 1 way ANOVA (Correct for mulple comparisons using Tukey test)

Number of families	1
Number of comparisons per family	6
Alpha	0.05

Tukey's multiple comparisons test	Mean Diff.	95.00% CI of diff.	Significant?	Summary	Adjusted P Value	
Normal Breast FAM-CSG vs. Tumor FAM-CSG	-24.01	-37.82 to -10.2	Yes	***	0.0005	
Normal Breast FAM-CSG vs. Tumor CSG + FAM-CSG	3.333	-10.81 to 17.48	No	ns	0.9098	
Normal Breast FAM-CSG vs. Tumor FAM-ARA	3.905	-9.901 to 17.71	No	ns	0.8557	
Tumor FAM-CSG vs. Tumor CSG + FAM-CSG	27.34	16.21 to 38.47	Yes	****	<0.0001	
Tumor FAM-CSG vs. Tumor FAM-ARA	27.91	17.22 to 38.61	Yes	****	<0.0001	
Tumor CSG + FAM-CSG vs. Tumor FAM-ARA	0.5714	-10.56 to 11.7	No	ns	0.9989	
