# Supplementary material for: Immune‐mediated ECM depletion improves tumour perfusion and payload delivery
Source: EMBO Mol Med. 2019 Nov 11;11(12):e10923. doi: 10.15252/emmm.201910923 (PMC6895610; doi:10.15252/emmm.201910923)
Supplement: Supplementary file 5 — Source Data for Figure 2 [file EMMM-11-e10923-s004.pdf]

Figure 2 B: Co-staining analysis

C3H Pancreas: collagen IV/Nidogen I/DAPI

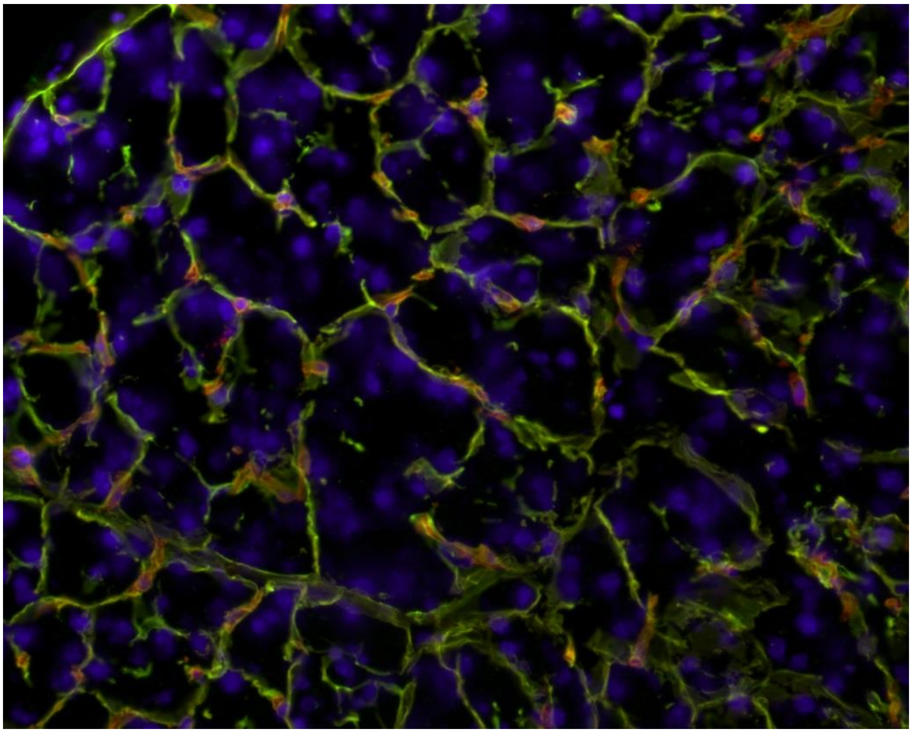

C3H Pancreas: Laminin/Nidogen I/DAPI

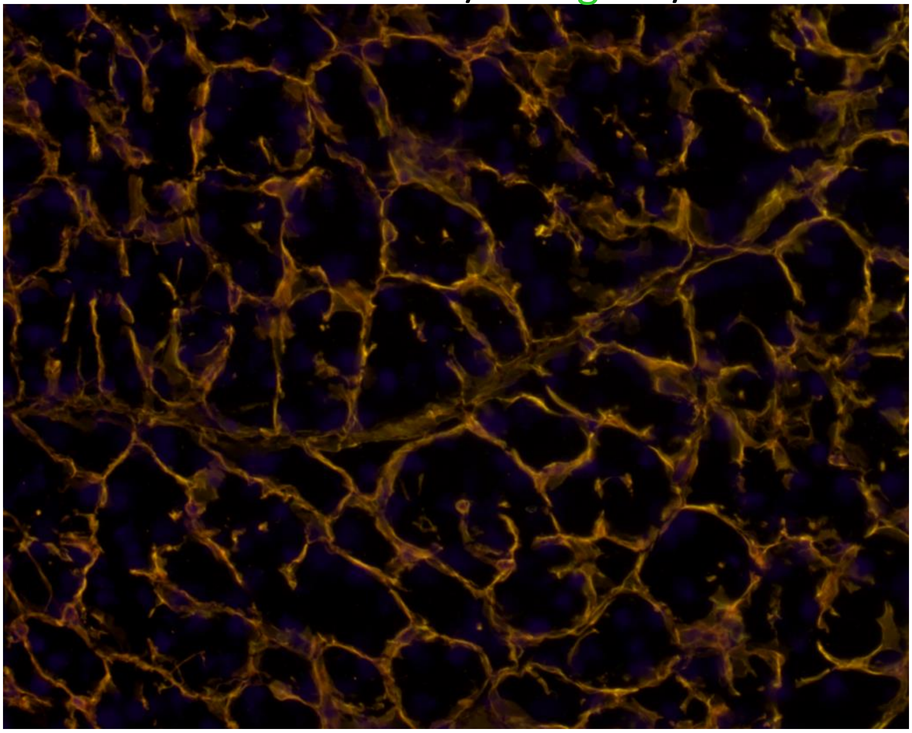

Figure 2 B: Co-staining analysis

RIP1-Tag5 tumour: collagen IV/Nidogen I/DAPI

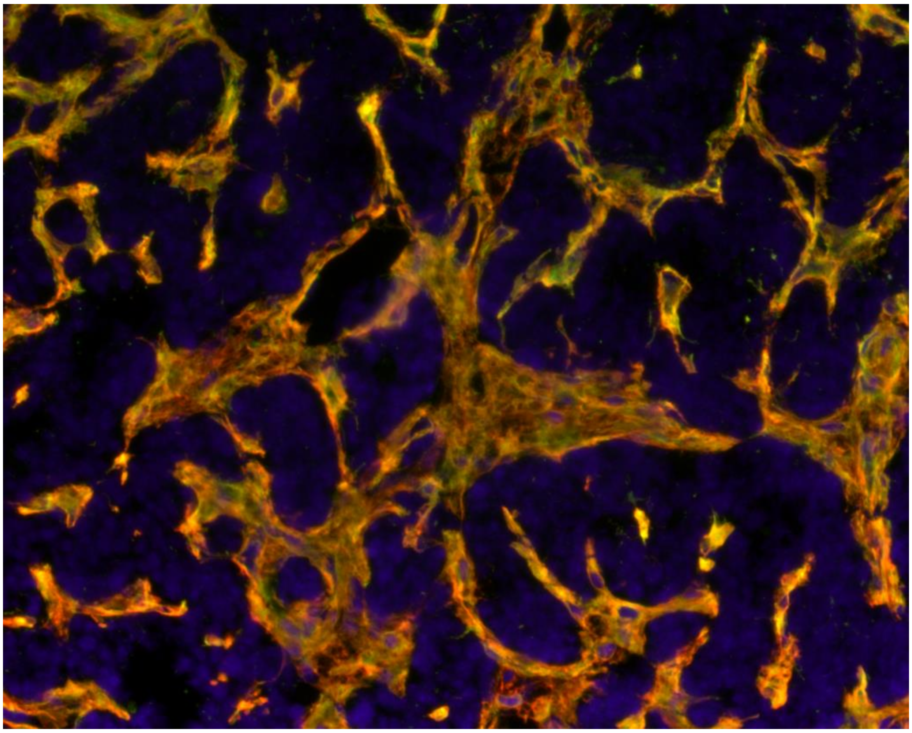

RIP1-Tag5 tumour: Laminin/Nidogen I/DAPI

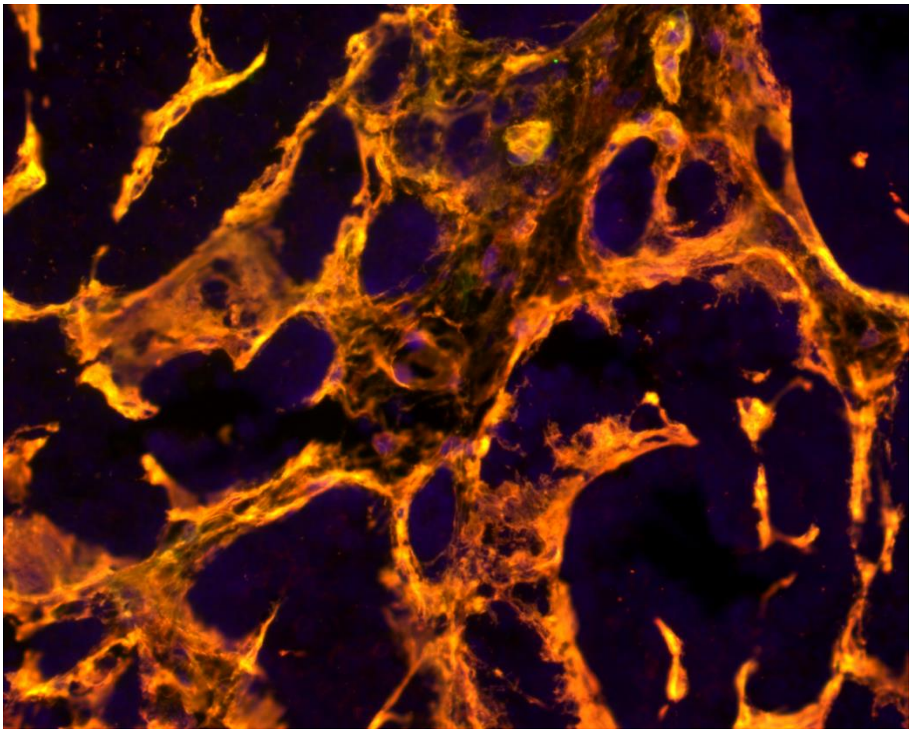

Figure 2 C: Co-staining analysis

C3H Panreas: CD31/Laminin/DAPI

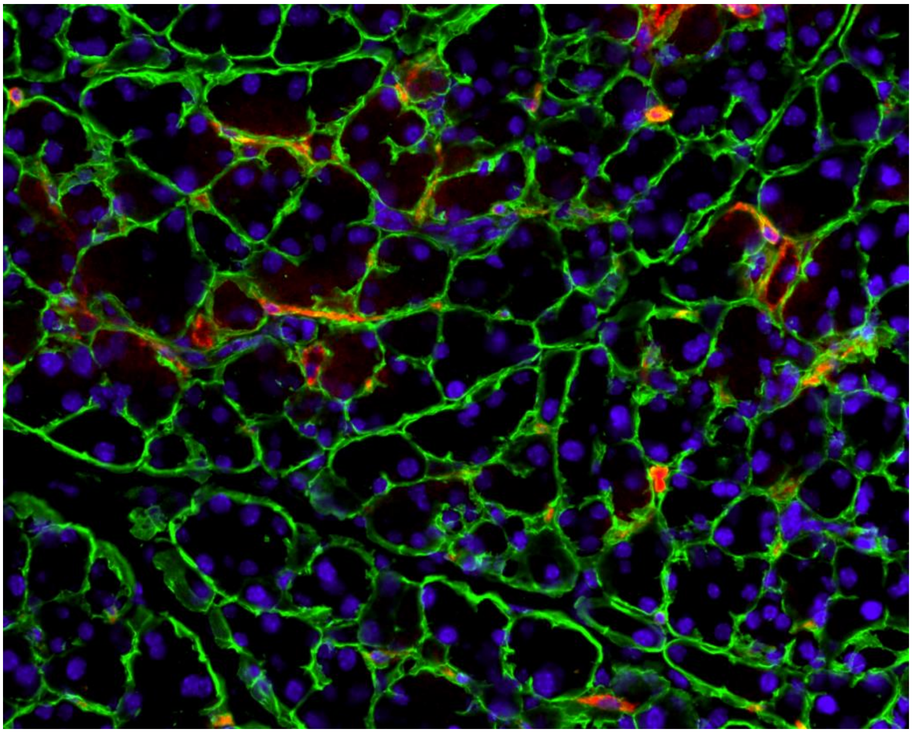

RIP1-Tag5 tumour: CD31/Laminin/DAPI

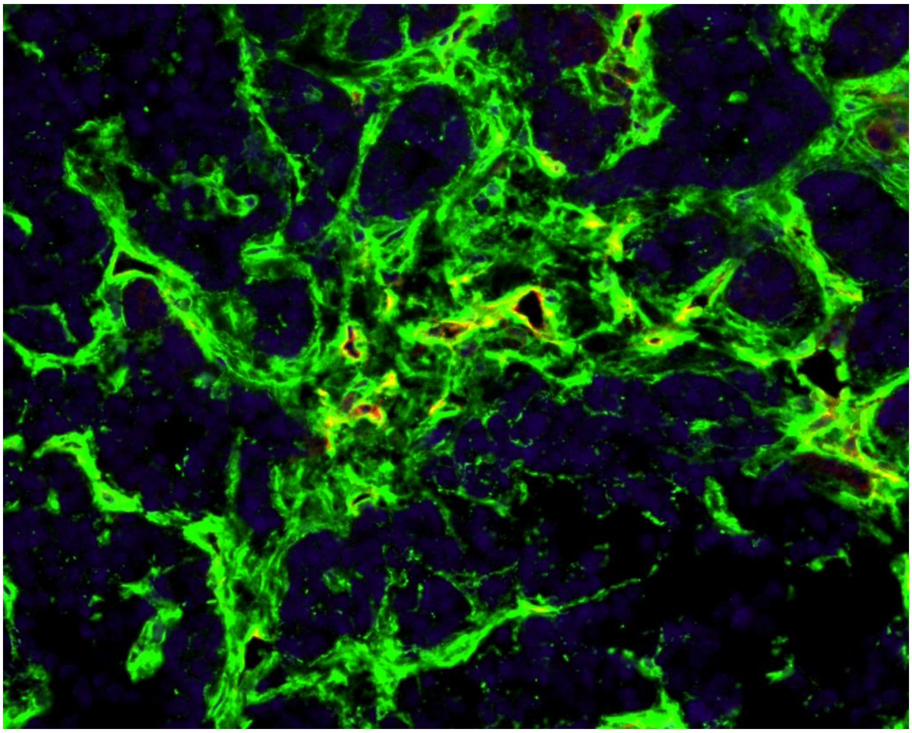

Figure 2 D: Co-staining analysis

RIP1-Tag5 tumour: FAM-CSG/Laminin/DAPI

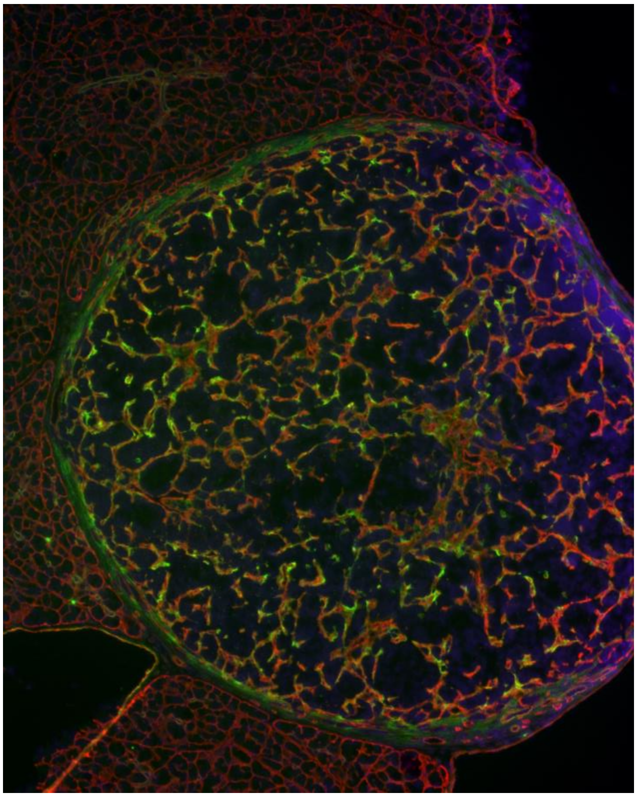

RIP1-Tag5 tumour: Laminin/CD31/DAPI

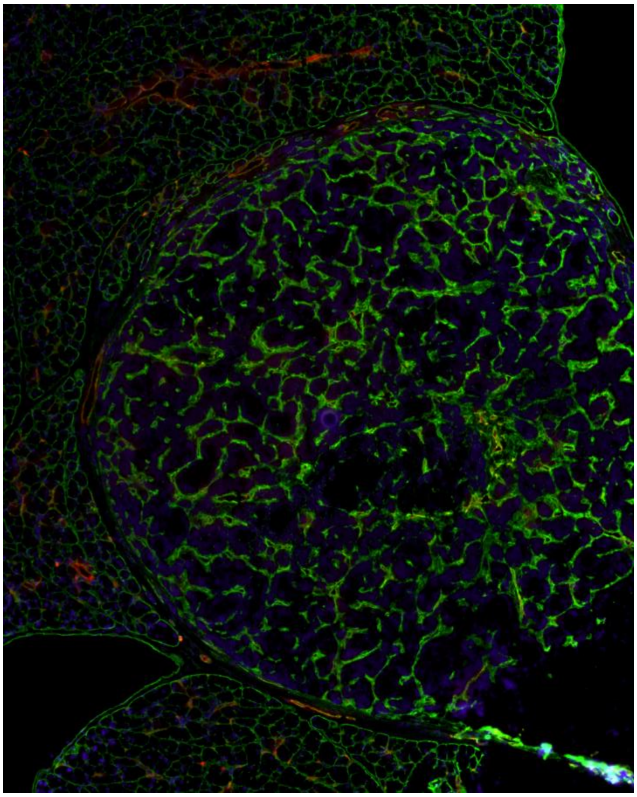

Figure 2 E: Co-staining analysis

RIP1-Tag5 tumour: FAM-CSG/Laminin

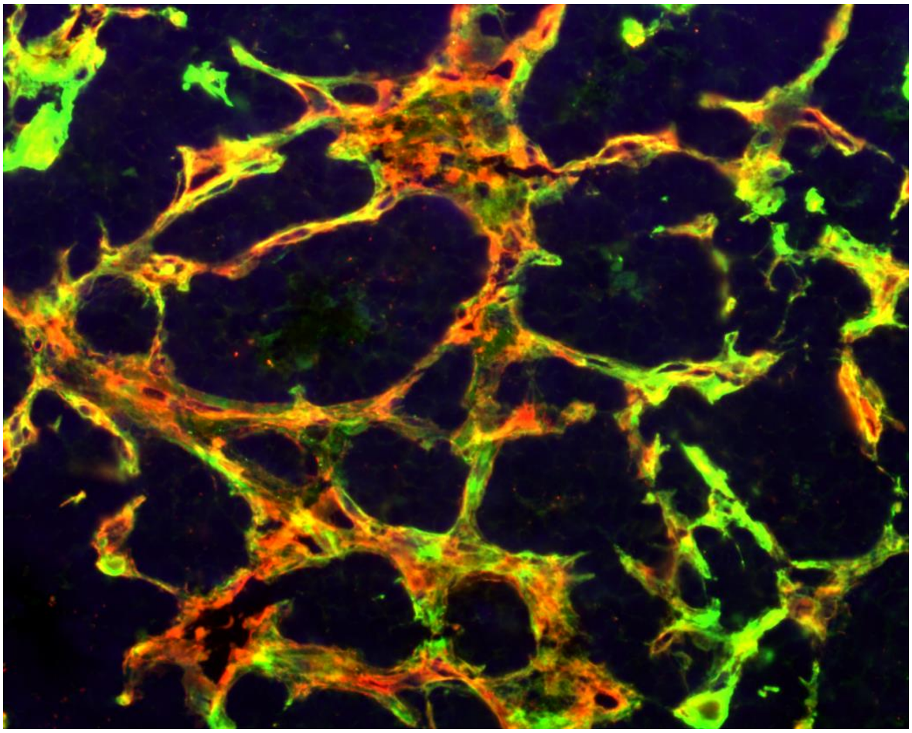

RIP1-Tag5 tumour: FAM-CSG/Nidogen-1

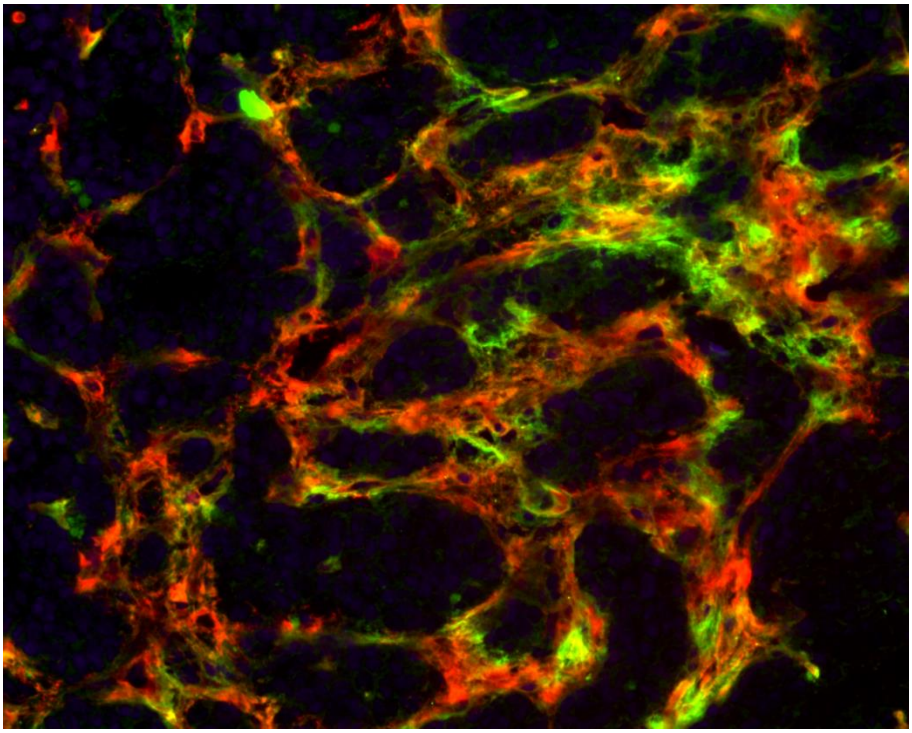

Figure 2 E: Co-staining analysis

RIP1-Tag5 tumour: FAM-CSG/Col-IV

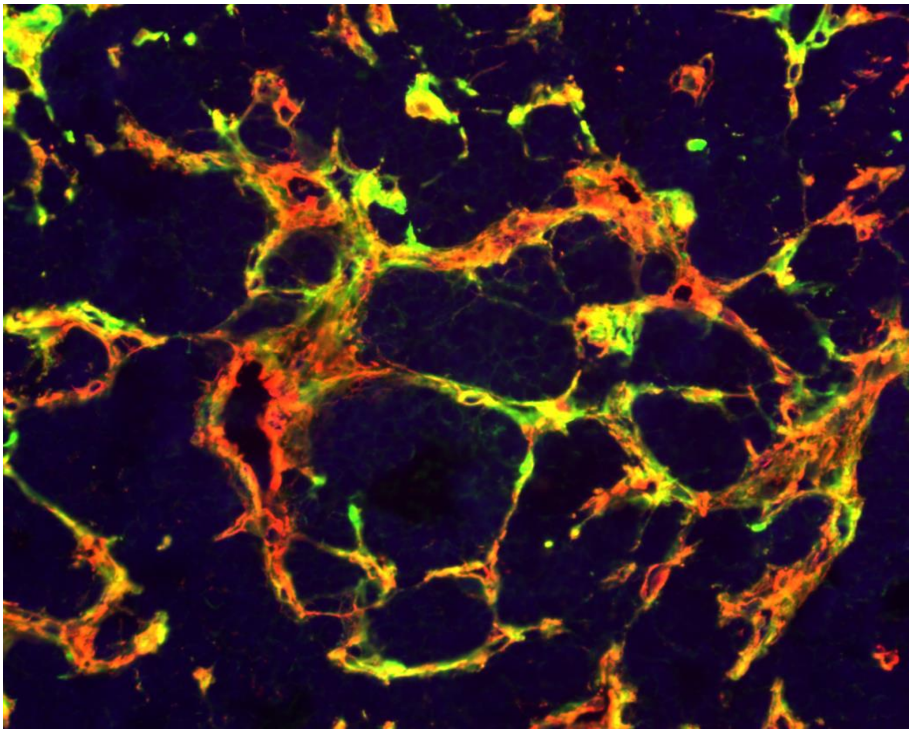

RIP1-Tag5 tumour: FAM-CSG/CD31/DAPI

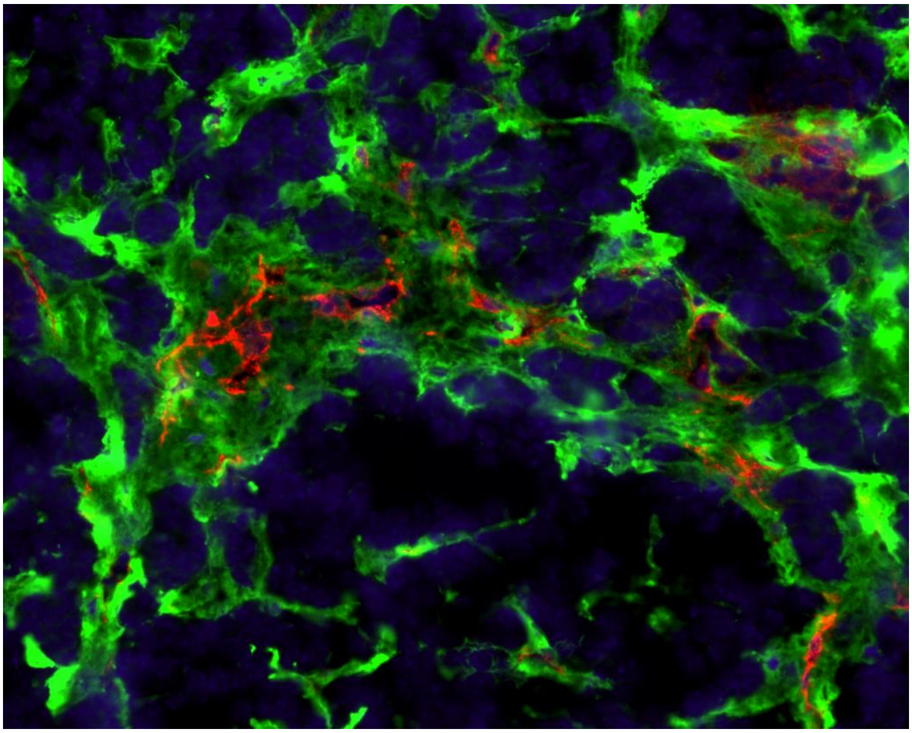

Figure 2 E: Co-staining analysis

RIP1-Tag5 tumour: FAM-CREKA/CD31/DAPI

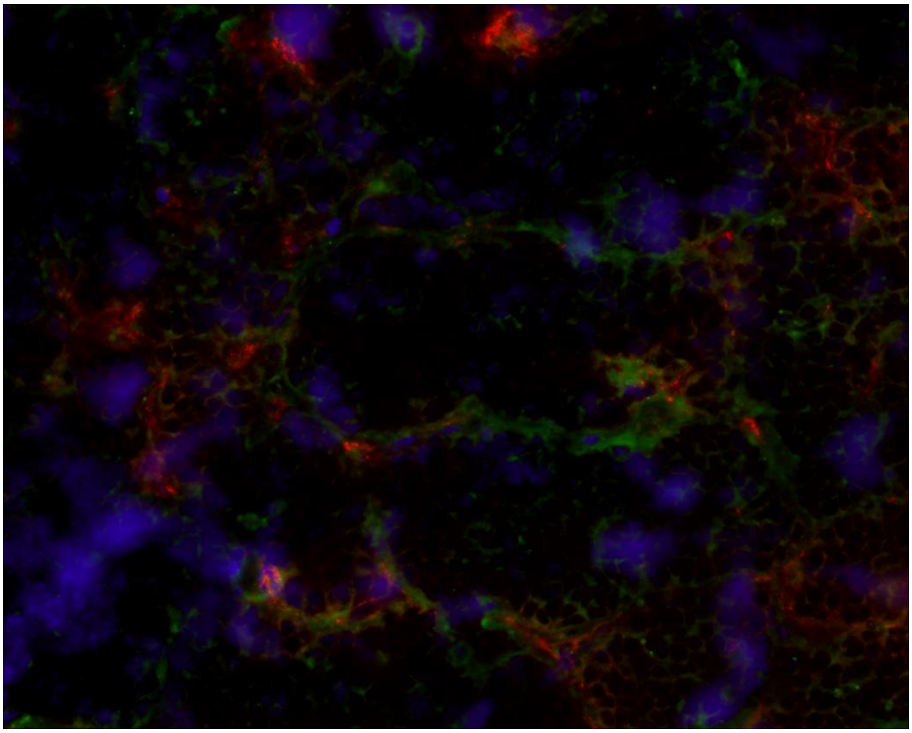

RIP1-Tag5 tumour: FAM-CREKA/Laminin/DAPI

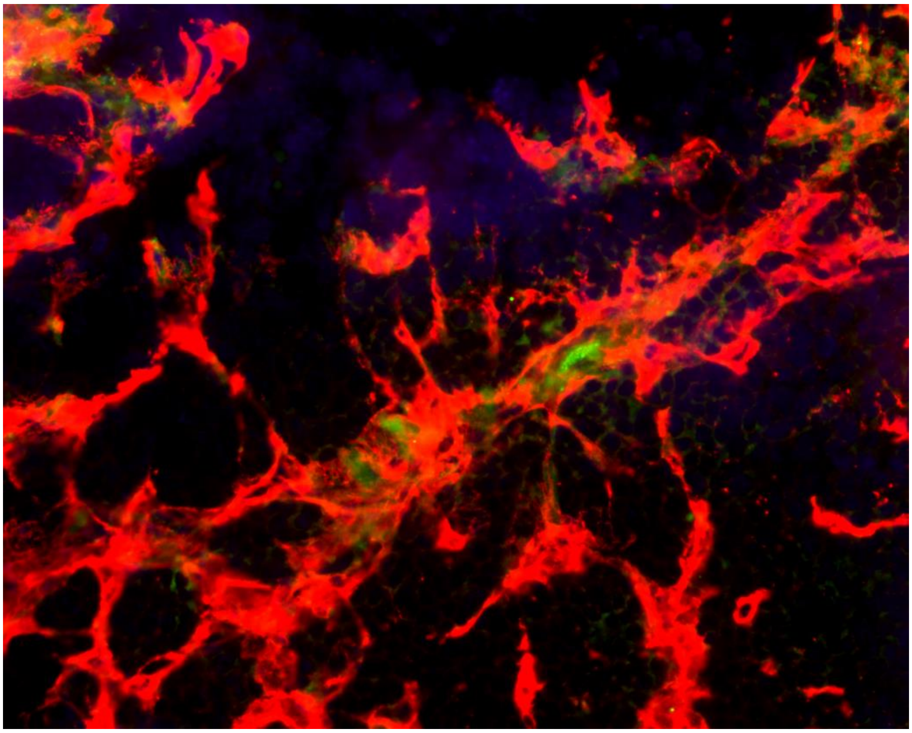

Figure 2B **% staining/field**

**FAM-CSG on Human Normal Breast**	**Pancreas**	**Pancreas**	**Pancreas**	Tumour	**Tumour**	**Tumour**
Laminin	18.6	12.6	10	25.4	27.9	40.3
Nidogen 1	11.4	14.8	10.4	27.3	27.9	35.1
Collagen IV	9.74	10.46	9.78	30.7	39.8	38.4

**Multiple t-tests**	**Significant?**	**P value**	**Mean1**	**Mean2**	**Difference**	**of differer**	**t ratio**	**df**
Laminin	Yes	0.029426579	13.73	31.2	-17.47	5.264	3.318	4
Nidogen 1	Yes	0.003230055	12.2	30.1	-17.9	2.838	6.308	4
Collagen IV	Yes	0.000753965	9.993	36.3	-26.31	2.839	9.267	4

Figure 2C: Ratio Laminin: CD31

**Pancreas**	**Tumour**
7.54	20.36
6.86	8.66
7.86	14.95
2.02	12.42
5.53	17.55

Unpaired t-test

**Table Analyzed**	**Ratio Laminin CD31**
Column B	RT5 T
vs.	vs.
Column A	Pancreas
Unpaired t test	
P value	0.0048
P value summary	**
Significantly different (P < 0.05)?	Yes
One- or two-tailed P value?	Two-tailed
t, df	t=3.861 df=8

Figure 2E: % of colocalisation between 2 markers

**CSG Lam**	**CSG NID1**	**CSG COLIV**	**CSG CD31**	**CREKA Lam**	**CREKA CD31**
98.7	78.8	86	25.7	4.87	10.74
81.9	92.7	79.8	22.8	3.32	5.44
85.5	93.3	61.7	21.3	10.7	24.1
91.4	86.1	77.4	27.3	5.6	8.86

One-way ANOVA Multiple comparison

Number of families	1
Number of comparisons per family	6
Alpha	0.05

**Tukey's multiple comparisons test**	**Mean Diff.**	**95.00% CI of diff.**	**Significant?**	**Summary**	**Adjusted P Value**	
CSG Lam vs. CSG CD31+	65.1	52.75 to 77.45	Yes	****	<0.0001	A-D
CSG Lam vs. CREKA Lam	83.25	70.9 to 95.6	Yes	****	<0.0001	A-E
CSG Lam vs. CREKA CD31	77.09	64.74 to 89.44	Yes	****	<0.0001	A-F
CSG CD31 vs. CREKA Lam	18.15	5.801 to 30.5	Yes	**	0.0044	D-E
CSG CD31 vs. CREKA CD31	11.99	-0.3612 to 24.34	No	ns	0.0581	D-F
CREKA Lam vs. CREKA CD31	-6.163	-18.51 to 6.189	No	ns	0.4773	E-F
