# Supplementary material for: Immune‐mediated ECM depletion improves tumour perfusion and payload delivery
Source: EMBO Mol Med. 2019 Nov 11;11(12):e10923. doi: 10.15252/emmm.201910923 (PMC6895610; doi:10.15252/emmm.201910923)
Supplement: Supplementary file 6 — Source Data for Figure 3 [file EMMM-11-e10923-s005.pdf]

Figure 3A: % CD4/tumour area

Untreated	CSG	TNF -CSG 2 ug	TNF-CSG 5 ug
0.54	0.79	4.76	10.23
3.76	0.63	3.56	4.3
3.71	1.38	6.3	5.09
0.48	3.73	5.93	7
5.6	4.36	5.43	5.14
	3.14	5.7	9
	4.05		

One-way ANOVA multiple comparison

Number of families	1
Number of comparisons per family	6
Alpha	0.05

Tukey's multiple comparisons test	Mean Diff.	95.00% CI of dif	Significant?	Summary	Adjusted P Value	
Untreated vs. CSG	0.264	-2.381 to 2.909	No	ns	0.9923	
Untreated vs. TNF -CSG 2 ug	-3.433	-6.078 to -0.7883	Yes	**	0.0079	
Untreated vs. TNF-CSG 5 ug	-3.888	-6.633 to -1.144	Yes	**	0.0037	
CSG vs. TNF -CSG 2 ug	-3.697	-6.238 to -1.156	Yes	**	0.0029	
CSG vs. TNF-CSG 5 ug	-4.152	-6.797 to -1.508	Yes	**	0.0013	
TNF -CSG 2 ug vs. TNF-CSG 5 ug	-0.4552	-3.1 to 2.19	No	ns	0.9632	

% CD8/tumour area

Untreated	CSG	TNF -CSG 2 ug	TNF-CSG 5 ug
1.09	0.6	6	4.36
0.72	0.35	2.32	4.34
0.49	0.2	2.57	2.53
0.3	0.26	3.5	7.71
2.1	1.02	1.51	2.24
0.33	1.04	8.1	7.18
	0.55	5.9	

Tukey's multiple comparisons test	Mean Diff.	95.00% CI of dif	Significant?	Summary	Adjusted P Value	
Untreated vs. CSG	0.264	-2.381 to 2.909	No	ns	0.9923	
Untreated vs. TNF -CSG 2 ug	-3.433	-6.078 to -0.7883	Yes	**	0.0079	
Untreated vs. TNF-CSG 5 ug	-3.888	-6.633 to -1.144	Yes	**	0.0037	
CSG vs. TNF -CSG 2 ug	-3.697	-6.238 to -1.156	Yes	**	0.0029	
CSG vs. TNF-CSG 5 ug	-4.152	-6.797 to -1.508	Yes	**	0.0013	
TNF -CSG 2 ug vs. TNF-CSG 5 ug	-0.4552	-3.1 to 2.19	No	ns	0.9632	

% CD11b/tumour area

Untreated	CSG	TNF -CSG 2 ug	TNF-CSG 5 ug
0.77	0.51	2.17	4.2
0.61	0.3	3.16	3.8
0.38	0.5	1.85	3.71
1.1	0.25	1.56	4.47
0.3	0.17		3.63
			3.38

Tukey's multiple comparisons test	Mean Diff.	95.00% CI of dif	Significant?	Summary	Adjusted P Value	
Untreated vs. CSG	0.286	-0.465 to 1.037	No	ns	0.7006	
Untreated vs. TNF -CSG 2 ug	-1.553	-2.35 to -0.7565	Yes	***	0.0002	
Untreated vs. TNF-CSG 5 ug	-3.233	-3.952 to -2.514	Yes	****	<0.0001	
CSG vs. TNF -CSG 2 ug	-1.839	-2.636 to -1.042	Yes	****	<0.0001	
CSG vs. TNF-CSG 5 ug	-3.519	-4.238 to -2.8	Yes	****	<0.0001	
TNF -CSG 2 ug vs. TNF-CSG 5 ug	-1.68	-2.446 to -0.9136	Yes	****	<0.0001	

Figure 3B: cDNA/HPRT

CSG				TNF-CSG 2 ug					TNF-CSG 10 ug			
0.0357	0.063	0.1656	0.0967	0.1734	0.1402	0.2604	0.172	0.2149	0.302	0.306	0.2634	0.2208
0.040561	0.072775	0.086074	0.197269	0.013099	0.021948	0.01857	0.02991	0.044378	0.382906	0.504732	0.316803	0.293598
0.071684	0.104613	0.060445	0.171757	0.068004	0.043621	0.150882	0.070429	0.171036	0.541311	0.387224	0.500875	0.318985
0.070408	0.076673	0.105416	0.094143	0.184504	0.167901	0.127902	0.279007	0.192514	1.698575	2.040221	2.035589	1.171082
0.136735	0.237167	0.243472	0.206252	0.28874	0.278189	0.169916	0.386795	0.238787	1.814815	0.487382	1.016919	0.594923
0.079847	0.289799	0.249758	0.285304	0.248885	0.213992	0.353993	0.357675	0.74921	1.376068	1.361199	1.686698	2.908389
0.8196	2.6426	5.007	2.133	3.5697	5.7687	7.782	6.5424	7.006	3.4986	5.4259	4.2573	3.3499
0.7255	1.2164	1.6514	1.9202	1.369	2.0283	3.8716	1.9673	2.3879	3.8917	2.4708	2.6278	3.1921
0.8301	1.3912	1.4956	0.9544	1.6814	1.8277	2.825	2.0954	2.6944	1.3675	1.6932	1.8396	1.4283
0.4125	0.5094	0.9287	0.5264	0.7834	0.6889	0.9285	0.8823	0.7408	0.4085	0.5434	0.5948	0.5717
0.1952	0.4243	1.1248	0.4157	1.8935	1.3764	0.8932	1.3871	1.2287	0.657	1.0804	0.6788	0.5453
0.0992	0.2027	0.2357	0.1171	0.4384	0.2749	0.2637	0.3096	0.391	0.2553	0.4732	0.3305	0.3091
0.2278	0.5036	0.8755	0.2472	0.9418	0.7781	0.8682	0.7745	1.048	0.6661	1.1222	0.8118	0.585
0.7676	1.4035	1.9205	0.8376	1.6686	1.7468	2.4487	1.6659	2.2742	0.9618	1.6293	0.8976	0.4768

Statistical significance determined without correction for multiple comparisons, with alpha=0.05%.
Each row was analyzed individually, without assuming a consistent SD.

CSG vs TNF-CSG 10 ug	Significant?	P value	Mean1	Mean2	Difference	of differen	t ratio	df
uPA	Yes	0.001800467	0.09025	0.2731	-0.1828	0.03438	5.317	6
MMP2	Yes	0.003257315	0.09917	0.3745	-0.2753	0.05833	4.72	6
MMP3	Yes	0.001067796	0.1021	0.4371	-0.335	0.05692	5.885	6
MMP9	Yes	0.000196158	0.08666	1.736	-1.65	0.2049	8.053	6
MMP12	Yes	0.043139863	0.2059	0.9785	-0.7726	0.3023	2.556	6
MMP14	Yes	0.004831219	0.2262	1.833	-1.607	0.3695	4.348	6
Cathepsin B	No	0.186782517	2.651	4.133	-1.482	0.9948	1.49	6
Cathepsin L	Yes	0.006950071	1.378	3.046	-1.667	0.4146	4.021	6
Cathepsin S	No	0.080022985	1.168	1.582	-0.4143	0.1969	2.104	6
Cathepsin C	No	0.614190003	0.5943	0.5296	0.06465	0.1216	0.5315	6
ADAM8	No	0.423719439	0.54	0.7404	-0.2004	0.2335	0.8582	6

ADAM9	Yes	0.020345398	0.1637	0.342	-0.1784	0.057	3.129	6
ADAM10	No	0.133558977	0.4635	0.7963	-0.3328	0.1919	1.734	6
ADAM17b	No	0.528352303	1.232	0.9914	0.2409	0.3601	0.669	6

CSG vs TNF-CSG 2 ug	Significant?	P value	Mean1	Mean2	Difference	of differer	t ratio	df
uPA	Yes	0.02020653	0.09025	0.1922	-0.1019	0.03408	2.991	7
MMP2	Yes	0.046849921	0.09917	0.02558	0.07359	0.03055	2.409	7
MMP3	No	0.97160073	0.1021	0.1008	0.00133	0.03606	0.03689	7
MMP9	Yes	0.009010511	0.08666	0.1904	-0.1037	0.02899	3.577	7
MMP12	No	0.186729314	0.2059	0.2725	-0.06658	0.04549	1.464	7
MMP14	No	0.216158683	0.2262	0.3848	-0.1586	0.1166	1.359	7
Cathepsin B	Yes	0.017104694	2.651	6.134	-3.483	1.12	3.109	7
Cathepsin L	No	0.11649472	1.378	2.325	-0.9464	0.5286	1.79	7
Cathepsin S	Yes	0.009159117	1.168	2.225	-1.057	0.2965	3.565	7
Cathepsin C	No	0.102827108	0.5943	0.8048	-0.2105	0.1122	1.876	7
ADAM8	Yes	0.015047232	0.54	1.356	-0.8158	0.2549	3.201	7
ADAM9	Yes	0.009193964	0.1637	0.3355	-0.1718	0.04824	3.562	7
ADAM10	Yes	0.023466772	0.4635	0.8821	-0.4186	0.1451	2.885	7
ADAM17b	Yes	0.047213101	1.232	1.961	-0.7285	0.3031	2.404	7

Figure 3C: Protease ELISA assay

	PBS 20hr	CSG 4hr	CSG 20hr	TNF-CSG 4hr	TNF-CSG 20hr
CD4: MMP9	1104	1164	1194	1254	1734
	1014	884	1144	1154	1724
	1144	1044	1194	1364	1534
	1154	974	1334	1334	2064
CD4: MMP12	860.3	1254.6	1114.6	914.6	1606
	923.1	1217.4	983.1	1077.4	1614.6
	946	860.3	1123.1	951.7	1620.3
	1174.6	988.9	1094.6	1146	1691.7
CD4: Cathepsin L	0	185.9	0	0	0
	0	91.8	0	91.8	88.8
	0	0	30	0	203.5
	0		0	38.8	174.1
CD8: MMP9	1254	1084	1564	1484	1464
	1424	1404	1414	1634	1184
	1714	1214	1494	1324	1444
	1404	1454	1304	1394	1344
CD8: MMP12	1326	1100.3	1426	1268.9	1377.4
	1417.4	1060.3	1603.1	1346	1286
	1526	1083.1	1460.3	906	1500.3
	1683.1	1157.4	1514.6	988.9	1480.3
CD8: Cathepsin L	0	0	138.8	0	130
	94.7	77.1	0	0	197.6
	62.4	0	162.4	0	394.7
	138.8	0	0	53.5	582.9
CD11b: MMP9	2261.3	1161.3	2130	1323.8	2255
	2280	1055	1961.3	1236.3	2280
	2411.3	1055	2180	1305	2523.8
	2398.8	948.8	2080	1367.5	2511.3
CD11b: MMP12	1965	1150.7	2643.6	1093.6	2065
	1600.7	993.6	1807.9	1029.3	1786.4
	1636.4	657.9	1450.7	1115	1636.4
	1336.4	365	1465	879.3	1315
CD11b: Cathepsin L	127.1	50.6	153.5	38.8	68.2
	71.2	41.8	262.4	138.8	85.9
	50.6	77.1	200.6	121.2	235.9
	88.8	188.8			224.1

One-way ANOVA multiple comparison

Number of families	1
Number of comparisons per family	10
Alpha	0.05

CD4: MMP9

Tukey's multiple comparisons test	Mean Diff.	95.00% CI of dif	Significant?	Summary	Adjusted P Value	
PBS 20hr vs. CSG 4hr	87.5	-192.1 to 367.1	No	ns	0.866	
PBS 20hr vs. CSG 20hr	-112.5	-392.1 to 167.1	No	ns	0.728	
PBS 20hr vs. TNF-CSG 4hr	-172.5	-452.1 to 107.1	No	ns	0.3562	
PBS 20hr vs. TNF-CSG 20hr	-660	-939.6 to -380.4	Yes	****	<0.0001	
CSG 4hr vs. CSG 20hr	-200	-479.6 to 79.64	No	ns	0.229	
CSG 4hr vs. TNF-CSG 4hr	-260	-539.6 to 19.64	No	ns	0.0745	
CSG 4hr vs. TNF-CSG 20hr	-747.5	-1027 to -467.9	Yes	****	<0.0001	
CSG 20hr vs. TNF-CSG 4hr	-60	-339.6 to 219.6	No	ns	0.9615	
CSG 20hr vs. TNF-CSG 20hr	-547.5	-827.1 to -267.9	Yes	***	0.0002	
TNF-CSG 4hr vs. TNF-CSG 20hr	-487.5	-767.1 to -207.9	Yes	***	0.0006	

CD8: MMP9

PBS 20hr vs. CSG 4hr	160	-168.3 to 488.3	No	ns	0.575	
PBS 20hr vs. CSG 20hr	5	-323.3 to 333.3	No	ns	>0.9999	
PBS 20hr vs. TNF-CSG 4hr	-10	-338.3 to 318.3	No	ns	>0.9999	
PBS 20hr vs. TNF-CSG 20hr	90	-238.3 to 418.3	No	ns	0.9117	
CSG 4hr vs. CSG 20hr	-155	-483.3 to 173.3	No	ns	0.6028	
CSG 4hr vs. TNF-CSG 4hr	-170	-498.3 to 158.3	No	ns	0.5201	
CSG 4hr vs. TNF-CSG 20hr	-70	-398.3 to 258.3	No	ns	0.9623	
CSG 20hr vs. TNF-CSG 4hr	-15	-343.3 to 313.3	No	ns	0.9999	
CSG 20hr vs. TNF-CSG 20hr	85	-243.3 to 413.3	No	ns	0.9268	
TNF-CSG 4hr vs. TNF-CSG 20hr	100	-228.3 to 428.3	No	ns	0.8766	

CD11b: MMP9

PBS 20hr vs. CSG 4hr	1283	1073 to 1493	Yes	****	<0.0001	
PBS 20hr vs. CSG 20hr	250	39.76 to 460.3	Yes	*	0.0164	
PBS 20hr vs. TNF-CSG 4hr	1030	819.4 to 1240	Yes	****	<0.0001	

PBS 20hr vs. TNF-CSG 20hr	-54.67	-264.9 to 155.6	No	ns	0.9257	
CSG 4hr vs. CSG 20hr	-1033	-1243 to -822.5	Yes	****	<0.0001	
CSG 4hr vs. TNF-CSG 4hr	-253.1	-463.4 to -42.86	Yes	*	0.0151	
CSG 4hr vs. TNF-CSG 20hr	-1338	-1548 to -1127	Yes	****	<0.0001	
CSG 20hr vs. TNF-CSG 4hr	779.7	569.4 to 989.9	Yes	****	<0.0001	
CSG 20hr vs. TNF-CSG 20hr	-304.7	-515 to -94.44	Yes	**	0.0035	
TNF-CSG 4hr vs. TNF-CSG 20hr	-1084	-1295 to -874.1	Yes	****	<0.0001	

CD4: MMP12

PBS 20hr vs. CSG 4hr	-104.3	-365.5 to 156.9	No	ns	0.7333	
PBS 20hr vs. CSG 20hr	-102.9	-364.1 to 158.4	No	ns	0.7428	
PBS 20hr vs. TNF-CSG 4hr	-46.43	-307.7 to 214.8	No	ns	0.9804	
PBS 20hr vs. TNF-CSG 20hr	-657.2	-918.4 to -395.9	Yes	****	<0.0001	
CSG 4hr vs. CSG 20hr	1.45	-259.8 to 262.7	No	ns	>0.9999	
CSG 4hr vs. TNF-CSG 4hr	57.87	-203.4 to 319.1	No	ns	0.9569	
CSG 4hr vs. TNF-CSG 20hr	-552.9	-814.1 to -291.6	Yes	****	<0.0001	
CSG 20hr vs. TNF-CSG 4hr	56.42	-204.8 to 317.7	No	ns	0.9606	
CSG 20hr vs. TNF-CSG 20hr	-554.3	-815.5 to -293.1	Yes	****	<0.0001	
TNF-CSG 4hr vs. TNF-CSG 20hr	-610.7	-872 to -349.5	Yes	****	<0.0001	

CD8: MMP12

PBS 20hr vs. CSG 4hr	387.9	100.8 to 674.9	Yes	**	0.0062	
PBS 20hr vs. CSG 20hr	-12.88	-300 to 274.2	No	ns	>0.9999	
PBS 20hr vs. TNF-CSG 4hr	360.7	73.58 to 647.8	Yes	*	0.011	
PBS 20hr vs. TNF-CSG 20hr	77.13	-210 to 364.2	No	ns	0.9173	
CSG 4hr vs. CSG 20hr	-400.7	-687.8 to -113.6	Yes	**	0.0048	
CSG 4hr vs. TNF-CSG 4hr	-27.18	-314.3 to 259.9	No	ns	0.9982	
CSG 4hr vs. TNF-CSG 20hr	-310.7	-597.8 to -23.63	Yes	*	0.031	
CSG 20hr vs. TNF-CSG 4hr	373.6	86.45 to 660.6	Yes	**	0.0084	
CSG 20hr vs. TNF-CSG 20hr	90	-197.1 to 377.1	No	ns	0.8652	
TNF-CSG 4hr vs. TNF-CSG 20hr	-283.6	-570.6 to 3.545	No	ns	0.0537	

CD11b:MMP12

PBS 20hr vs. CSG 4hr	842.8	79.17 to 1606	Yes	*	0.0273	
PBS 20hr vs. CSG 20hr	-207.2	-970.8 to 556.5	No	ns	0.9146	
PBS 20hr vs. TNF-CSG 4hr	605.3	-158.3 to 1369	No	ns	0.1559	
PBS 20hr vs. TNF-CSG 20hr	-66.08	-829.7 to 697.6	No	ns	0.9987	
CSG 4hr vs. CSG 20hr	-1050	-1814 to -286.3	Yes	**	0.0054	
CSG 4hr vs. TNF-CSG 4hr	-237.5	-1001 to 526.2	No	ns	0.8684	
CSG 4hr vs. TNF-CSG 20hr	-908.9	-1673 to -145.2	Yes	*	0.0163	
CSG 20hr vs. TNF-CSG 4hr	812.5	48.85 to 1576	Yes	*	0.0345	
CSG 20hr vs. TNF-CSG 20hr	141.1	-622.6 to 904.8	No	ns	0.9774	
TNF-CSG 4hr vs. TNF-CSG 20hr	-671.4	-1435 to 92.25	No	ns	0.0985	

Non-parametric analysis: Kruskal Wallis Test

CD4: Cathepsin L

**Dunn's multiple comparisons test**	**Mean rank diff.**	**Significant?**	**Summary**	**Adjusted P Value**	
PBS 20hr vs. CSG 4hr	-7.167	No	ns	0.6329	
PBS 20hr vs. CSG 20hr	-1.5	No	ns	>0.9999	
PBS 20hr vs. TNF-CSG 4hr	-4.125	No	ns	>0.9999	
PBS 20hr vs. TNF-CSG 20hr	-8	No	ns	0.2514	
CSG 4hr vs. CSG 20hr	5.667	No	ns	>0.9999	
CSG 4hr vs. TNF-CSG 4hr	3.042	No	ns	>0.9999	
CSG 4hr vs. TNF-CSG 20hr	-0.8333	No	ns	>0.9999	
CSG 20hr vs. TNF-CSG 4hr	-2.625	No	ns	>0.9999	
CSG 20hr vs. TNF-CSG 20hr	-6.5	No	ns	0.6885	
TNF-CSG 4hr vs. TNF-CSG 20hr	-3.875	No	ns	>0.9999	

CD8: Cathepsin L

PBS 20hr vs. CSG 4hr	4.375	No	ns	>0.9999	
PBS 20hr vs. CSG 20hr	0.5	No	ns	>0.9999	
PBS 20hr vs. TNF-CSG 4hr	4.875	No	ns	>0.9999	
PBS 20hr vs. TNF-CSG 20hr	-6.625	No	ns	0.9671	
CSG 4hr vs. CSG 20hr	-3.875	No	ns	>0.9999	
CSG 4hr vs. TNF-CSG 4hr	0.5	No	ns	>0.9999	
CSG 4hr vs. TNF-CSG 20hr	-11	No	ns	0.0582	
CSG 20hr vs. TNF-CSG 4hr	4.375	No	ns	>0.9999	
CSG 20hr vs. TNF-CSG 20hr	-7.125	No	ns	0.7403	
TNF-CSG 4hr vs. TNF-CSG 20hr	-11.5	Yes	*	0.0394	

CD11b: Cathepsin L

PBS 20hr vs. CSG 4hr	0.75	No	ns	>0.9999	
PBS 20hr vs. CSG 20hr	-7.958	No	ns	0.5084	
PBS 20hr vs. TNF-CSG 4hr	-0.2917	No	ns	>0.9999	
PBS 20hr vs. TNF-CSG 20hr	-4.125	No	ns	>0.9999	
CSG 4hr vs. CSG 20hr	-8.708	No	ns	0.3261	
CSG 4hr vs. TNF-CSG 4hr	-1.042	No	ns	>0.9999	
CSG 4hr vs. TNF-CSG 20hr	-4.875	No	ns	>0.9999	
CSG 20hr vs. TNF-CSG 4hr	7.667	No	ns	0.7845	
CSG 20hr vs. TNF-CSG 20hr	3.833	No	ns	>0.9999	
TNF-CSG 4hr vs. TNF-CSG 20hr	-3.833	No	ns	>0.9999	
