# Supplementary material for: Immune‐mediated ECM depletion improves tumour perfusion and payload delivery
Source: EMBO Mol Med. 2019 Nov 11;11(12):e10923. doi: 10.15252/emmm.201910923 (PMC6895610; doi:10.15252/emmm.201910923)
Supplement: Supplementary file 7 — Source Data for Figure 4 [file EMMM-11-e10923-s006.pdf]

Figure 4A RIP1-Tag 5

Laminin

CSG

TNF-RGR

TNF-CSG

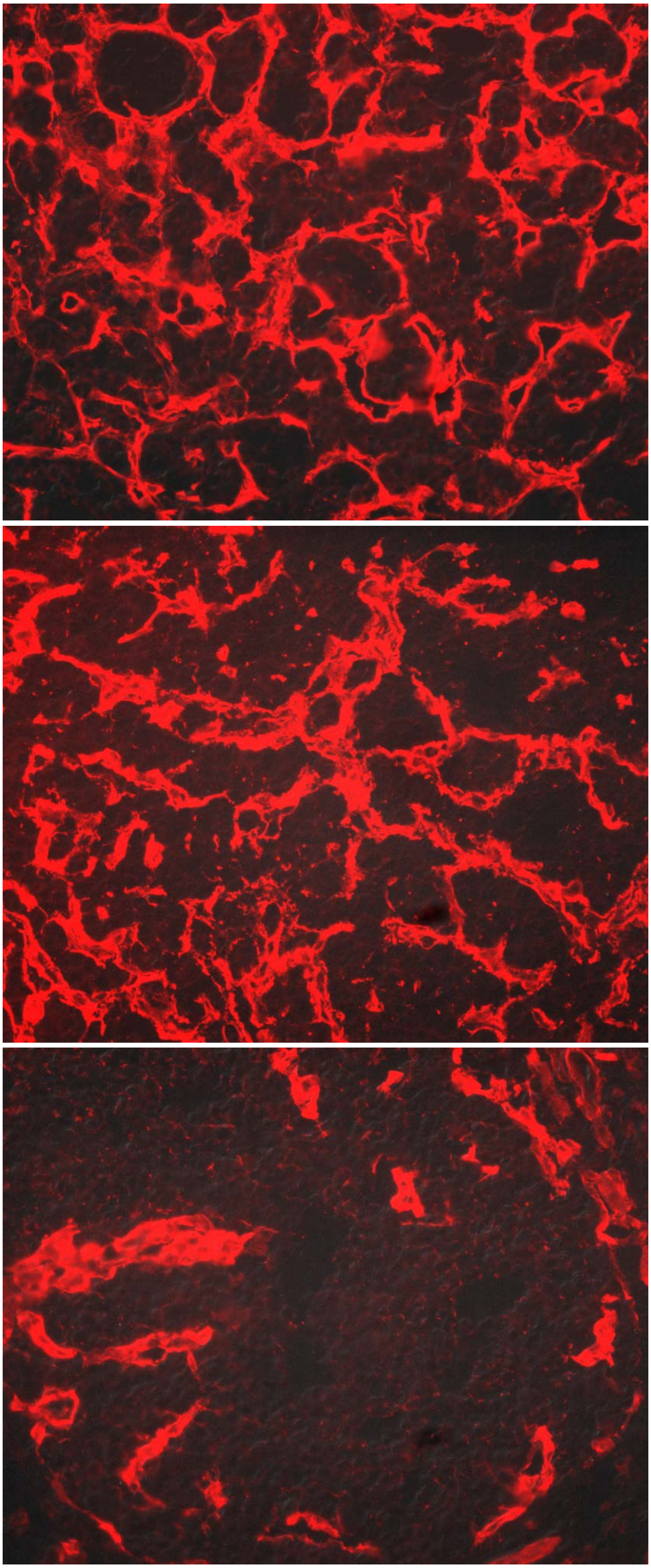

Figure 4A RIP1-Tag 5

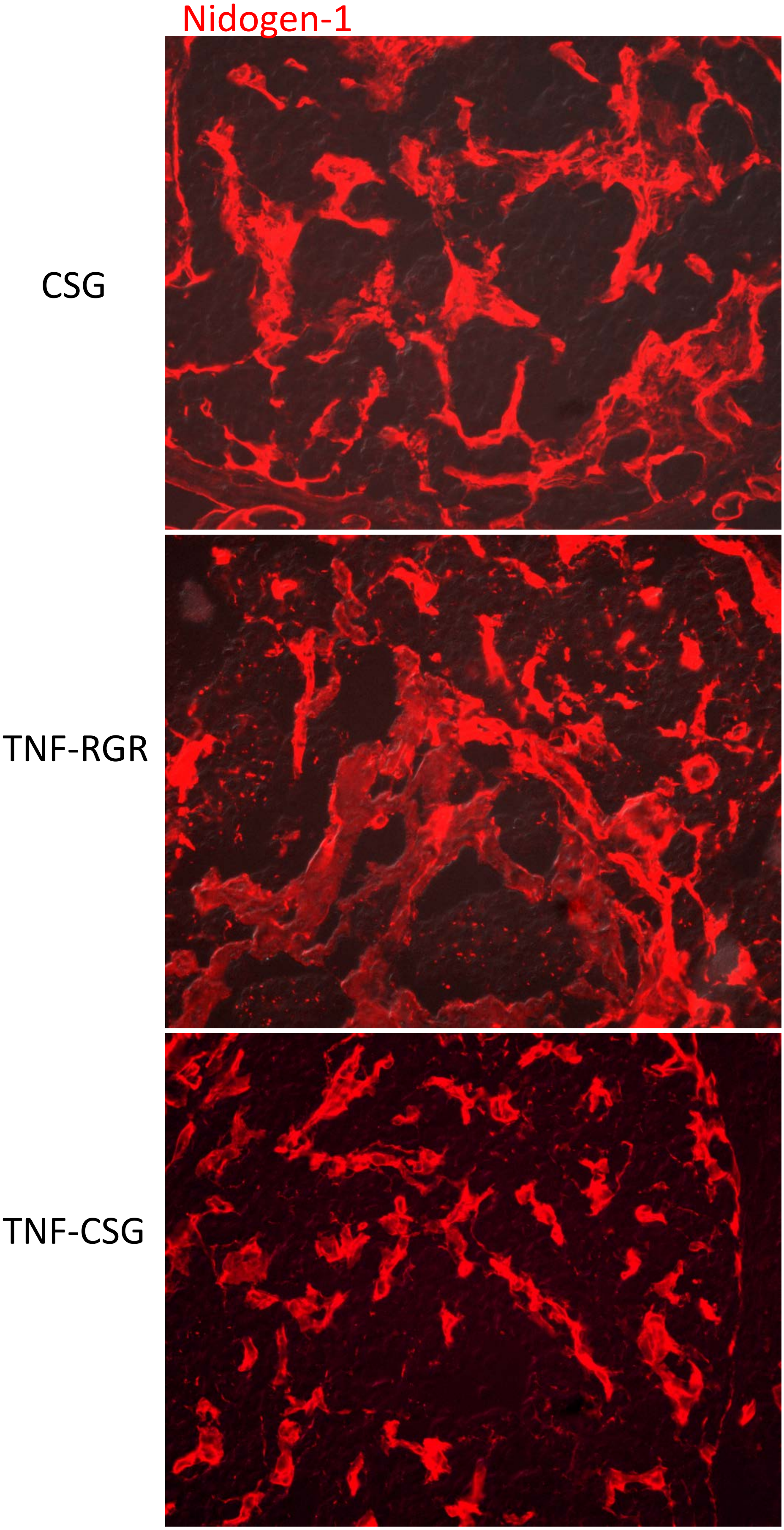

Figure 4A RIP1-Tag 5

Collagen-IV

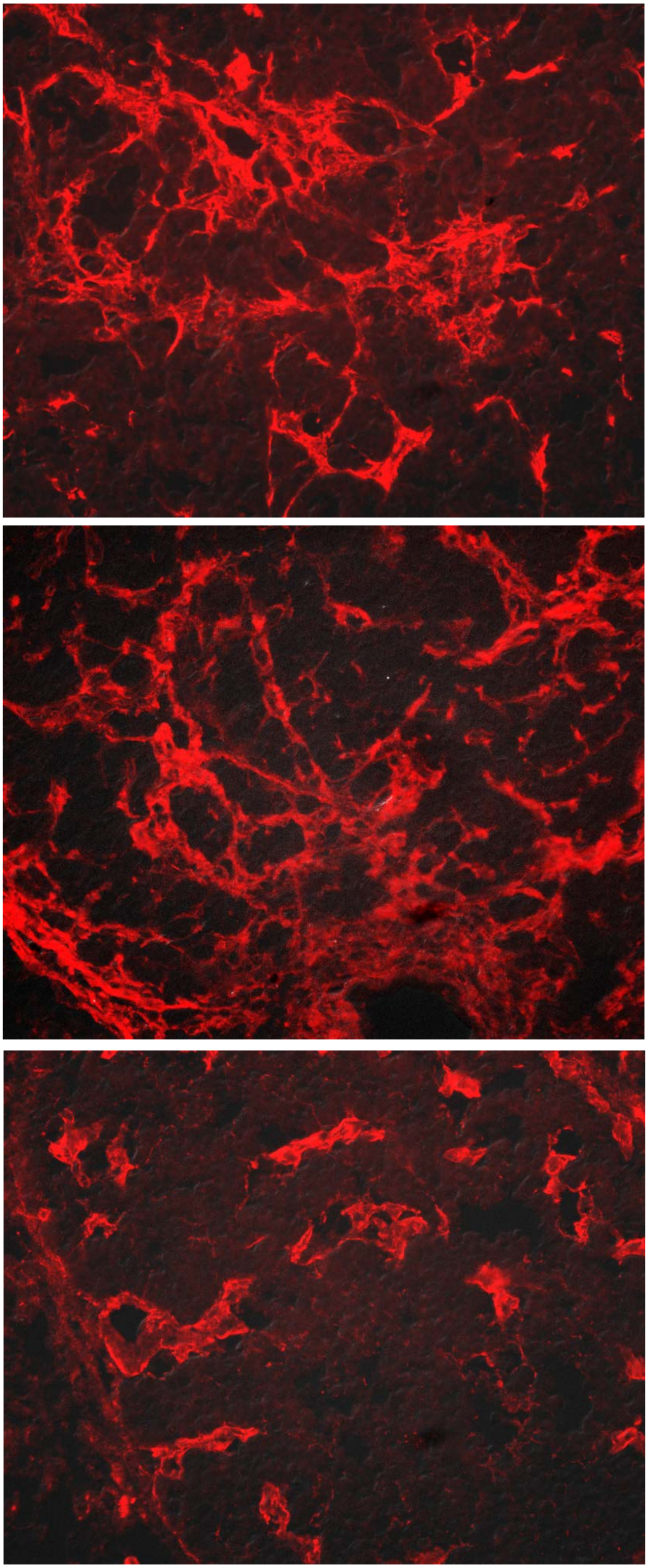

CSG

TNF-RGR

TNF-CSG

Figure 4B: % of staining/area

Laminin

Untreated	CSG	TNF-RGR 5 ug	TNF-CSG 2 ug	TNF-CSG 5 ug
18.65	16.6	9.67	5.36	8.15
17.37	13.29	11.07	11.69	8.48
15.55	17.99	14.01	11	6.25
	11.14	22.79	7.04	12.24
	14.31	13.7	10.22	8.44
	12.56	12.64	10.09	8.03
		20.6		
		16.28		

Nidogen 1

Untreated	CSG	TNF-RGR 5 ug	TNF-CSG 2 ug	TNF-CSG 5 ug
14.77	16.3	16.42	12.37	12.08
15.32	11.37	20.19	6.25	5.12
17.66	15.17	14.72	8.1	9.97
	14.76	15.71	7.2	10.5
	20.63	20.7	11.05	10.72
	19.73	12.33	10.2	11.23
		12.16		
		16.15		

Untreated	CSG	TNF-RGR 5 ug	TNF-CSG 2 ug	TNF-CSG 5 ug
19.2	11.76	12.77	5.82	11.2
11.33	12.2	13.3	6.66	8.3
17.4	15.55	10.7	7.98	5.99
	18.2	16.86	10.33	11.67
	12.67	17.22	10.19	10.29
	21.23	20.75	7.22	5.62
		19.59		

One-way ANOVA multiple comparison

Tukey's multiple comparisons test	Mean Diff.	95.00% CI of diff.	Significant?	Summary	Adjusted P Value	
**Laminin**						
Untreated vs. CSG	2.875	-3.623 to 9.3	No	ns	0.6917	
Untreated vs. TNF-RGR 5 ug	2.095	-4.126 to 8.3	No	ns	0.8563	
Untreated vs. TNF-CSG 2 ug	7.957	1.459 to 14.4	Yes	*	0.0112	
Untreated vs. TNF-CSG 5 ug	8.592	2.094 to 15.0	Yes	**	0.0056	
CSG vs. TNF-RGR 5 ug	-0.78	-5.743 to 4.1	No	ns	0.9899	
CSG vs. TNF-CSG 2 ug	5.082	-0.2238 to 1(	No	ns	0.0651	
CSG vs. TNF-CSG 5 ug	5.717	0.4112 to 11	Yes	*	0.0303	
TNF-RGR 5 ug vs. TNF-CSG 2 ug	5.862	0.8988 to 10	Yes	*	0.0151	
TNF-RGR 5 ug vs. TNF-CSG 5 ug	6.497	1.534 to 11.4	Yes	**	0.0062	
TNF-CSG 2 ug vs. TNF-CSG 5 ug	0.635	-4.67 to 5.94	No	ns	0.9965	
**Nidogen-1**						
Untreated vs. CSG	-0.41	-6.298 to 5.4	No	ns	0.9996	
Untreated vs. TNF-RGR 5 ug	-0.1308	-5.768 to 5.5	No	ns	>0.9999	
Untreated vs. TNF-CSG 2 ug	6.722	0.8339 to 12	Yes	*	0.0197	
Untreated vs. TNF-CSG 5 ug	5.98	0.09225 to 1	Yes	*	0.0453	

CSG vs. TNF-RGR 5 ug	0.2792	-4.218 to 4.7	No	ns	0.9997	
CSG vs. TNF-CSG 2 ug	7.132	2.324 to 11.9	Yes	**	0.0018	
CSG vs. TNF-CSG 5 ug	6.39	1.583 to 11.2	Yes	**	0.0053	
TNF-RGR 5 ug vs. TNF-CSG 2 ug	6.853	2.356 to 11.3	Yes	**	0.0013	
TNF-RGR 5 ug vs. TNF-CSG 5 ug	6.111	1.614 to 10.6	Yes	**	0.0043	
TNF-CSG 2 ug vs. TNF-CSG 5 ug	-0.7417	-5.549 to 4.0	No	ns	0.9906	
**Colagen IV**						
Untreated vs. CSG	0.7083	-6.071 to 7.4	No	ns	0.9979	
Untreated vs. TNF-RGR 5 ug	0.09238	-6.523 to 6.7	No	ns	>0.9999	
Untreated vs. TNF-CSG 2 ug	7.943	1.164 to 14.7	Yes	*	0.0163	
Untreated vs. TNF-CSG 5 ug	7.132	0.3525 to 13	Yes	*	0.0359	
CSG vs. TNF-RGR 5 ug	-0.616	-5.95 to 4.71	No	ns	0.9969	
CSG vs. TNF-CSG 2 ug	7.235	1.7 to 12.77	Yes	**	0.0064	
CSG vs. TNF-CSG 5 ug	6.423	0.8882 to 11	Yes	*	0.0175	
TNF-RGR 5 ug vs. TNF-CSG 2 ug	7.851	2.517 to 13.1	Yes	**	0.002	
TNF-RGR 5 ug vs. TNF-CSG 5 ug	7.039	1.705 to 12.3	Yes	**	0.0058	
TNF-CSG 2 ug vs. TNF-CSG 5 ug	-0.8117	-6.347 to 4.7	No	ns	0.9921	

Figure 4D: Correlation CD45+ cells and COL-IV staining

COL-IV	CD45+
19.735	7.228
7.153	10.898
3.65	13.7
30.12	6.06
1.457	12.178
13.828	7.932
2.533	13.55
21.143	6.8
7.544	7.237
3.043	8.927

Spearman r	
r	-0.8788
95% confidence interval	
P value	
P (two-tailed)	0.0016
P value summary	**
Exact or approximate P value?	Exact
Significant? (alpha = 0.05)	Yes
Number of XY Pairs	10
