# Supplementary material for: Immune‐mediated ECM depletion improves tumour perfusion and payload delivery
Source: EMBO Mol Med. 2019 Nov 11;11(12):e10923. doi: 10.15252/emmm.201910923 (PMC6895610; doi:10.15252/emmm.201910923)
Supplement: Supplementary file 8 — Source Data for Figure 5 [file EMMM-11-e10923-s007.pdf]

Figure 5D: 4T1 tumour >100 kPa

CSG	TNF-CSG
10.56	0.8
4.57	1.33
5.6	0.68

Figure 5D: 4T1 tumour stiffness variance

CSG	TNF-CSG
0.279	0.132
0.249	0.181
0.25	0.078

Unpaired T test

Table Analyzed	4T1 >100 kPa	Table Analyzed	4T1 variance
Column B	4T1-TNF-CSG	Column B	4T1-TNF-CSG
vs.	vs.	vs.	vs.
Column A	4T1-CSG	Column A	4T1-CSG
Unpaired t test		Unpaired t test	
P value	0.0325	P value	0.0146
P value summary	*	P value summary	*
Significantly different (P < 0.05)?	Yes	Significantly different (P < 0.05)?	Yes
One- or two-tailed P value?	Two-tailed	One- or two-tailed P value?	Two-tailed
t, df	t=3.212 df=4	t, df	t=4.117 df=4
How big is the difference?		How big is the difference?	
Mean ± SEM of column A	6.91 ± 1.849, n=3	Mean ± SEM of column A	0.2593 ± 0.009838, n=3
Mean ± SEM of column B	0.9367 ± 0.1997, n=3	Mean ± SEM of column B	0.1303 ± 0.02975, n=3
Difference between means	-5.973 ± 1.86	Difference between means	-0.129 ± 0.03133
95% confidence interval	-11.14 to -0.8097	95% confidence interval	-0.216 to -0.04201
R squared (eta squared)	0.7206	R squared (eta squared)	0.8091
F test to compare variances		F test to compare variances	
F, DFn, Dfd	85.74, 2, 2	F, DFn, Dfd	9.142, 2, 2
P value	0.0231	P value	0.1972
P value summary	*	P value summary	ns
Significantly different (P < 0.05)?	Yes	Significantly different (P < 0.05)?	No
