# Supplementary material for: Immune‐mediated ECM depletion improves tumour perfusion and payload delivery
Source: EMBO Mol Med. 2019 Nov 11;11(12):e10923. doi: 10.15252/emmm.201910923 (PMC6895610; doi:10.15252/emmm.201910923)
Supplement: Supplementary file 9 — Source Data for Figure 6 [file EMMM-11-e10923-s008.pdf]

Figure 6A

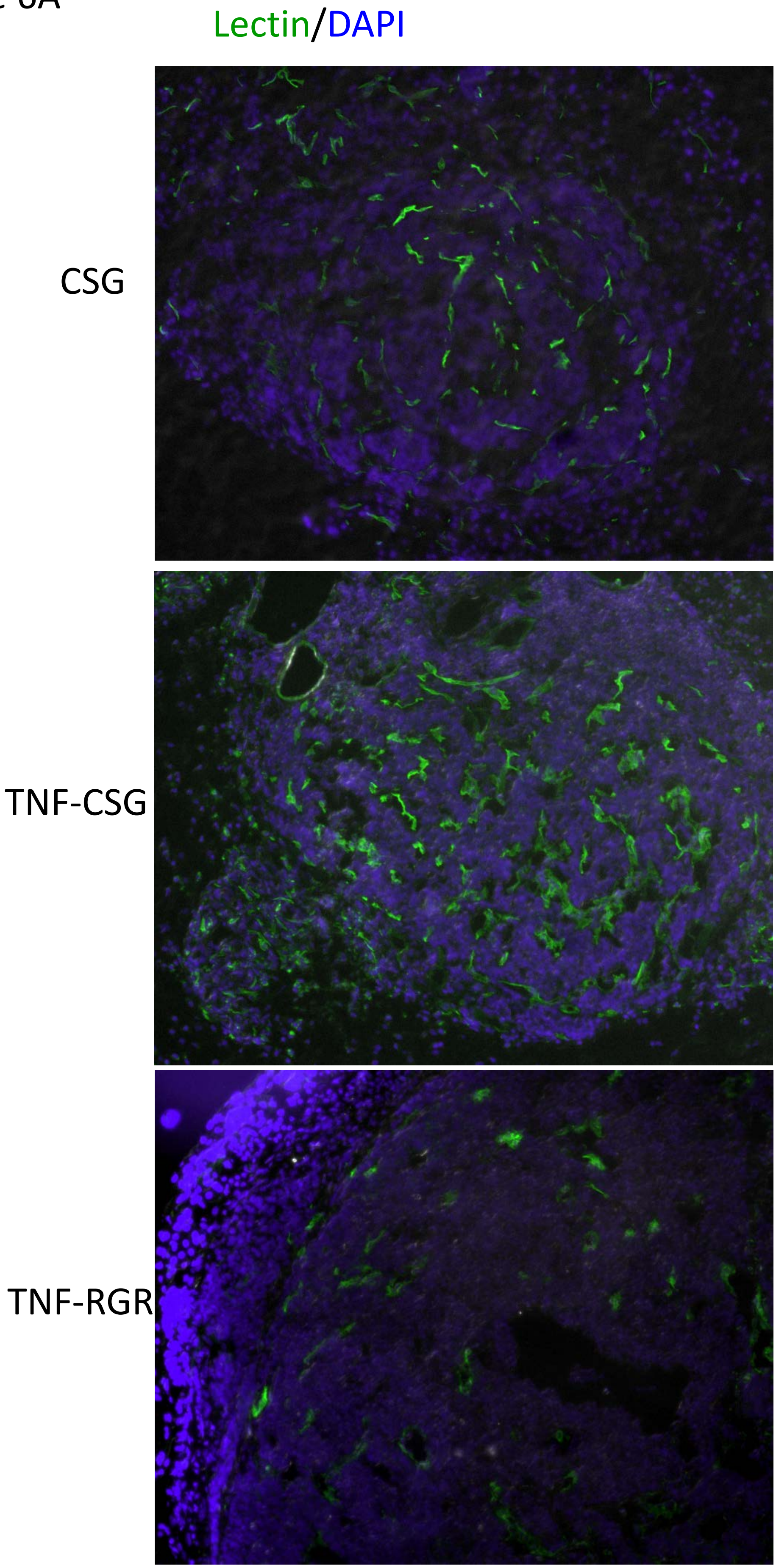

Figure 6B

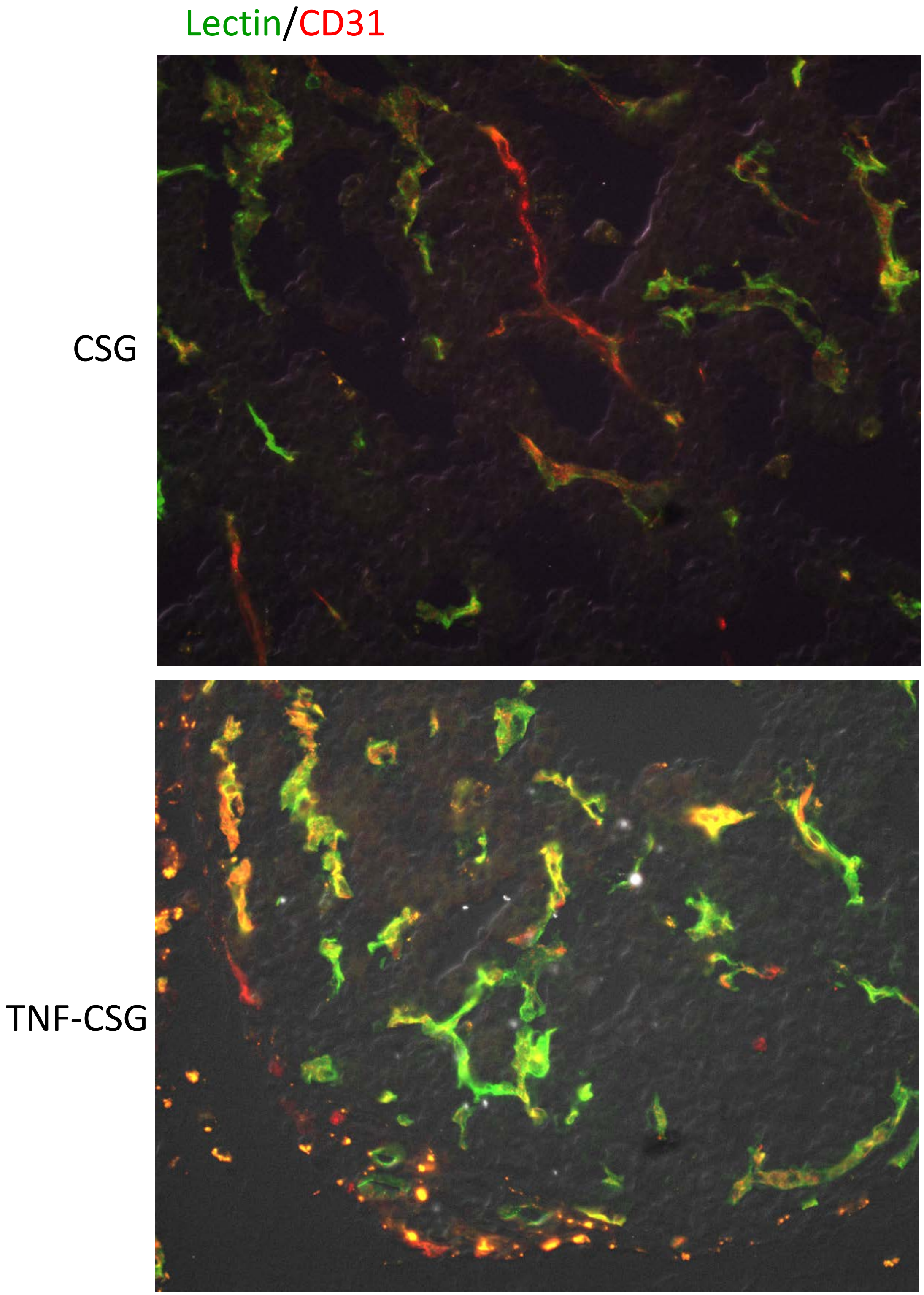

Figure 6C

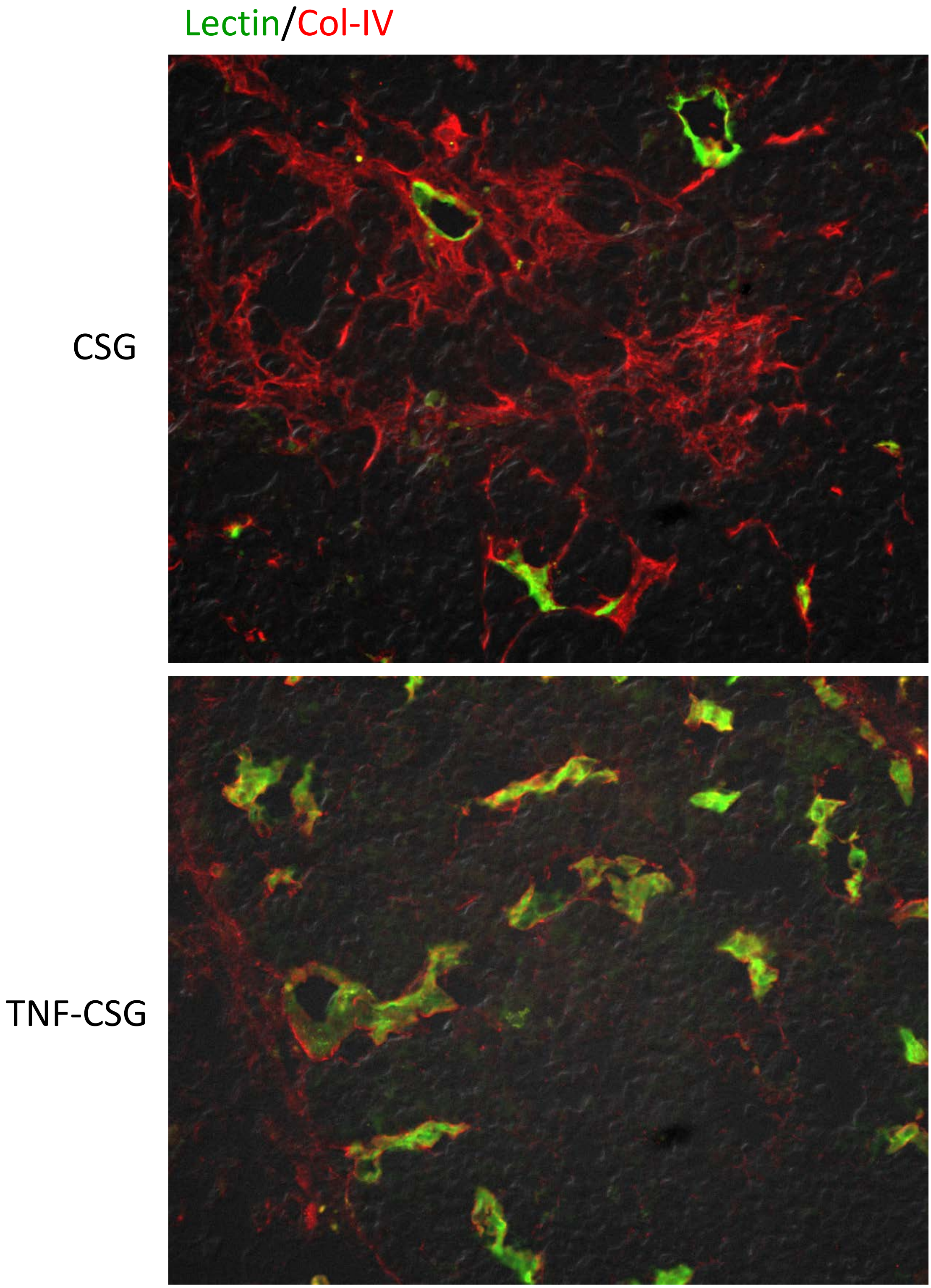

Figure 6A: Mean vessel diameter/tumour

PBS	CSG	TNF-RGR	TNF-CSG 5 ug
6.9265	5.855714	5.075294	15.15684
4.174615	5.569048	6.57	14.245
4.237692	5.063077	4.036	6.917143
7.085882	7.435	5.165	18.015
5.6785	4.772	4.487273	18.53375
5.365	5.114167	4.511818	8.907778
7.417	6.249412	3.2475	14.112
2.61	5.731667	7.45	15.85692
2.350588	4.885334	5.78	12.65769
4.32	4.667619	5.2	12.295
5.301875	5.716667	3.32	11.57286
5.270769	4.137143	2.17	15.97429
			13.89125
			10.35571
			18.715

One-way ANOVA multiple comparison

Number of families	1
Number of comparisons	6
Alpha	0.05

Tukey's multiple comparisons test	Mean Diff.	95.00% CI of diff.	Significant?	Summary	Adjusted P Value	
PBS vs. CSG	-0.3715	-2.768 to 2.0	No	ns	0.976	
PBS vs. TNF-RGR	0.3105	-2.086 to 2.7	No	ns	0.9857	
PBS vs. TNF-CSG 5 ug	-8.752	-11.03 to -6.4	Yes	****	<0.0001	
CSG vs. TNF-RGR	0.682	-1.714 to 3.0	No	ns	0.8727	
CSG vs. TNF-CSG 5 ug	-8.381	-10.65 to -6.1	Yes	****	<0.0001	
TNF-RGR vs. TNF-CSG	-9.063	-11.34 to -6.7	Yes	****	<0.0001	

Figure 6B: Ratio Lectin: CD31

Untreated	CSG	TNF-CSG2 ug	TNF-CSG 5 ug
0.8746594	0.8107527	1.017978	1.360656
0.531854	0.730631	0.9949324	1.197379
0.7133758	0.7985294	1.029126	1.75502
0.8688712	0.8814815	1.01222	1.188848
0.7438825	0.6058172	1.182712	0.8644068
			1.154545
			1.035565

One-way ANOVA multiple comparison

Number of families	1
Number of comparisons	6
Alpha	0.05

Tukey's multiple comparisons test	Mean Diff.	95.00% CI of diff.	Significant?	Summary	Adjusted P Value	
Untreated vs. CSG	-0.01891	-0.35 to 0.31	No	ns	0.9984	
Untreated vs. TNF-CSG2	-0.3009	-0.632 to 0.0	No	ns	0.0826	
Untreated vs. TNF-CSG	-0.4758	-0.7824 to -0	Yes	**	0.0018	
CSG vs. TNF-CSG2 ug	-0.282	-0.6131 to 0.	No	ns	0.1115	

CSG vs. TNF-CSG 5 ug	-0.4569	-0.7634 to -0	Yes	**	0.0027	
TNF-CSG2 ug vs. TNF-C	-0.175	-0.4815 to 0.	No	ns	0.3965	

Figure 6C: Correlation lectin and Collagen IV

	Collagen IV	Lectin
Untreated	19.2	2.99
Untreated	11.33	1.79
Untreated	17.4	2.15
CSG	11.76	1.9
CSG	12.2	3.41
CSG	15.55	1.56
CSG	18.2	3.55
CSG	12.67	3.33
CSG	21.23	2.11
TNF-RGR	12.77	1.73
TNF-RGR	13.3	0.62
TNF-RGR	10.7	1.28
TNF-RGR	16.86	2.32
TNF-RGR	17.22	1.4
TNF-RGR	20.75	3.7
TNF-RGR	19.59	2.7
TNF-CSG	5.82	4.34
TNF-CSG	6.66	4.42
TNF-CSG	7.98	5.7
TNF-CSG	10.33	3.76
TNF-CSG	10.19	3.33
TNF-CSG	7.22	4.67
TNF-CSG	11.2	3.33
TNF-CSG	8.3	5.4
TNF-CSG	5.99	5.51
TNF-CSG	11.67	4.87
TNF-CSG	10.29	7.57
TNF-CSG	5.62	5.58

**Pearson r**		
r	-0.5427	
95% confidence interval	-0.7616 to -0.2126	
R squared	0.2945	
P value		
P (two-tailed)	0.0028	
P value summary	**	
Significant? (alpha = 0.05	Yes	
Number of XY Pairs	28	

Figure 6D: Kinetic constant

CSG	TNFa-CSG
0.042831	0.082653
0.064268	0.085991
0.052241	0.12851
0.030594	0.083735
	0.066646

Unpaired t-test

Table Analyzed	Kinetic constant	
Column B	TNFa-CSG	
vs.	vs.	
Column A	CSG	
Unpaired t test		
P value	0.0159	
P value summary	*	
Significantly different (P <	Yes	
One- or two-tailed P valu	Two-tailed	
t, df	t=3.162 df=7	
How big is the difference?		
Mean ± SEM of column A	0.04748 ± 0.007137, n=4	
Mean ± SEM of column B	0.08951 ± 0.01034, n=5	
Difference between mea	0.04202 ± 0.01329	
95% confidence interval	0.0106 to 0.07345	
R squared (eta squared)	0.5882	

Figure 6E: T2 relaxation time (ms)/tumour)

**CSG**	**TNF-CSG 10 ug**
50.18273	39.085
61.89	42.73667
52.93	31.065
63.02	35.85
53.66	41.775
	37.675
	43.28
	43.06
	35.52
	35.61

Unpaired t-test

Table Analyzed	RIPTag5	
Column B	TNF-CSG 10 ug	
vs.	vs.	
Column A	CSG	
Unpaired t test		
P value	<0.0001	
P value summary	****	
Significantly different (P <	Yes	
One- or two-tailed P valu	Two-tailed	
t, df	t=6.93 df=13	
How big is the difference?		
Mean ± SEM of column A	56.34 ± 2.57, n=5	
Mean ± SEM of column B	38.57 ± 1.303, n=10	
Difference between mea	-17.77 ± 2.564	
95% confidence interval	-23.31 to -12.23	

R squared (eta squared)	0.787	

Figure 6F: % IO NPs accumulation in tumours (anti-FITC)

CSG	TNFa-CSG
0.1	19.2
0.2	2.9
0.2	3
3.8	1.6
4	5.2
4	6
	10.1
	10.7
	8.8

Unpaired t-test

**Table Analyzed**	**IO micelles uptake**	
Column B	TNFa-CSG	
vs.	vs.	
Column A	CSG	
Unpaired t test		
P value	0.038	
P value summary	*	
Significantly different (P <	Yes	
One- or two-tailed P valu	Two-tailed	
t, df	t=2.309 df=13	
How big is the difference?		
Mean ± SEM of column A	2.05 ± 0.8429, n=6	
Mean ± SEM of column B	7.5 ± 1.823, n=9	
Difference between mea	5.45 ± 2.36	
95% confidence interval	0.3516 to 10.55	
R squared (eta squared)	0.2909	
F test to compare variances		
F, DFn, Dfd	7.018, 8, 5	
P value	0.0461	
P value summary	*	
Significantly different (P <	Yes	
