# Supplementary material for: Immune‐mediated ECM depletion improves tumour perfusion and payload delivery
Source: EMBO Mol Med. 2019 Nov 11;11(12):e10923. doi: 10.15252/emmm.201910923 (PMC6895610; doi:10.15252/emmm.201910923)
Supplement: Supplementary file 10 — Source Data for Figure 7 [file EMMM-11-e10923-s009.pdf]

Figure 7A: Survival of RIP1-Tag5 mice (weeks)

**Untreated**	25.7
**Untreated**	27
**Untreated**	27.1
**Untreated**	27.3
**Untreated**	27.4
**Untreated**	27.9
**Untreated**	29.6
**Untreated**	30
**Untreated**	30
**Untreated**	30.1
**Untreated**	30.4
**Untreated**	30.4
**Untreated**	31
**Untreated**	31
**Untreated**	31
**Untreated**	31
**Untreated**	26.6
**Untreated**	26.6
**Untreated**	26.7
**Untreated**	26.7
**Untreated**	26.7
**Untreated**	26.9
**Untreated**	27.3
**Untreated**	28.1
**Untreated**	28.4
**Untreated**	28.6
**Untreated**	29.4

**TNF-CSG 5 ug**	33.6
**TNF-CSG 5 ug**	29.9
**TNF-CSG 5 ug**	32.6
**TNF-CSG 5 ug**	32.7
**TNF-CSG 5 ug**	32.3
**TNF-CSG 5 ug**	30.7
**TNF-CSG 5 ug**	29.9
**TNF-CSG 5 ug**	31.9
**TNF-CSG 5 ug**	28.4
**TNF-CSG 5 ug**	26.4
**TNF-CSG 5 ug**	29.7
**TNF-CSG 5 ug**	30.7
**TNF-CSG 5 ug**	30.3
**TNF-CSG 5 ug**	31.7
**TNF-CSG 5 ug**	35.4
**TNF-CSG 5 ug**	36

Comparison of Survival Curves

**Log-rank (Mantel-Cox) test**		
Chi square	14.23	
df	1	
P value	0.0002	
P value summary	***	
sig different?	Yes	
Gehan-Breslow-Wilcoxon test		
Chi square	11.42	
df	1	
P value	0.0007	
P value summary	***	
sig different?	Yes	
Median survival		
Untreated	28.1	
TNF-CSG5 ug	31.2	
Ratio (and its reciprocal	0.9006	1.11
95% CI of ratio	0.4853 to 1.672	0.5983 to 2.061
Hazard Ratio (Mantel-Ha	A/B	B/A
Ratio (and its reciprocal	3.837	0.2606
95% CI of ratio	1.908 to 7.716	0.1296 to 0.5242

Hazard Ratio (logrank)	A/B	B/A
Ratio (and its reciprocal	2.732	0.366
95% CI of ratio	1.477 to 5.055	0.1978 to 0.6771

Figure 7B: Survival of 4T1 tumour-bearing mice (days)

**Untreated**	16
**Untreated**	18
**Untreated**	18
**Untreated**	18
**Untreated**	19
**Untreated**	19
**Untreated**	20

**TNF CSG 2 ug**	22
**TNF CSG 2 ug**	23
**TNF CSG 2 ug**	23
**TNF CSG 2 ug**	25
**TNF CSG 2 ug**	26

**Comparison of Survival Curves**			
Log-rank (Mantel-Cox) test			
Chi square	10.83		
df	1		
P value	0.001		
P value summary	**		
Are the survival curves sig different?	Yes		
Gehan-Breslow-Wilcoxon test			
Chi square	8.895		
df	1		
P value	0.0029		
P value summary	**		
Are the survival curves sig different?	Yes		
Median survival			
Untreated	18		
TNF-CSG	23		
Ratio (and its reciprocal)	0.7826	1.278	
95% CI of ratio	0.2484 to 2.466	0.4055 to 4.026	
Hazard Ratio (Mantel-Ha	A/B	B/A	
Ratio (and its reciprocal)	16.48	0.06067	
95% CI of ratio	3.105 to 87.48	0.01143 to 0.3221	
Hazard Ratio (logrank)	A/B	B/A	
Ratio (and its reciprocal)	3.957	0.2527	
95% CI of ratio	1.092 to 14.34	0.06973 to 0.9161	

Figure 7C: tumour volume

**Untreated**	**CSG**	**TNF-CSG 2 ug**
386.5375	486.5255	375.17
247.009	318.3236	158.3839
443.576	381.8561	91.39235
332.82	227.8194	66.67699
690.9705	234.4322	69.02592
	648	247.45

	433.5	364.5
	281.75	100
	368	100
	379.75	171.5
		245

One-way ANOVA nonparametric multiple comparison

Kruskal-Wallis test

Number of families	1					
Number of comparisons	3					
Alpha	0.05					
Dunn's multiple comparisons test	Mean rank diff.	Significant?	Summary	Adjusted P Value		
Untreated 5 vs. CSG	2.1	No	ns	>0.9999		
Untreated 5 vs. TNF-CS(	11.56	Yes	*	0.0152		
CSG vs. TNF-CSG	9.464	Yes	*	0.0139		
Test details	Mean rank 1	Mean rank 2	Mean rank diff.	n1	n2	
Untreated 5 vs. CSG	19.2	17.1	2.1	5	10	
Untreated 5 vs. TNF-CS(	19.2	7.636	11.56	5	11	
CSG vs. TNF-CSG	17.1	7.636	9.464	10	11	

Figure 7D: FACS anaylsis of granzyme B cytotoxic T cells and regulatory CD4 T cells

CD8 T cells granzyme B+

CSG	TNF-CSG 2 ug
0.55	15.2
2.47	16.8
1.62	22.1
5.65	13.2
0.28	29

CD4 T cells granzyme B+

CSG	TNF-CSG 2 ug
2.83	3.92
0.633	3.1
0.526	2.74
1.79	2.89
1.19	2.04

Unpaired t-test

**Table Analyzed**	**CD8 GranzymB**
Column B	TNF-CSG 2 ug
vs.	vs.
Column A	CSG
Unpaired t test	
P value	0.0005
P value summary	***
Significantly different (P < 0.05)?	Yes
One- or two-tailed P value?	Two-tailed
t, df	t=5.701 df=8

**Table Analyze**	**CD4 GranzymB**	
Column B	TNF-CSG 2 ug	
vs.	vs.	
Column A	CSG	
Unpaired t test		
P value	0.0181	
P value summary	*	
Significantly different (P < 0.05)?	Yes	
One- or two-tai	Two-tailed	
t, df	t=2.963 df=8	

CD4 T cells FoxP3+

CSG	TNF-CSG 2 ug
13.1	12.7
15.9	2.17
11.1	10.3
9.73	10.3
12.4	11.9

Unpaired t-test

Table Analyzed	CD4: CD25+ FOXP3+	
Column B	TNF-CSG 2 ug	
vs.	vs.	
Column A	CSG	
Unpaired t test		
P value	0.2045	
P value summary	ns	
Significantly different (P < 0.05)?	No	
One- or two-tailed P valu	Two-tailed	
t, df	t=1.382 df=8	
