# Supplementary material for: Immune‐mediated ECM depletion improves tumour perfusion and payload delivery
Source: EMBO Mol Med. 2019 Nov 11;11(12):e10923. doi: 10.15252/emmm.201910923 (PMC6895610; doi:10.15252/emmm.201910923)
Supplement: Supplementary file 11 — Source Data for Figure 8 [file EMMM-11-e10923-s010.pdf]

Figure 8C: Treatment of 4T1 tumour in BALB/C nude

CD4 (purple)/nuclei (methyl green)

Adoptive transfer of CD4 and CD8 T cells

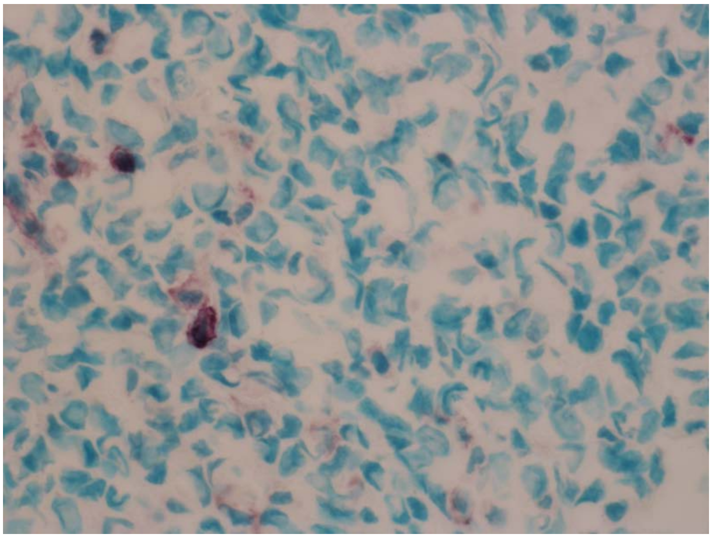

TNF-CSG only

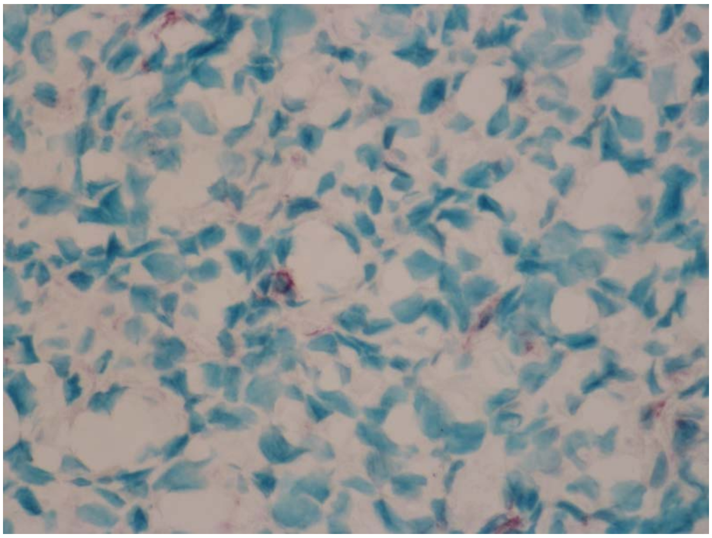

TNF-CSG + CD4 and CD8 T cells

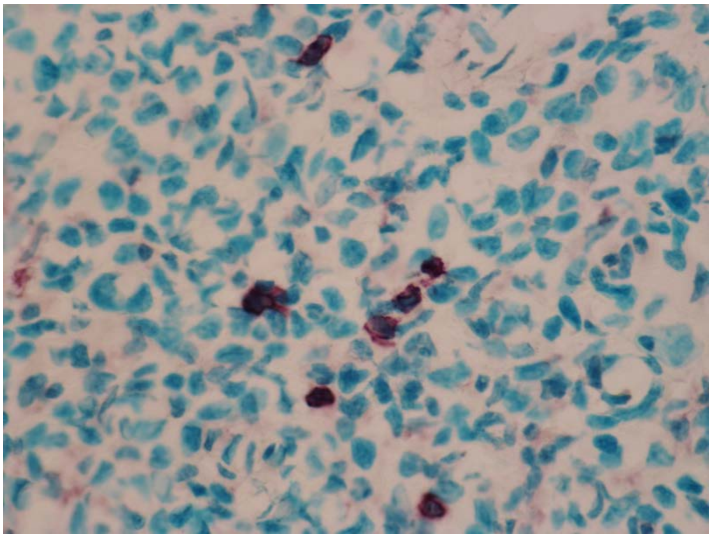

Figure 8C: Treatment of 4T1 tumour in BALB/C nude

CD8 (purple)/nuclei (methyl green)

Adoptive transfer of CD4 and CD8 T cells

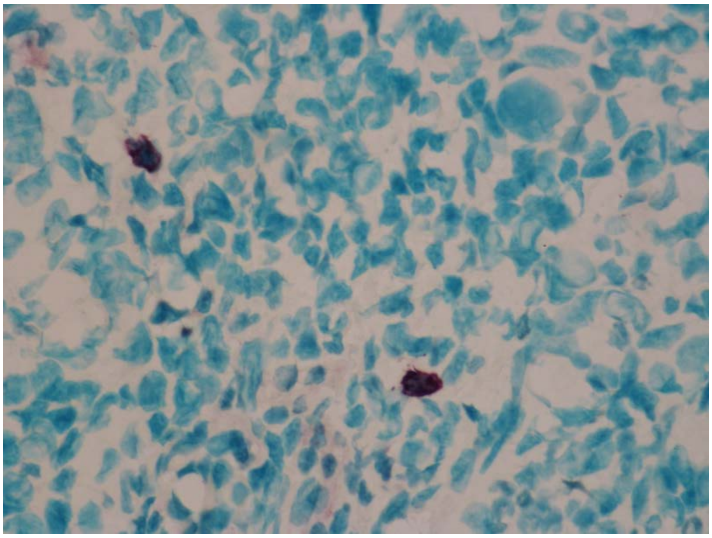

TNF-CSG only

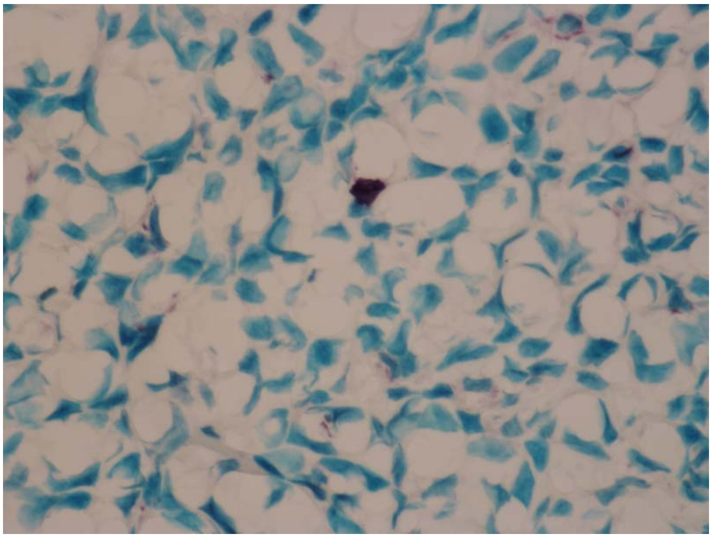

TNF-CSG + CD4 and CD8 T cells

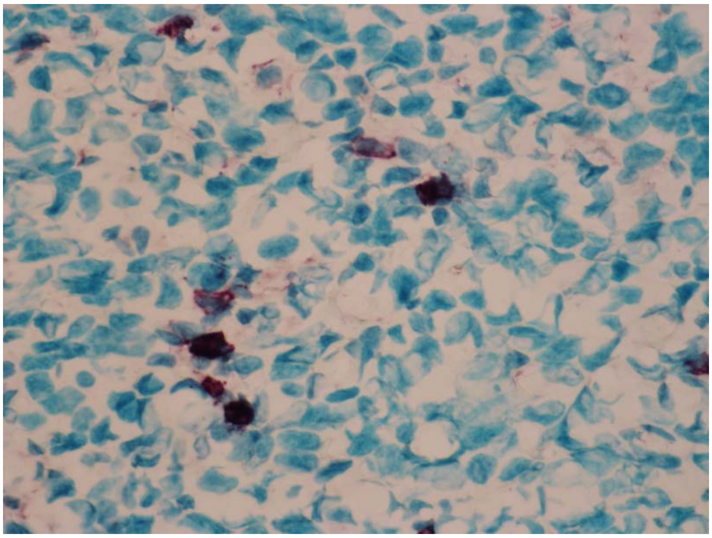

Figure 8D: Treatment of 4T1 tumour in BALB/C nude

Col IV (purple)/nuclei (methyl green)

Untreated

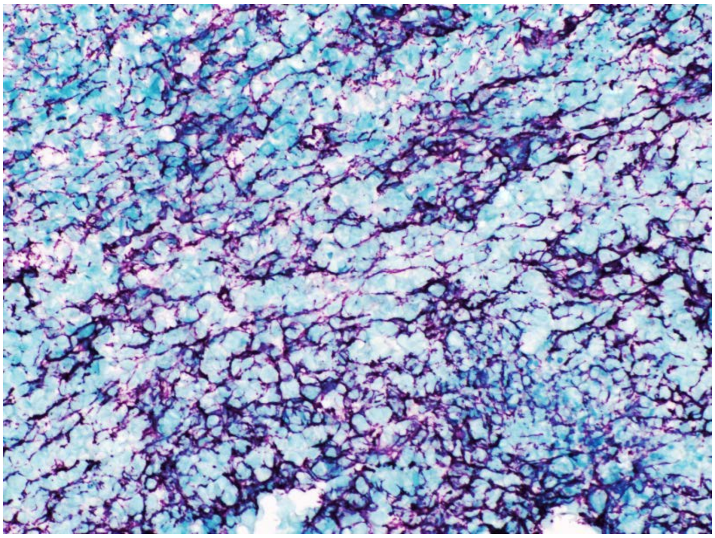

Adoptive transfer of CD4 and CD8 T cells

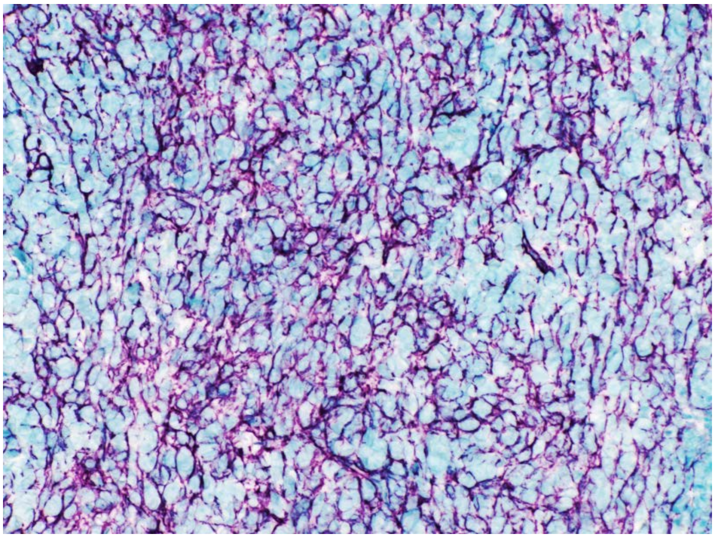

TNF-CSG only

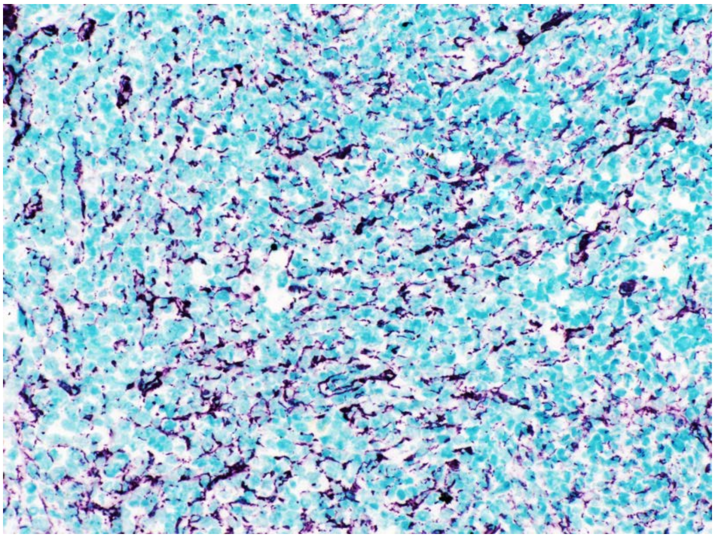

Figure 8D: Treatment of 4T1 tumour in BALB/C nude

Col IV (purple)/nuclei (methyl green)

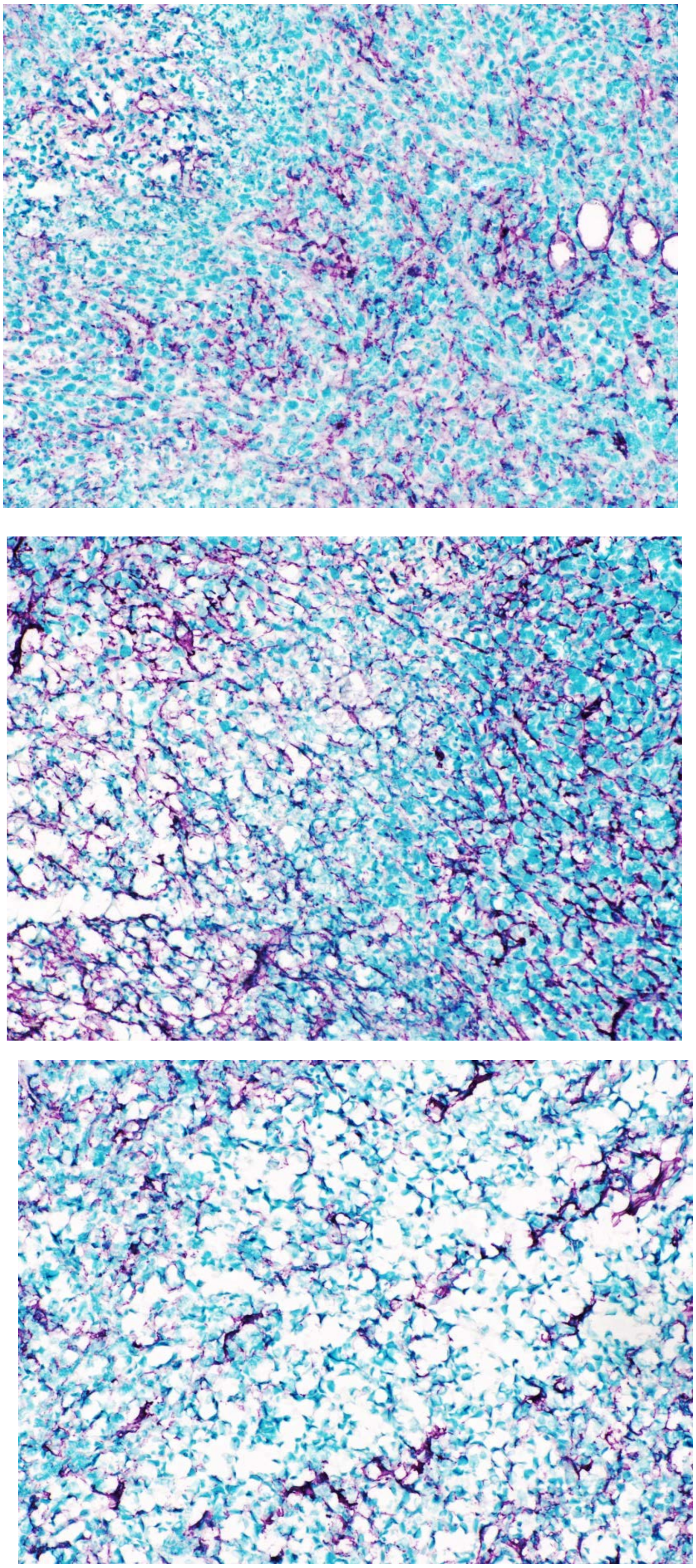

TNF-CSG + CD4 T cells

TNF-CSG + CD8 T cells

TNF-CSG + CD4 and CD8 T cells

Figure 8 B: measured tumour weights

Untreated	CD4+ CD8 transfer	TNF-CSG	CD4 transfer+ TNF-CSG	CD8 transfer + TNF-CSG	CD4+ CD8 transfer + TNF-CSG
0.44	0.35	0.2	0.27	0.3	0.21
0.28	0.35	0.29	0.19	0.21	0.23
0.32	0.36	0.41	0.35	0.27	0.21
0.46	0.34	0.37	0.37	0.41	0.21
0.4	0.37	0.23	0.36	0.36	0.2

One-way ANOVA multiple comparison

Uncorrected Fisher's LSD	Mean Diff.	95.00% CI of diff.	Significant?	Summary	Individual P Value
UNTR vs. CD4+CD8	0.021	-0.06006 to 0.1	No	ns	0.5984
UNTR vs. TNF-CSG	0.075	-0.006064 to 0.	No	ns	0.0683
UNTR vs. TNF-CSG + CD4	0.067	-0.01406 to 0.1	No	ns	0.1011
UNTR vs. TNF-CSG + CD8	0.065	-0.01606 to 0.1	No	ns	0.1112
UNTR vs. TNF-CSG + CD4+CD8	0.163	0.08194 to 0.24	Yes	***	0.0003
CD4+CD8 vs. TNF-CSG	0.054	-0.03067 to 0.1	No	ns	0.2009
CD4+CD8 vs. TNF-CSG + CD4	0.046	-0.03867 to 0.1	No	ns	0.2738
CD4+CD8 vs. TNF-CSG + CD8	0.044	-0.04067 to 0.1	No	ns	0.2947
CD4+CD8 vs. TNF-CSG + CD4+CD8	0.142	0.05733 to 0.22	Yes	**	0.002
TNF-CSG vs. TNF-CSG + CD4	-0.008	-0.09267 to 0.0	No	ns	0.8473
TNF-CSG vs. TNF-CSG + CD8	-0.01	-0.09467 to 0.0	No	ns	0.8098
TNF-CSG vs. TNF-CSG + CD4+CD8	0.088	0.003331 to 0.1	Yes	*	0.0422
TNF-CSG + CD4 vs. TNF-CSG + CD8	-0.002	-0.08667 to 0.0	No	ns	0.9616
TNF-CSG + CD4 vs. TNF-CSG + CD4+CD8	0.096	0.01133 to 0.18	Yes	*	0.0279
TNF-CSG + CD8 vs. TNF-CSG + CD4+CD8	0.098	0.01333 to 0.18	Yes	*	0.025

Figure 8 B: measured tumour volume

Untreated	CD4+ CD8 transfer	TNF-CSG	CD4 transfer+ TNF-CSG	CD8 transfer + TNF-CSG	CD4+ CD8 transfer + TNF-CSG
380.9	242.8	190.2	294.3	222.9	236.8
224.3	291.7	392.9	156.7	182.6	199.1
231.3	330	411.4	252.4	311.4	178.5
368.8	271.3	299.6	289	241	174.6
273.6	305.2	204.5	235.8	232.6	212.4
477.7					

One-way ANOVA multiple comparison

Uncorrected Fisher's LSD	Mean Diff.	95.00% CI of diff.	Significant?	Summary	Individual P Value	
UNTR vs. CD4+CD8	37.9	-48.56 to 124.4	No	ns	0.3753	
UNTR vs. TNF-CSG	26.38	-60.08 to 112.8	No	ns	0.5355	
UNTR vs. TNF-CSG + CD4	80.46	-6.002 to 166.9	No	ns	0.0668	
UNTR vs. TNF-CSG + CD8	88	1.538 to 174.5	Yes	*	0.0464	
UNTR vs. TNF-CSG + CD4+CD8	125.8	39.36 to 212.3	Yes	**	0.0061	
CD4+CD8 vs. TNF-CSG	-11.52	-101.8 to 78.79	No	ns	0.7949	
CD4+CD8 vs. TNF-CSG + CD4	42.56	-47.75 to 132.9	No	ns	0.341	
CD4+CD8 vs. TNF-CSG + CD8	50.1	-40.21 to 140.4	No	ns	0.264	
CD4+CD8 vs. TNF-CSG + CD4+CD8	87.92	-2.387 to 178.2	No	ns	0.0559	
TNF-CSG vs. TNF-CSG + CD4	54.08	-36.23 to 144.4	No	ns	0.2289	
TNF-CSG vs. TNF-CSG + CD8	61.62	-28.69 to 151.9	No	ns	0.1722	
TNF-CSG vs. TNF-CSG + CD4+CD8	99.44	9.133 to 189.7	Yes	*	0.0322	
TNF-CSG + CD4 vs. TNF-CSG + CD8	7.54	-82.77 to 97.85	No	ns	0.8649	
TNF-CSG + CD4 vs. TNF-CSG + CD4+CD8	45.36	-44.95 to 135.7	No	ns	0.3108	
TNF-CSG + CD8 vs. TNF-CSG + CD4+CD8	37.82	-52.49 to 128.1	No	ns	0.3966	

Figure 8C: Counts of immune cells/field of tumour area

	Untreated			CD4+ CD8 transfer			TNF-CSG			CD4 transfer+ TNF-CSG			CD8 transfer + TNF-CSG			CD4+ CD8 transfer + TNF-CSG		
CD4	3	0	2	6	1	2	2	2	4	9	11	11	3	0	3	8	15	11
CD8	4	1	0	5	1	1	5	2	3	5	5	2	8	8	6	20	11	8

One-way ANOVA multiple comparison

Number of families	1
Number of comparisons per family	15
Alpha	0.05

CD4 T cells

Tukey's multiple comparisons test	Mean Diff.	95.00% CI of diff.	Significant?	Summary	Adjusted P Value
UNTR vs. CD4+CD8	-1.333	-7.187 to 4.52	No	ns	0.9685
UNTR vs. TNF-CSG	-1	-6.854 to 4.854	No	ns	0.991
UNTR vs. TNF-CSG + CD4	-8.667	-14.52 to -2.81	Yes	**	0.0034
UNTR vs. TNF-CSG + CD8	-0.3333	-6.187 to 5.52	No	ns	>0.9999
UNTR vs. TNF-CSG + CD4+CD8	-9.667	-15.52 to -3.81	Yes	**	0.0014
CD4+CD8 vs. TNF-CSG	0.3333	-5.52 to 6.187	No	ns	>0.9999
CD4+CD8 vs. TNF-CSG + CD4	-7.333	-13.19 to -1.48	Yes	*	0.0119
CD4+CD8 vs. TNF-CSG + CD8	1	-4.854 to 6.854	No	ns	0.991
CD4+CD8 vs. TNF-CSG + CD4+CD8	-8.333	-14.19 to -2.48	Yes	**	0.0046
TNF-CSG vs. TNF-CSG + CD4	-7.667	-13.52 to -1.81	Yes	**	0.0087
TNF-CSG vs. TNF-CSG + CD8	0.6667	-5.187 to 6.52	No	ns	0.9986
TNF-CSG vs. TNF-CSG + CD4+CD8	-8.667	-14.52 to -2.81	Yes	**	0.0034
TNF-CSG + CD4 vs. TNF-CSG + CD8	8.333	2.48 to 14.19	Yes	**	0.0046
TNF-CSG + CD4 vs. TNF-CSG + CD4+CD8	-1	-6.854 to 4.854	No	ns	0.991
TNF-CSG + CD8 vs. TNF-CSG + CD4+CD8	-9.333	-15.19 to -3.48	Yes	**	0.0018

CD8 T cells

Tukey's multiple comparisons test	Mean Diff.	95.00% CI of diff.	Significant?	Summary	Adjusted P Value	
UNTR vs. CD4+CD8	-0.6667	-8.995 to 7.662	No	ns	0.9998	A-B
UNTR vs. TNF-CSG	-1.667	-9.995 to 6.662	No	ns	0.9819	A-C

UNTR vs. TNF-CSG + CD4	-2.333	-10.66 to 5.995	No	ns	0.9278	A-D
UNTR vs. TNF-CSG + CD8	-5.667	-14 to 2.662	No	ns	0.2709	A-E
UNTR vs. TNF-CSG + CD4+CD8	-11.33	-19.66 to -3.00[illegible]	Yes	**	0.0065	A-F
CD4+CD8 vs. TNF-CSG	-1	-9.329 to 7.329	No	ns	0.9983	B-C
CD4+CD8 vs. TNF-CSG + CD4	-1.667	-9.995 to 6.662	No	ns	0.9819	B-D
CD4+CD8 vs. TNF-CSG + CD8	-5	-13.33 to 3.329	No	ns	0.3874	B-E
CD4+CD8 vs. TNF-CSG + CD4+CD8	-10.67	-19 to -2.338	Yes	*	0.0102	B-F
TNF-CSG vs. TNF-CSG + CD4	-0.6667	-8.995 to 7.662	No	ns	0.9998	C-D
TNF-CSG vs. TNF-CSG + CD8	-4	-12.33 to 4.329	No	ns	0.6058	C-E
TNF-CSG vs. TNF-CSG + CD4+CD8	-9.667	-18 to -1.338	Yes	*	0.0201	C-F
TNF-CSG + CD4 vs. TNF-CSG + CD8	-3.333	-11.66 to 4.995	No	ns	0.7568	D-E
TNF-CSG + CD4 vs. TNF-CSG + CD4+CD8	-9	-17.33 to -0.67	Yes	*	0.0317	D-F
TNF-CSG + CD8 vs. TNF-CSG + CD4+CD8	-5.667	-14 to 2.662	No	ns	0.2709	E-F

Figure 8D: % Col-IV staining/field of tumour area

Untreated	CD4+ CD8 transfer	TNF-CSG	CD4 transfer+ TNF-CSG	CD8 transfer + TNF-CSG	CD4+ CD8 transfer + TNF-CSG
28.6	27.7	12.2	8.1	6.8	10.5
35.3	32.5	19.4	13.8	20.4	8.3
31.5	31.6	25.4	12.3	17.7	13.5
31.1	25.9	20.1	16.7	14.2	3.1
22.7	27.1	16.8	12.7	14.6	9.3

One-way ANOVA multiple comparison

Number of families	1
Number of comparisons per family	15
Alpha	0.05

Tukey's multiple comparisons test	Mean Diff.	95.00% CI of d	Significant?	Summary	Adjusted P Value
UNTR vs. CD4+CD8	0.88	-7.245 to 9.005	No	ns	0.9994
UNTR vs. TNF-CSG	11.06	2.935 to 19.18	Yes	**	0.0037
UNTR vs. TNF-CSG + CD4	17.12	8.995 to 25.24	Yes	****	<0.0001
UNTR vs. TNF-CSG + CD8	15.1	6.975 to 23.22	Yes	****	<0.0001
UNTR vs. TNF-CSG + CD4+CD8	20.9	12.78 to 29.02	Yes	****	<0.0001
CD4+CD8 vs. TNF-CSG	10.18	2.055 to 18.3	Yes	**	0.0084
CD4+CD8 vs. TNF-CSG + CD4	16.24	8.115 to 24.36	Yes	****	<0.0001
CD4+CD8 vs. TNF-CSG + CD8	14.22	6.095 to 22.34	Yes	***	0.0002
CD4+CD8 vs. TNF-CSG + CD4+CD8	20.02	11.9 to 28.14	Yes	****	<0.0001
TNF-CSG vs. TNF-CSG + CD4	6.06	-2.065 to 14.18	No	ns	0.2302
TNF-CSG vs. TNF-CSG + CD8	4.04	-4.085 to 12.16	No	ns	0.6448
TNF-CSG vs. TNF-CSG + CD4+CD8	9.84	1.715 to 17.96	Yes	*	0.0114
TNF-CSG + CD4 vs. TNF-CSG + CD8	-2.02	-10.14 to 6.105	No	ns	0.9702
TNF-CSG + CD4 vs. TNF-CSG + CD4+CD8	3.78	-4.345 to 11.9	No	ns	0.7042
TNF-CSG + CD8 vs. TNF-CSG + CD4+CD8	5.8	-2.325 to 13.92	No	ns	0.271
